# Genome-wide profiling of the PIWI-interacting RNA-mRNA regulatory networks in epithelial ovarian cancers

**DOI:** 10.1371/journal.pone.0190485

**Published:** 2018-01-10

**Authors:** Garima Singh, Jyoti Roy, Pratiti Rout, Bibekanand Mallick

**Affiliations:** RNAi and Functional Genomics Laboratory, Department of Life Science, National Institute of Technology – Rourkela, Odisha, India; West China Second Hospital, Sichuan University, CHINA

## Abstract

PIWI-interacting (piRNAs), ~23–36 nucleotide-long small non-coding RNAs (sncRNAs), earlier believed to be germline-specific, have now been identified in somatic cells, including cancer cells. These sncRNAs impact critical biological processes by fine-tuning gene expression at post-transcriptional and epigenetic levels. The expression of piRNAs in ovarian cancer, the most lethal gynecologic cancer is largely uncharted. In this study, we investigated the expression of PIWILs by qRT-PCR and western blotting and then identified piRNA transcriptomes in tissues of normal ovary and two most prevalent epithelial ovarian cancer subtypes, serous and endometrioid by small RNA sequencing. We detected 219, 256 and 234 piRNAs in normal ovary, endometrioid and serous ovarian cancer samples respectively. We observed piRNAs are encoded from various genomic regions, among which introns harbor the majority of them. Surprisingly, piRNAs originated from different genomic contexts showed the varied level of conservations across vertebrates. The functional analysis of predicted targets of differentially expressed piRNAs revealed these could modulate key processes and pathways involved in ovarian oncogenesis. Our study provides the first comprehensive piRNA landscape in these samples and a useful resource for further functional studies to decipher new mechanistic views of piRNA-mediated gene regulatory networks affecting ovarian oncogenesis. The RNA-seq data is submitted to GEO database (GSE83794).

## Introduction

Ovarian cancer (OCa) is the most lethal gynecologic cancer which has emerged as a leading cause of mortality among women over the past years and responsible for 0.14 million deaths annually worldwide [1, 2]. OCa is heterogeneous in origin and categorized primarily into three histologic types- epithelial ovarian cancer (EOCa) (60%), germ cells tumors (30%) and sex-cord stromal tumors (8%) [2]. Among these, EOCa is the most prevalent neoplasm with less than 30% survival rate [3]. Further, there are several morphological subtypes of EOCa which include serous (70%), endometrioid (10%), clear cell (10%), low-grade serous (5%), mucinous (3%), undifferentiated and unclassified [3, 4]. Among OCa types, endometrioid ovarian cancer (ENOCa) and serous ovarian cancer (SOCa) of EOCa are the frequently observed and highly lethal cancers [2].

Several studies have proposed that alteration in signaling pathways, mutations in various signaling molecules and deregulations of genes with tumorigenic potential are the key causative factors of OCa [5, 6]. Despite these discoveries, there is still a lack of clinical utility such as diagnostic markers and precise knowledge about the mechanisms of deregulated gene complexities in the neoplastic pathways which hinder to enhance the overall survival rate of OCa patients. To explore the genomic complexity and to fill the void of clinical utility in OCa, several biomarkers such as CA125, human epididymis protein-4(HE4), decoy receptor-3 (DcR-3), ERB2, and EGFR, etc. have been proposed, but all of them are either non-specific or insensitive [7]. Further, non-coding RNAs (ncRNAs), especially microRNAs (miRNAs) have been well studied and proposed as prognostic or predictive tools in cancer because of their tissue-specific nature of expression and higher abundance than that of protein-coding mRNAs [8–10]. Moreover, a strong correlation between ncRNAs and cancers have been reported wherein ncRNAs act as potent regulators of gene expression at transcriptional and post-transcriptional levels and are often de-regulated in cancers [11–14].

More recently, piRNAs, a newly discovered class of ncRNA has garnered keen attention due to their diversified emerging roles in the human genome [15]. These ncRNAs earlier thought to be germline-specific are about ~23–36 nucleotides (nts) in length and interact with PIWI protein to form piRISC complex (piRNA-induced silencing complex) that regulates mechanistic RNA-based inhibition of transposable elements (TEs) in germlines [16, 17]. Apart from regulation of TEs, piRNAs can target non-transposable elements such as protein-coding mRNAs and modulate their expression not only in germlines but also in somatic cells by a mechanism similar to that of miRNAs [18, 19].

The roles of piRNAs have been documented in some of the human cancers [20–24]. However, no information is available to date on the expression of piRNAs and their role in OCa carcinogenesis. Therefore, we performed genome-wide piRNA profiling in tissues of healthy ovary and two most prevalent lethal histological subtypes of EOCa (ENOCa, SOCa) by performing next-generation small RNA sequencing (RNAs of 16–40 nts) after confirming the expression of PIWIL mRNAs and proteins in these tissues by qRT-PCR, and western blotting techniques. This study identified an extensive catalog of piRNAs expressed in individual samples as well as differentially expressed piRNAs in EOCa subtypes with respect to normal ovarian sample as a control. The impact of differentially expressed piRNAs on de-regulated target mRNAs in both the subtypes of EOCa was studied to advance our understanding of ovarian tumorigenesis mediated by this young class of small ncRNAs. This study holds great promise for improving diagnosis and treatment of OCa.

## Materials and methods

### Collection of tissue samples

The tissue samples from ovarian cancer patients and the healthy donor was surgically resected and immediately placed into RNAlater solution (Invitrogen) at room temperature. These samples were collected from neighboring hospitals (Ispat General Hospital, Rourkela, Odisha and HCG Panda Cancer Hospital, Cuttack, Odisha) in the state. The median age of the donors was 60 years. The resected ovarian cancer tissues were characterized as ENOCa and SOCa subtypes from the tissue biopsy. The samples were stored at -80°C until next-generation sequencing, and other experiments were performed.

### Ethics

All experiments were carried out in accordance with the relevant guidelines and regulations, and the study was approved by the Institutional Ethical Committee (IEC) of NIT Rourkela, Odisha, India. All sample donors have provided written informed consent to this study for research purpose.

### RNA isolation and integrity

RNA was isolated from all three tissue samples (ENOCa, SOCa, and normal ovary) using RNeasy Mini kit (Qiagen, USA) according to the manufacturer’s instructions. The concentration and purity of isolated RNA was measured using NanoDrop spectrophotometer (Thermo Scientific) by measuring absorbance at 260 nm and 280 nm. RNA integrity of the samples was measured using Bioanalyzer chip (Agilent Technologies).

### Real-time quantitative PCR (qPCR)

The primers (both forward and reverse) were designed for four human *PIWIL* mRNAs (*HIWI*, *HILI*, *PIWIL3*, *HIWI2*) using PrimerQuest (IDT) to amplify *PIWIL* genes. 2 μg of total RNA was used for the synthesis of cDNA using the First-strand cDNA synthesis kit (Invitrogen) following manufacturer’s protocol. 20 ng of the cDNA per reaction was used for real-time quantification using Maxima-SYBR green qPCR-MasterMix (2X) (ThermoScientific) on Real-Time qPCR system (Eppendorf). All reactions were run in triplicates. The expression levels of four *PIWIL* mRNAs were normalized to the endogenous control, *ACTB* (β-actin). We also adopted the same approach to quantify the piRNA targets such as NUDT4, MTR, EIF2S3, and MPHOSPH8 in ENOCa as well as LIAS, PLEKHA5 and ACTR10 in SOCa.

Further, piRNA was isolated from all three tissue samples (ENOCa, SOCa, and normal ovary) using mirVana miRNA isolation kit (Ambion, USA) according to the manufacturer’s protocol. The kit enables isolation and enrichment of small RNA-containing total RNAs of <200 nts. The primers of selected piRNAs (piR-52207, piR-33733) were designed using their sequences available at NCBI and were synthesized by Integrated DNA Technologies (IDT). cDNA was prepared using miScript Reverse transcription kit (Qiagen). The quantification of piRNAs was done using miScript SYBR Green PCR kit (Qiagen) considering small nucleolar RNA U6 an endogenous control for normalization.

### Western blot

Protein was extracted from the homogenized tissues using RIPA buffer and estimated using BCA Protein Assay Kit (Thermo Fisher Scientific). The expression of PIWIL protein was evaluated by sodium dodecyl sulphate (SDS) acrylamide gel electrophoresis and immunoblotting of total protein extracts using rabbit anti-PIWIL1 (ab12337, Abcam), rabbit anti-PIWIL2 (ab26408, Abcam), rabbit anti-PIWIL4 (ab111714, Abcam), mouse anti β-actin (ab6276, Abcam) and goat anti-rabbit IgG H&L (HRP) (ab97051, Abcam).

### Library preparation and small RNA sequencing

1μg of high-quality total RNA with optimal RIN (RNA Integrity Number) values was used for library preparation as per Illumina TruSeq small RNA library protocol. Genotypic Technology, Bangalore, India carried out small RNA sequencing on Illumina Next-Seq 500 platform. Small RNAs were size fractionated in the range of 16–40 nucleotides in length and were ligated to the adapter at 3^/^ end. The ligation product was used as template in the reverse transcription reaction and amplified by 11 cycles of PCR. The purified PCR products were then subjected to next-generation sequencing to generate small RNA reads. The quality of generated reads was assessed using FastQC, a quality control tool.

### Mapping of reads to hg19 human genome and annotations

The reads after removal of 3^/^ adapters by Cutadapt were aligned to the human reference genome and transcriptomes using Bowtie, an efficient and widely used genome-scale alignment tool [25]. The uniquely mapped reads with no more than one mismatch in the alignment were considered for their annotations. The human reference sequences (hg19) used for this purpose were downloaded from the UCSC genome browser.

The uniquely mapped reads showing length distribution within the range of 16–40 nts were analyzed using iMir [26] and mirTools2.0 [27] to predict known and novel piRNAs as well as miRNAs and their differential expression with respect to normal ovarian tissue as a control. The annotations of genomic regions of the detected piRNAs and miRNAs were done by aligning them to the coordinates of protein-coding genes (PCGs) (5^/^UTR, CDS, 3^/^UTR), repeats, pseudogenes, introns, small ncRNAs and lncRNAs using in-house programs. These individual annotation track files used for predicting the genomic origin of the piRNAs were downloaded from UCSC FTP site.

### Identification of differentially expressed piRNAs

The differential expression analysis of the small RNAs was performed using DESeq of Bioconductor [28] to obtain a set of piRNAs significantly up- or down-regulated in each of the two EOCa types with respect to normal ovary as a control. The expression levels (up/down) of piRNAs were considered significant if the fold-change (FC) is ≥1.5 and p-value is ≤0.05.

### Identification of piRNA clusters

We searched for the piRNA clusters that are considered as the hotspot of their origin within a genome. We designated piRNAs as part of a cluster when at least 10 different piRNAs are located on a chromosome on a scanning window of length 20 kilobase (kb) and a window shift of 1 kb.

### Evolutionary conservation of piRNAs

The conservation score of each piRNA across 100 vertebrate species was computed by averaging the PhastCons conservation scores of each nucleotides which are computed by the PhastCons program from the alignment of 100 vertebrate species. The PhastCons score in big Wig track format and bigwig Average OverBed tool downloaded from UCSC genome browser (http://genome.ucsc.edu/) through FTP was used to compute the conservation score of each piRNA. The boxplot analysis of conservation patterns of piRNAs from different genomic regions was performed using R to see the enrichment of conservation scores of all piRNAs originated from respective genomic locations.

### Identification of differentially expressed mRNAs in ENOCa and SOCa

We downloaded gene expression profiles of ENOCa and SOCa from Gene Expression Omnibus database (GEO: GSE6008, HG-U133A Affymetrix platform) [29]. The GSE6008 microarray dataset is comprised of 37 samples of ENOCa, 41 samples of SOCa and 4 samples of normal ovary. The expression analysis of these samples was performed in GeneSpring GX 12.6 software (Agilent Technologies). The raw data samples were normalized using Robust Multichip Averaging (RMA) algorithm wherein quantile normalization with median of all samples taken for baseline transformation were considered followed by summarization of probe sets by percentile shift algorithm. The probe sets having intensities less than 20 percentile were excluded. The probe sets were then subjected to statistical analysis using unpaired t-test and Benjamini-Hochberg false discovery rate multiple-testing corrections at a rate of 0.05. Finally, the genes differentially expressed in ENOCa and SOCa compared to normal ovarian sample were obtained by exercising a fold-change ≥2.0 with p-value ≤0.05.

### Identification of mRNAs containing retrotransposable elements (RTEs)

We used Repeat Masker program [30] to screen down the differentially expressed genes that contain retrotransposable elements (RTEs) within them. The RTEs are known to be the prime target regions of piRNAs. Briefly, we uploaded the sequences of differentially expressed genes of ENOCa and SOCa individually to the Web server of RepeatMasker and obtained sets of genes that harbor any repeat elements. We excluded the genes that harbor simple repeats, and low complexity regions repeat from further analysis. The genomic locations of RTEs obtained from the RepeatMasker results were used in subsequent steps to filter piRNA targets by checking whether binding site region is falling within RTE.

### Identification of potential piRNA targets and functional analysis

The potential targets for up-regulated piRNAs were identified by searching for target sites within 5^/^UTR, CDS and 3^/^UTRs of downregulated RTE-containing mRNAs with an alignment score (sc ≥170) and energy (en ≤ -20Kcal/mol) proposed by Hashim et al. [21] using miRanda Algorithm. The predicted targets were analyzed using MetaCore (Thomson Reuters) to identify enriched biological processes, disease functions, networks, pathways and upstream regulators. The above-enriched results were screened further to unveil the involvement of target genes in EOCa regulated by DE-piRNAs. We filtered and considered only those target genes that are enriched in cancer-related functions or processes but do not have any reported upstream regulator(s). The target genes were then scooped out that harbor binding sites within RTEs and showed watson-crick base pairing to primary (2–11 nts) and secondary (12–21 nts) seed regions of piRNAs as proposed by Goh et al. [31] with slight modification by allowing up to one G:U pair and/or a mismatch. Among these, target genes appearing in either canonical pathway(s) or network(s) are only considered to evaluate and discuss the impact of piRNAs targeting these genes and their possible consequences on tumorigenesis of EOCa. The excluded targets enriched in only cancer-related functions/processes that showed binding sites of piRNAs as per Hashmi et al. can also be important to explore their role in EOCa in future.

## Results and discussion

### *PIWI* orthologues- widely expressed in human EOCa

**Fig 1:**
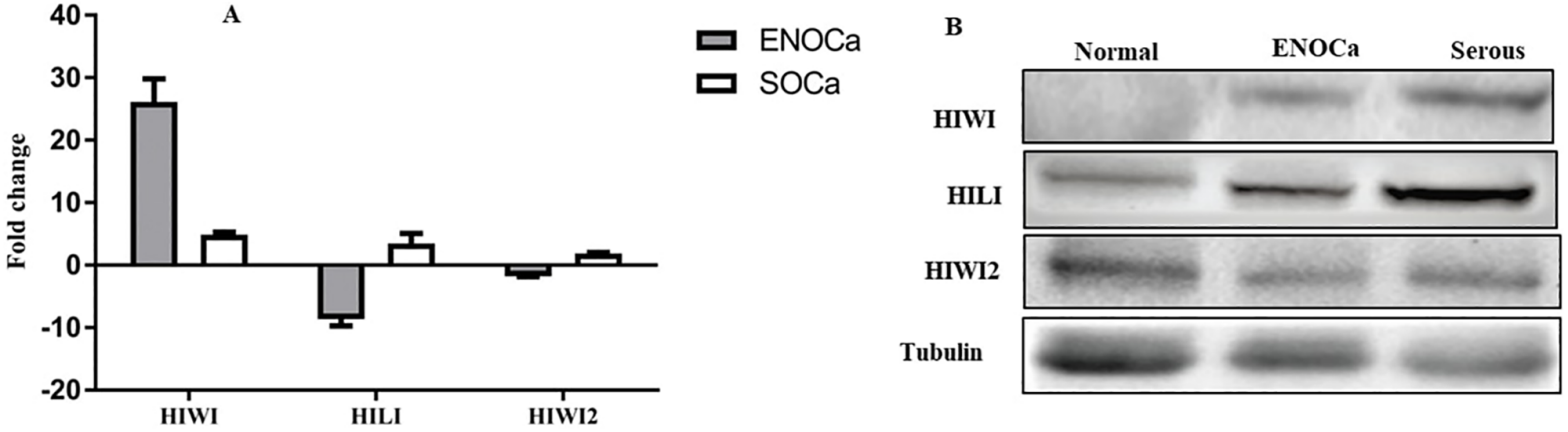
Expression profile of PIWIL genes and proteins in human normal ovarian and cancer tissues. A. The relative expression of thee PIWIL mRNAs analyzed by qRT-PCR analysis. The amount of PIWIL mRNAs was normalized to the endogenous control, β-actin mRNA. The fold-change was calculated based on the ratio of the normalized values of the ENOCa and SOCa to that of normal ovary. B. Western blot of three PIWIL proteins in normal ovary, ENOCa and SOCa.

The biogenesis, as well as the functionality of piRNAs, are linked with expression of Piwi-like (Piwil) genes producing *PIWIL* proteins, the key members of the piRNA pathways. In human, four PIWIL genes (HIWI, HILI, PIWIL3, and HIWI2) are reported which are initially found in testis. Recent studies demonstrated some of these PIWILs, such as HIWI and HILI are highly expressed in a variety of human cancers [32]. To investigate the presence of active piRNA pathways in the EOCa, we surveyed the expression of PIWIL mRNAs in ENOCa, SOCa as well as in normal ovary by performing real-time qPCR. The relative expression of three human PIWI orthologues, *HIWI*, *HILI*, and *HIWI2* except *PIWIL3* was detected from qRT-PCR study in both the cancer tissues and healthy tissue [33] (Fig 1A). All of these mRNAs are upregulated in SOCa compared to normal ovary, whereas HIWI is highly upregulated and HILI is downregulated in ENOCa. The overexpression of *HILI* is known to be correlated with the etiology of cancers of the colon, breast, and cervix [34, 35]. We speculate that the differential expression of these genes might be involved in oncogenicity of SOCa and ENOCa similar to that in other cancers. The expression of these PIWIL orthologues at protein level was also determined by Western Blot **(**Fig 1B**)**.

### Small RNA-seq catalog in human ovary and EOCa

**Fig 2:**
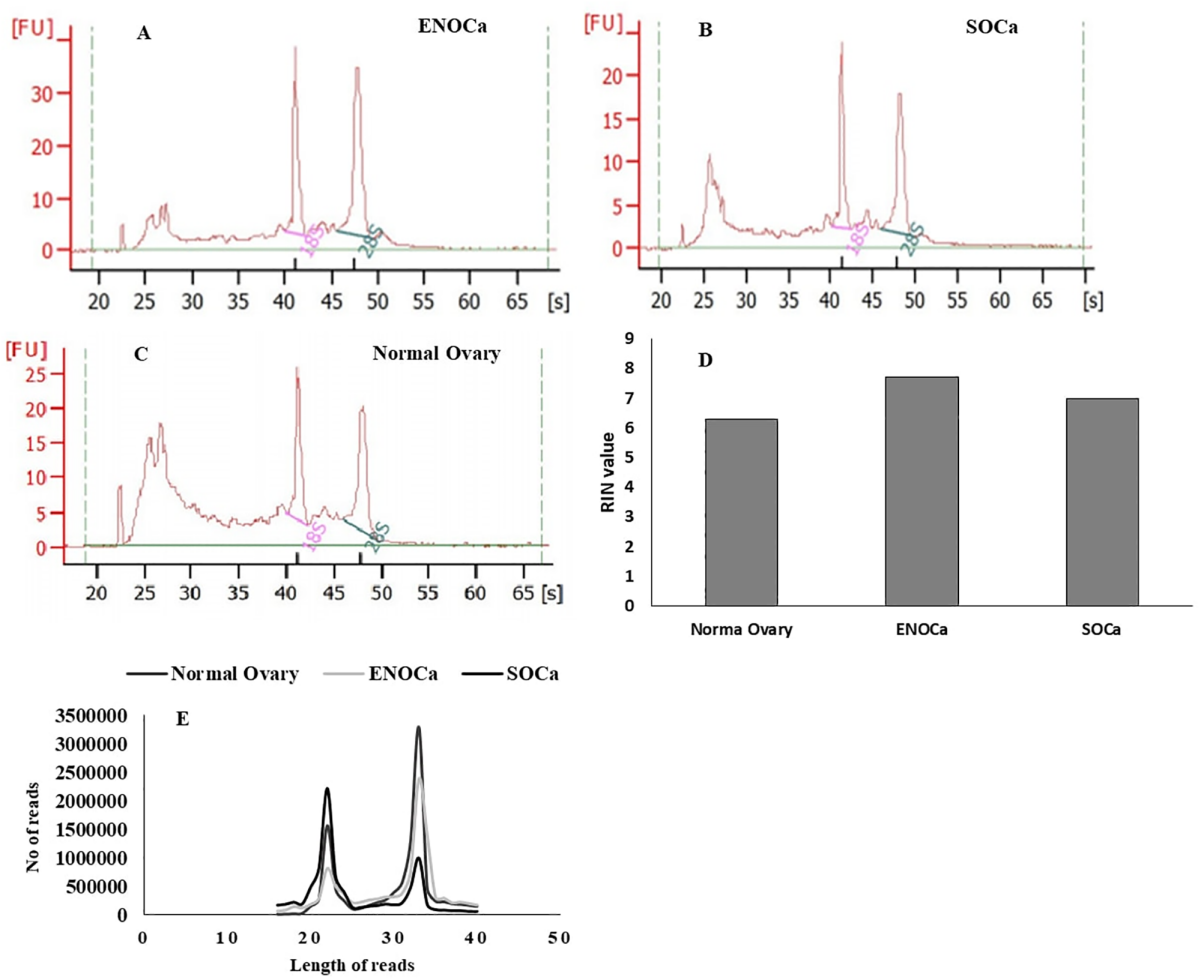
QC analysis of tissue samples of ENOCa, SOCa and normal ovary for small RNA sequencing and generation of reads. Agilent RNA Bioanalyzer profile of A. ENOCa; B. SOCa; C. normal ovary; D. RIN values revealing small RNA intactness optimal for sequencing; E. Number of trimmed reads of 16–40 nts generated from each sample type.

We performed next-generation sequencing of small RNAs of 16–40 nucleotides extracted from three samples (ENOCa, SOCa and Normal) to identify the repertoire of piRNome in human EOCa. The samples with optimal RIN values (>6) measured by Agilent RNA Bioanalyzer (Fig 2A–2D)were only considered for the sequencing. The sequencing strategy generated ~15 million high-quality reads for each sample as evident from QC analysis. The adapter-trimmed high-quality reads were aligned to the human genome (hg19). Among these, 11991579 (89.63%) reads from the normal ovary, 12718957 (88.87%) reads from ENOCa, and 9639236 (80.45%) reads from SOCa in the range of 16 to 40 nucleotides mapped perfectly to hg19 human genome which comprise of both miRNAs and piRNAs (Fig 2E). The raw reads from these samples have been submitted to the GEO database of NCBI (accession number: GSE83794).

**Fig 3:**
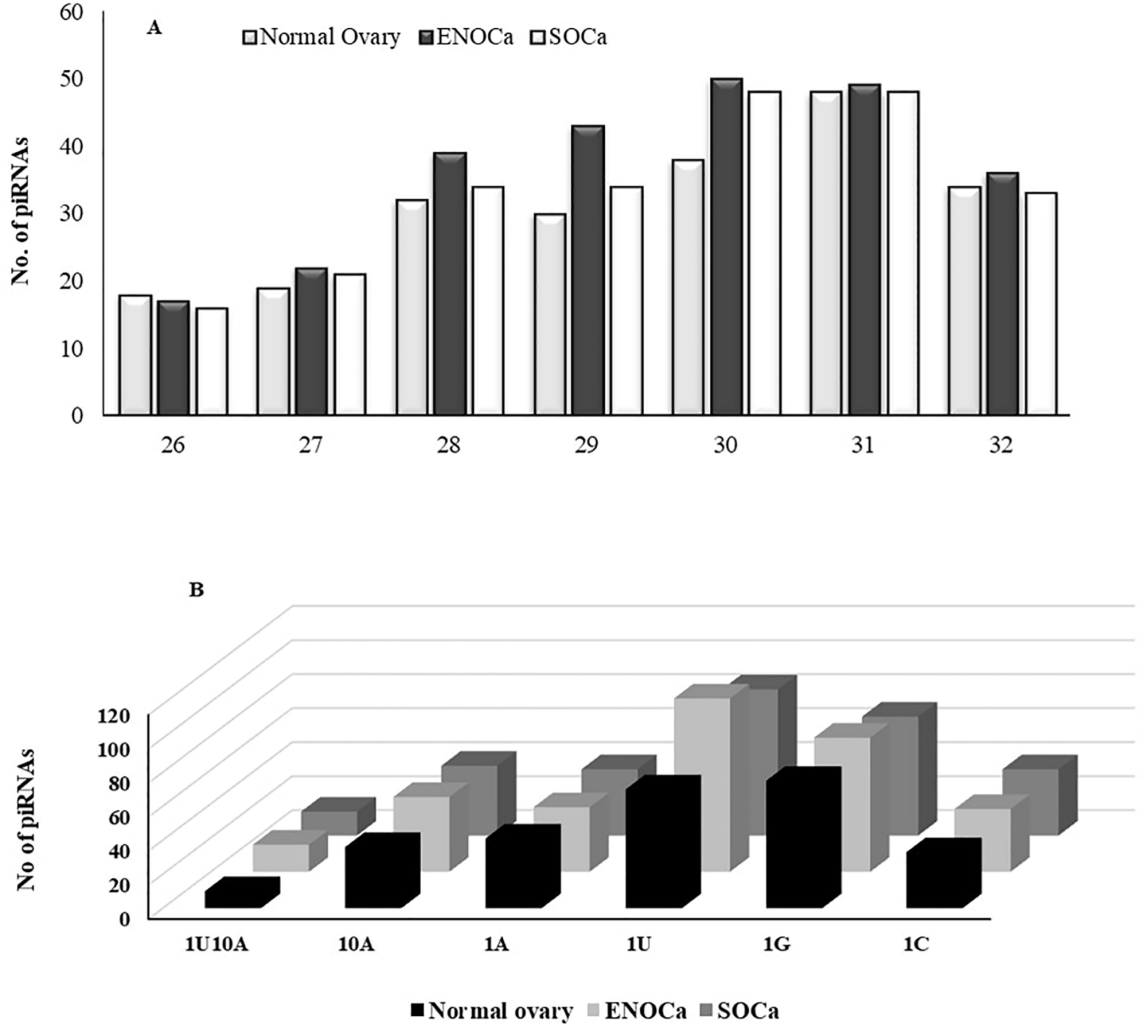
Characteristic properties of piRNAs identified in ENOCa, SOCa and normal ovary. A. Length and B. nucleotide bias observed among the piRNAs identified in each samples.

The analysis of reads aligned to hg19 genome identified a total of 219, 256, and 234 piRNAs in normal ovary, ENOCa, and SOCa respectively (S1 Table); whereas the average number of known miRNAs detected in each sample was 480 (S2 Table). The piRNAs from each sample exhibited varied length distribution between 26–32 nts with specific nucleotide bias at 1^st^ and 10^th^ position (Fig 3A and 3B). Further, the reads from each sample that did not align with annotated piRNAs or miRNAs were processed through piRNA and miRNA predictor individually to detect un-annotated small RNAs, otherwise termed as novel piRNA-likes and miRNA-likes.

### Genomic architecture of piRNome in human ovary, ENOCa, and SOCa

**Fig 4:**
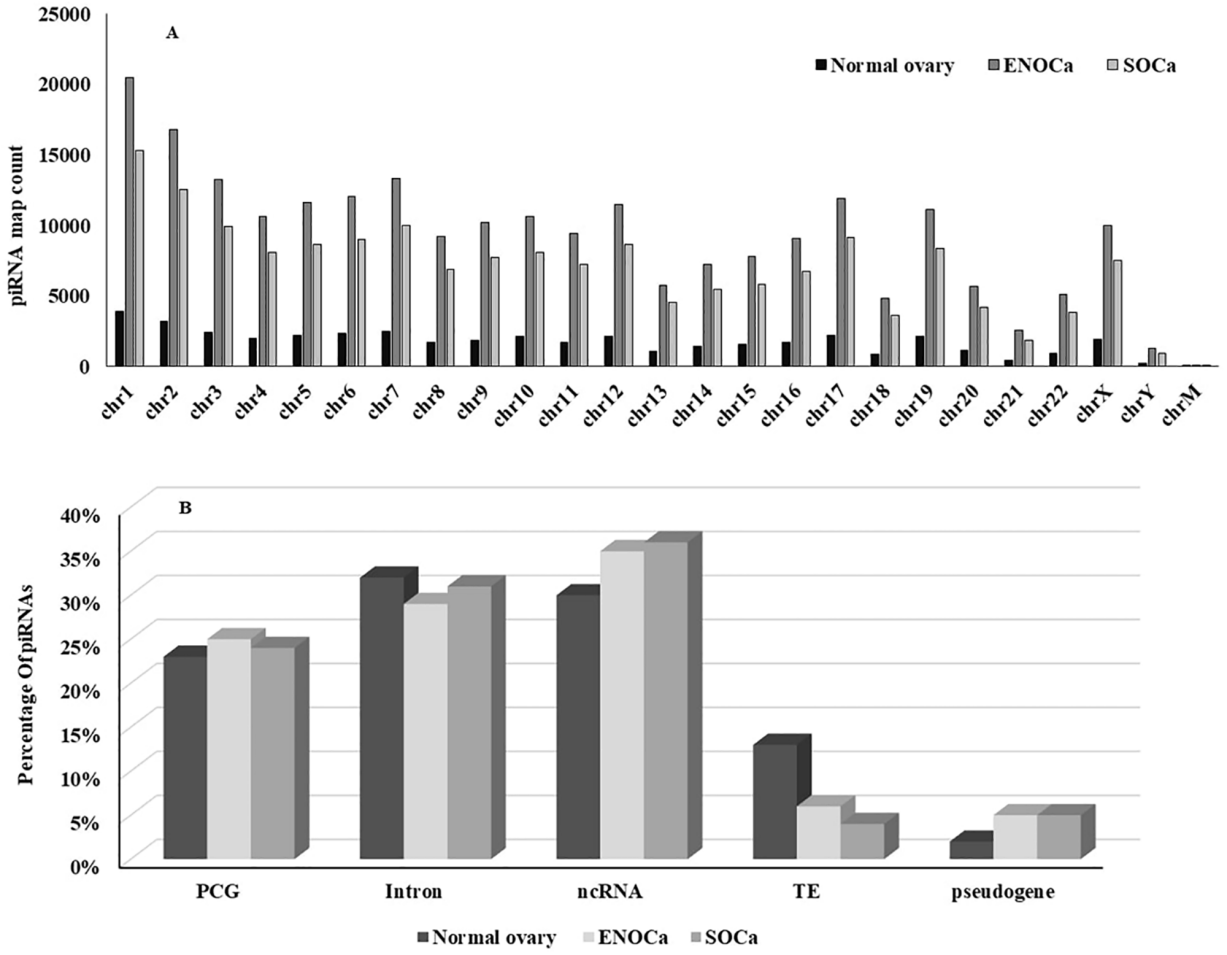
The chromosomal origin and genomic contexts of piRNAs in human genome. A. Origin of piRNAs on all human chromosomes in three samples; B. piRNAs mapped to various genomic contexts of human genome.

Similar to the other ncRNAs, piRNAs are also seen to be originated from various genic and non-genic regions of all human chromosomes including the mitochondrial chromosome (Fig 4A) in all three samples. The piRNAs originated from different genomic regions (Fig 4B) demonstrating different characteristic features and functions are discussed below separately and concisely.

#### PCG-derived piRNAs

**Fig 5:**
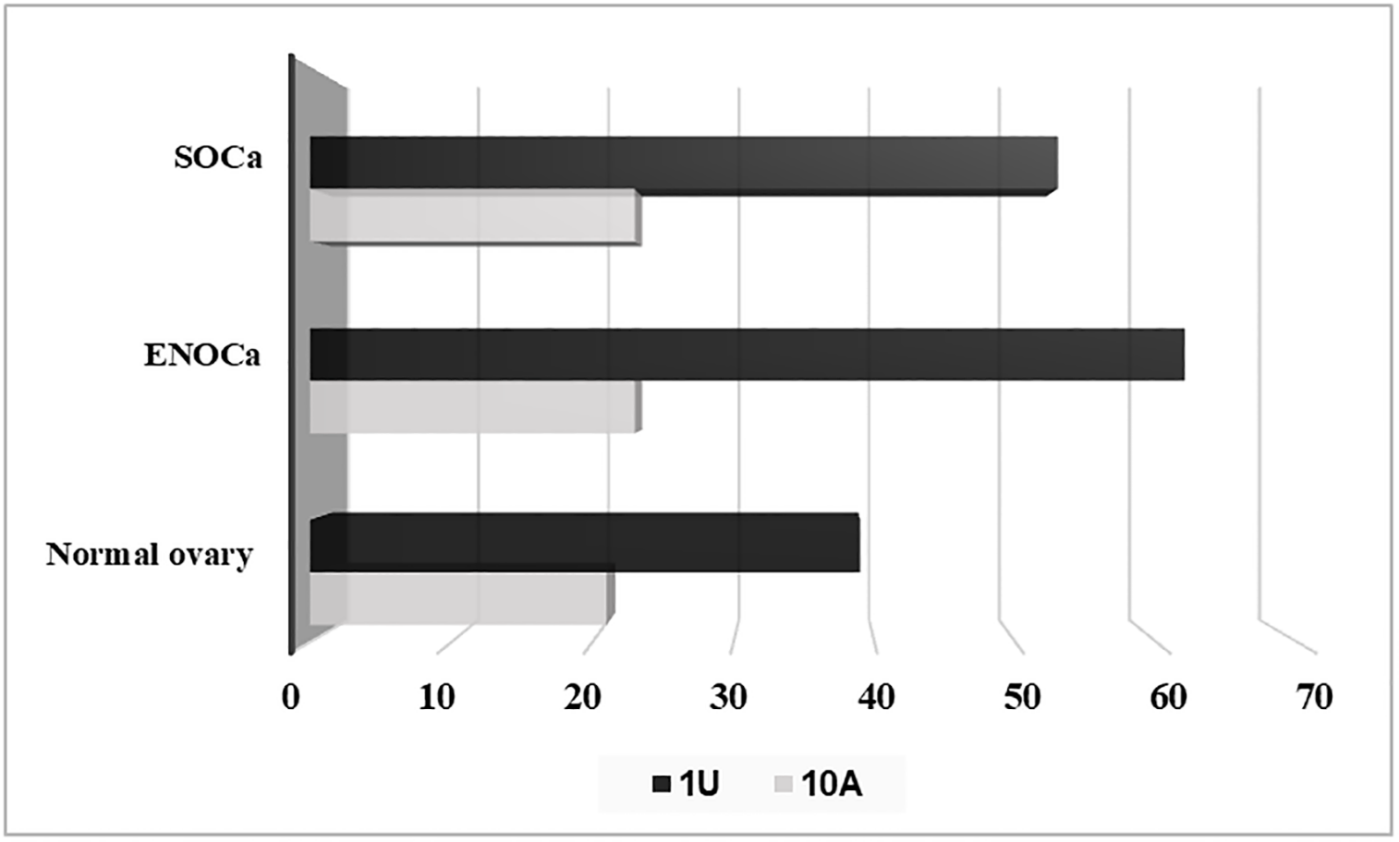
The biogenetic sequence signatures of the intron derived piRNAs (piRtrons). The preference of 1U & 10A are observed among piRtrons in ENOCa, SOCa and normal ovary.

More than 50% of piRNAs are found housed within different regions of PCGs such as exons and introns. 25%, 24% and 23% of the total piRNAs in ENOCa, SOCa, and normal ovary respectively are originated from exons of PCGs which are marginally lesser than the piRtrons (piRNAs originated from intron) which constituted ~1/3^rd^ of total piRNAs in each of the samples (Fig 4B). This indicates that largest fraction of piRNAs is originated from the intronic regions, which was earlier thought as a dark matter of the genome than any other regions, unlike reported elsewhere [36, 37]. We observed the least preference of adenine at 10^th^ position and abundance of uridine at 1^st^ position in these piRNAs suggesting intron derived piRNAs are mostly transcribed by primary biogenesis pathway (Fig 5). The exon-derived piRNAs can target and regulate the expression of any available transcript originating from the opposite strand of the same PCG.

#### ncRNA-derived piRNAs

In this study, we observed a significant proportion of piRNAs are originated from other ncRNAs, which is mainly comprised of tRNAs, lncRNAs, rRNAs, and srpRNAs. tRNAs, the most important class of ncRNAs, has been recently reported to be the hot spot of origin for the HIWI2 associated piRNAs in MDA-MB-231 breast cancer cells [38]. The piRNAs identified from this study also mapped to different tRNA species (S3 Table), among which majority of them mapped to 5′ end of four tRNA species (tRNAVal(CAC), tRNAVal(AAC), tRNAGly(GCC), tRNAMet(CAT)) in ENOCa and SOCa, while piRNAs in normal ovary dominantly mapped to two tRNA species (tRNAMet(CAT), tRNAGly(GCC)). However, none of the piRNAs either from the tumors or normal ovary samples mapped to tRNAs of His, Phe and Trp. Based on these observations, we can speculate that tRNA fragments from 5^/^ half generate piRNAs which probably associate with PIWI proteins to modulate silencing of genes which remain to be fully explicated. We also observed piRNAs are originated from lncRNAs which account for 41%, 37.2% and 41.4% of total ncRNA-derived piRNAs in ENOCa, SOCa and normal ovary which is 14%, 13% and 12% of total piRNAs in these samples respectively. We can also speculate that lncRNAs act as a precursor of piRNAs similar to that of tRNAs in addition to its other putative emerging functions.

#### Repeat and pseudogene-derived piRNAs

Intriguingly, least number of piRNAs showed their origin from the repeats or pseudogenes in comparison with overall genomic ancestry (Fig 4B). The piRNAs were found to be originated from the repeats, mainly from retrotransposons (SINE, LINE, LTR, DNA-Tigger), and composite retrotransposons (SVA) that constitute 13%, 6% and 4% of total piRNAs in normal ovary, ENOCa and SOCa respectively (Fig 4B). We observed only 5% of piRNAs in two EOCa, and 2% in normal ovarian tissue are originated from the pseudogenes. Subsequent analysis revealed that majority of the pseudogene-derived piRNAs in each sample is primarily originated from primary biogenesis pathway. Recently, Haifan Lin and his group at Yale University reported that piRNAs derived from transposons and pseudogenes mediate the degradation of a large fraction of mRNAs and lncRNA transcriptome in mouse late spermatocytes via the piRNA pathway [37]. We conjecture that the subset of piRNAs originated from these genomic contexts in both EOCa, and normal ovary samples might be regulating a significant fraction of lncRNome as well as mRNome with the help of PIWIL1 or two other human PIWILs orthologues in a similar manner as reported by Lin and his group [37].

### piRNA clusters are abundant in ENOCa and enriched with TEs

**Fig 6:**
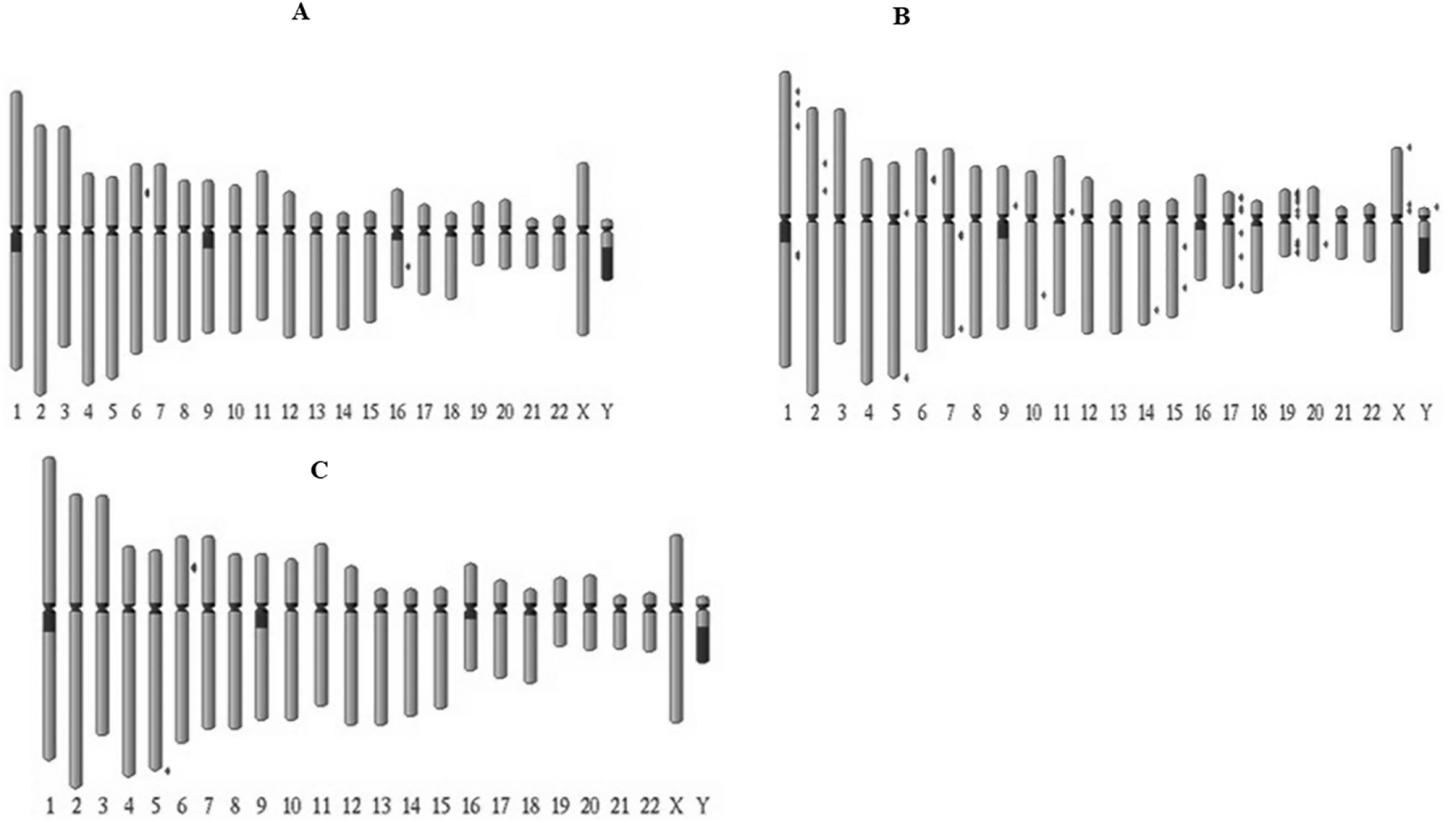
The chromosomal distribution of piRNA clusters. Location of piRNA clusters on human chromosomes in A. Normal ovary, B. ENOCa and C. SOCa samples.

The piRNAs detected in these samples showed a distinctive localization pattern in the human genome supporting their origin from piRNA clusters that are known to genetically control the activity of TEs. We obtained the landscape of piRNA clusters in each sample by searching for a minimum of ten non-redundant piRNAs located on either of the strands on sliding window of 20 kb by 1 kb steps along each chromosome. We found 58 clusters in ENOCa, while only a few clusters were found in SOCa and normal ovary (5) (Fig 6A–6C).

We found that majority of the piRNA clusters in all three samples mostly harbor repeat elements encompassing LTRs, LINEs, etc., among which 12 ENOCa-clusters were exclusively accumulated within the repeat elements. The piRNAs originated from these clusters can be considered as “reincarnated” retrotransposons and might be controlling transposable elements (TEs) from which these have been derived as thought conventionally [38]. Taking into account of the prevalence of TEs within the piRNA clusters, we can propose that these might act as “TE traps” [39] in cancers of the ovary, especially ENOCa. Besides, the piRNA clusters were also found to harbor other genes, such as pseudogenes, PCGs, ncRNAs, etc., but with minor fraction compared to the repeat elements. We found 14 piRNA clusters in ENOCa that contains at least one pseudogene or lncRNA within it. The lncRNAs which are part of piRNA clusters in ENOCa are SNAPIN (NR_052019, NR_052020), NMRK2 (NR_110316), and TRIM41 (NR_045218). Strikingly, we did not find any lncRNAs located within any of the piRNA clusters predicted in SOCa and normal ovary samples. However, we found only PGOHUM00000243083 pseudogene present in both SOCa and normal ovary samples located on same piRNA cluster on chromosome 6. The genomic location corresponding to this cluster in SOCa and normal ovary is seen to be part of a slightly bigger piRNA cluster predicted in ENOCa.

Looking at the chromosomal distribution of clusters in ENOCa, we found chromosome 19 was ahead of chromosomes 17 and 6 in harboring highest number of piRNA clusters within it. Fourteen clusters were predicted within chromosome 19, whereas only seven and six clusters were found on chromosome 17 and 6 respectively. Interestingly, we found the largest piRNA cluster of length 39663 nts in ENOCa residing on chromosome 6 (26526725–26566387 nts) that contain 20 non-redundant piRNAs, among which most of the piRNAs are located on its sense strand.

### piRNAs showed genomic region-specific conservation patterns

**Fig 7:**
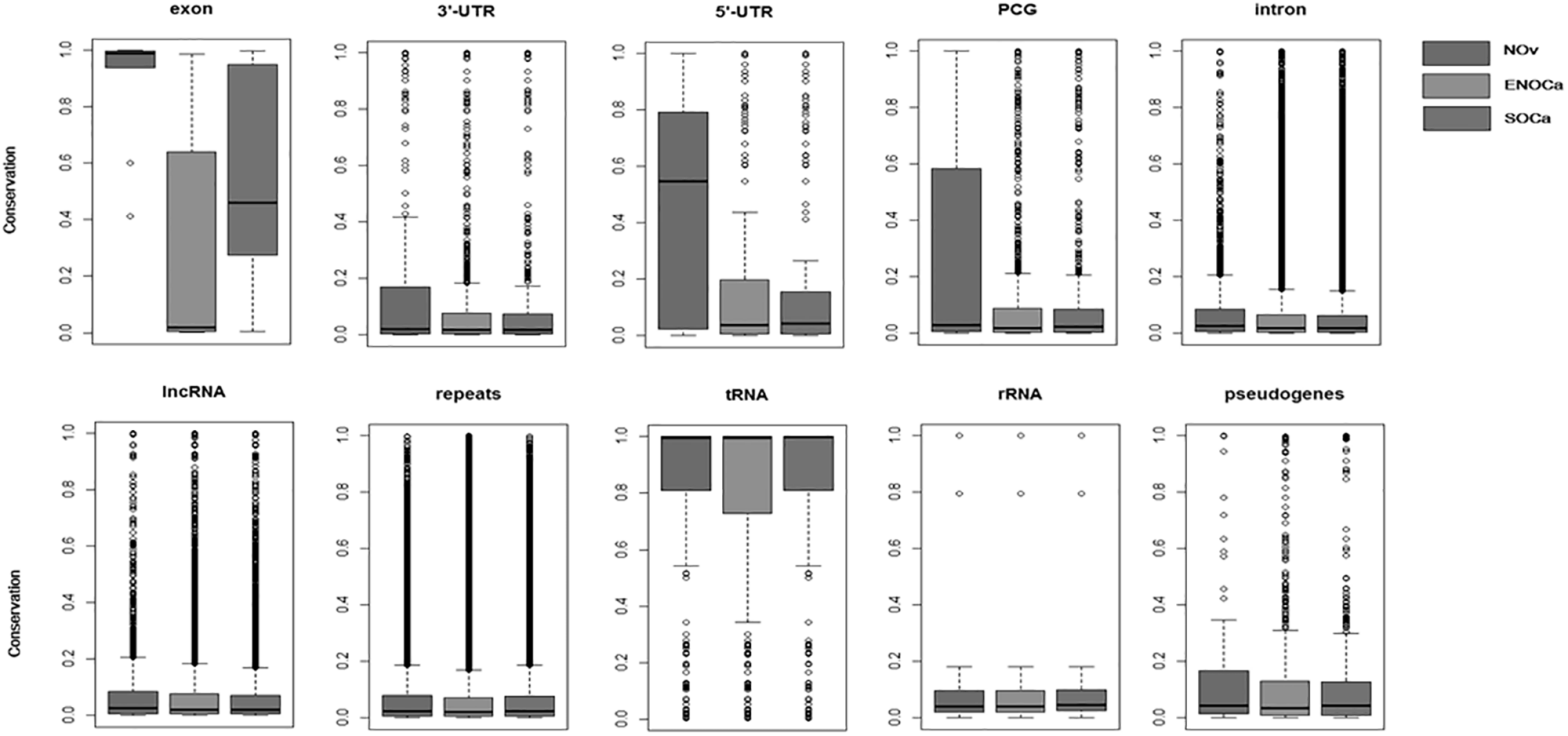
Box plot analysis showing distribution of conservation scores of piRNAs originated from various genomic contexts in three samples (Normal ovary, ENOCa, SOCa). Center lines show the medians; box limits indicate the 25^th^ and 75^th^ percentiles as determined by R program; whiskers extend to minimum and maximum values; crosses represent sample means; bars indicate 90% confidence intervals of the means; width of the boxes is proportional to the square root of the sample size.

Investigation of the conservation patterns of the piRNAs originated from different genomic regions revealed some interesting observations. The conservation pattern is very impressive for piRNAs originated from tRNAs with a median value of phastcons score of 0.994 in all three samples, whereas piRNAs originated from lncRNA, rRNA, repeats, and introns are marginally conserved with median phastcons score lying between 0.02 and 0.03 (Fig 7). Further, piRNAs mapped to CDS of mRNAs are highly conserved in all samples compared to other parts of mRNAs (5^/^UTR, 3^/^UTR). We can conjecture that the genomic origin of piRNAs determine the conservation status of this class of small RNA in these three samples. This can be evaluated in other cells and tissues the way we have reported in healthy human brain and Alzheimer’s disease-affected brain [19] to provide a generalized view of conservation of piRNAs from different genomic locations.

### Differentially expressed piRNAs in ENOCa and SOCa

In this study, piRNAs in each tumor sample showed differential piRNA profiles compared with the normal ovary. We observed 159 differentially expressed (DE) piRNAs in ENOCa and 143 DE piRNAs in SOCa. The DE piRNAs in ENOCa is comprised of 74 up-regulated and 77 down-regulated piRNAs as well as 8 anomalous expressed piRNAs which are excluded from the analysis (S4 Table). In contrast, DE piRNAs of SOCa includes 56 up-regulated and 81 down-regulated piRNAs excluding 6 piRNAs showing anomalous expression as predicted from iMir and mirTools2 (S4 Table). Surprisingly, 55% of DE piRNAs in ENOCa and 27% of DE piRNAs in SOCa were found to originate from intronic regions which hint towards the fact that introns probably contribute to the development of an ovarian epithelial neoplasm.

### Transcriptional profiles of human ENOCa and SOCa

We analyzed microarray data of ENOCa and SOCa of Affymetrix platform (Human Genome U133A technology) from Gene Expression Omnibus database (GSE6008) using GeneSpring GX 12.6 and obtained the transcriptional profile of genes differentially expressed in both EOCa types. We found 652 up-regulated and 745 down-regulated transcripts in ENOCa, whereas 703 up-regulated and 790 down-regulated transcripts in SOCa with fold-change cut-off ≥2.0 and p-value ≤0.05.

### piRNA target genes harboring RTEs in ENOCa and SOCa

piRNAs are known to affect the transposon activity epigenetically and post-transcriptionally by suppressing TEs, which is essential to maintain and protect the genomic stability in cells [17, 40]. Among TEs, RTEs such as Long Interspersed Elements (LINEs) and Short Interspersed Elements (SINEs) are most abundant that comprise ~50% of the mammalian genome [41]. Moreover, silencing of RTEs by piRNAs has been well documented elsewhere [42]. The precise mechanism by which piRNA promote silencing is not clear; however recent studies have shown that piRNAs can form piRISC and induce mRNA repression via imperfect base-pairing between the piRNAs and target mRNAs by a mechanism that closely resembles miRNAs [43] but with extensive sequence complementarity as proposed by Hashim et al. [21] and Goh et al. [31]. We implemented these concepts in our target prediction pipeline (see Materials & methods) to obtain a list of target genes, which are then enriched by pathway analysis to gain more insight into the molecular processes of OCa wherein piRNAs are presumably involved.

To better understand the interplay between piRNA and RTE-containing DE genes in OCa, we considered down-regulated genes of ENOCa (915 transcripts) and SOCa (849 transcripts) that harbor RTEs while predicting targets of 74 and 56 up-regulated piRNAs of ENOCa and SOCa respectively. We found 67 piRNAs are targeting 311 transcripts in ENOCa, whereas 50 piRNAs are targeting 333 transcripts in SOCa. The above predicted sets of targets are used for functional analysis to unveil the neoplastic biological processes regulated by DE piRNAs and their targets in OCa.

### Functional analysis of piRNAs and their targets

To elucidate the biological networks and pathways modulated by piRNA-targeted genes harboring RTEs, we performed functional analysis using MetaCore (Thomson Reuters). From this study, we found 308 and 290 disease functions enriched in ENOCa and SOCa with 160 genes and 172 genes respectively. All of these were related to cancer, apoptosis, cellular movement, cell cycle and cell-cell signaling. We then screened these target genes enriched in ENOCa and SOCa by checking the presence of their upstream regulators reported previously. We obtained 50 genes in ENOCa and 41 genes in SOCa that do not have any upstream regulator reported earlier and hence could be regulated by piRNAs. We then screened the genes whose piRNA binding sites fall within the repeat regions. We obtained 20 genes (30 transcripts targeted by 10 piRNAs) in ENOCa and 21 genes (39 transcripts targeted by 8 piRNAs) in SOCa satisfying this criterion. Further, we exercised two screening criteria, i) piRNAs having 2–21 nt target binding sites allowing upto one wobble pairing or mismatch and ii) targets appearing either in canonical pathways (CPs) or networks to identify DE piRNAs and their targets which possibly have a significant role in tumorigenesis of OCa. We found 10 genes are exclusively targeted by piR-52207 in ENOCa and 7 genes targeted by piR-52207 and piR-33733 in SOCa showing 2–21 nts binding sites. Among these, we found only 4 genes in ENOCa and 3 genes in SOCa are enriched in CPs and networks satisfying the second criteria. Interestingly, all these targets contain SINE elements and harbor target-binding sites of corresponding piRNAs within 3^/^UTR regions (S5 Table).

**Fig 8:**
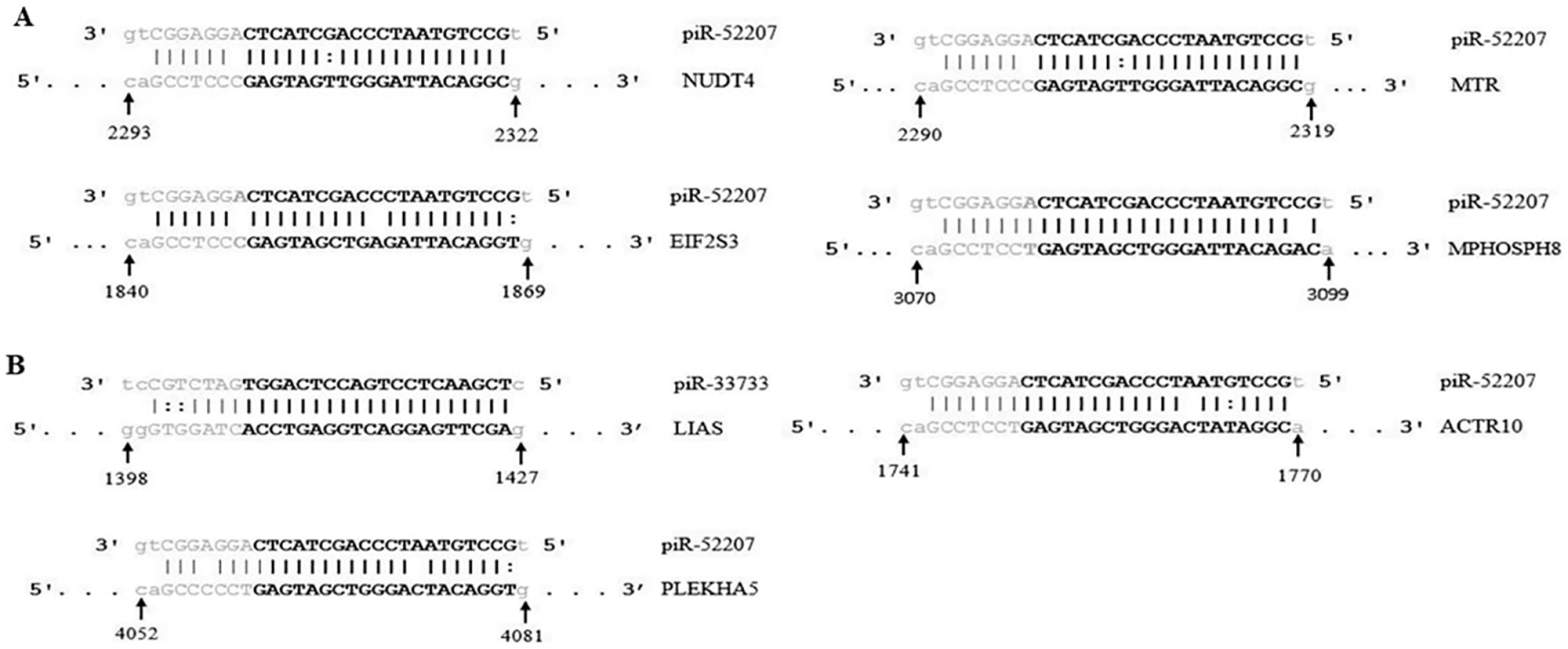
piRNA-target duplexes in ENOCa and SOCa. A. The binding sites of piR-52207 with its targets NUDT4, MTR, EIF2S3 and MPHOSPH8 in ENOCa; B. The binding sites of piR-33733 and piR-52207 with its targets LIAS and ACTR10, PLEKHA5 respectively in SOCa.

**Fig 9:**
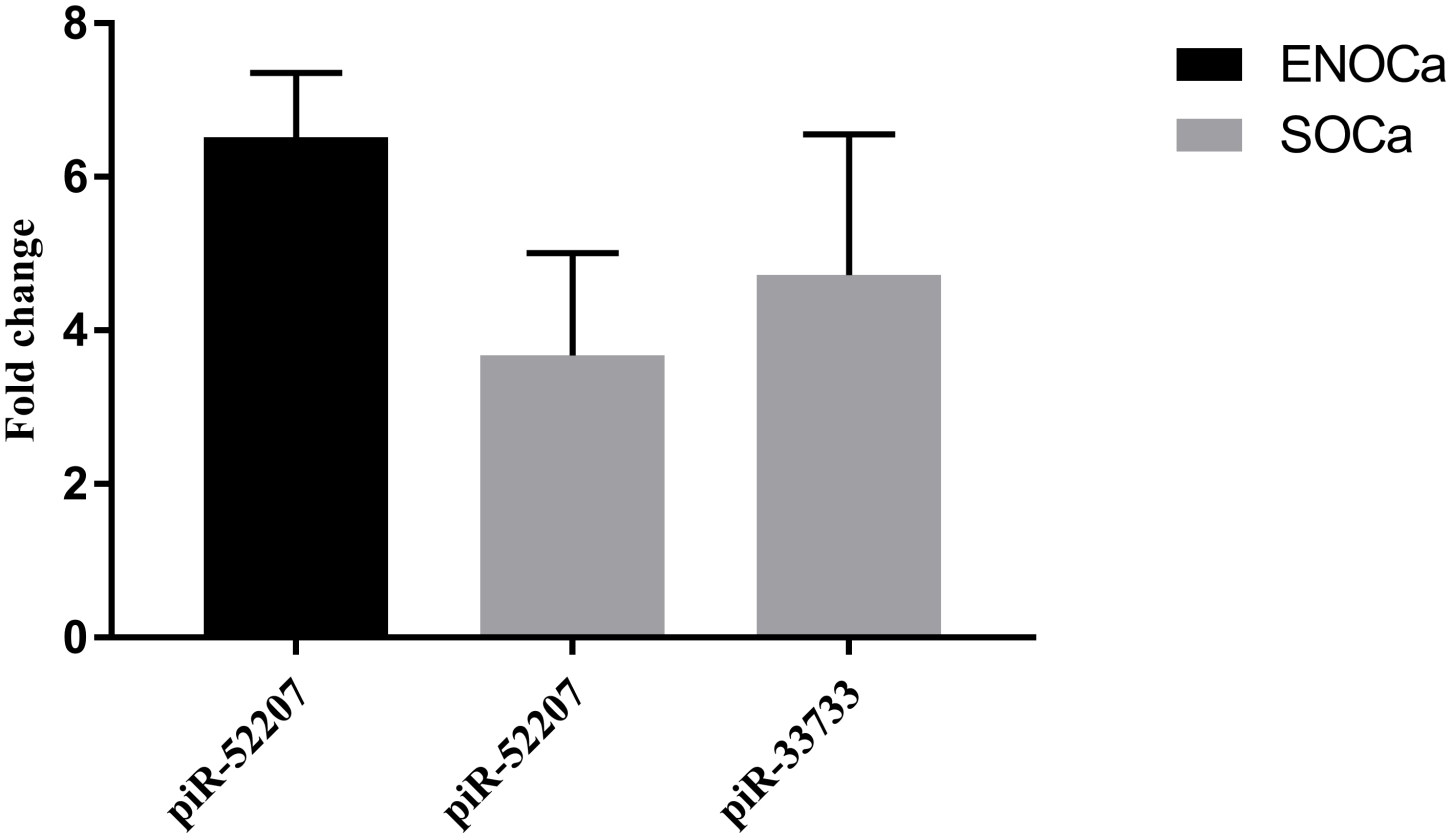
Validation of expression of piRNAs in ovarian cancer subtypes with respect to normal ovarian tissue by qRT-PCR.

**Fig 10:**
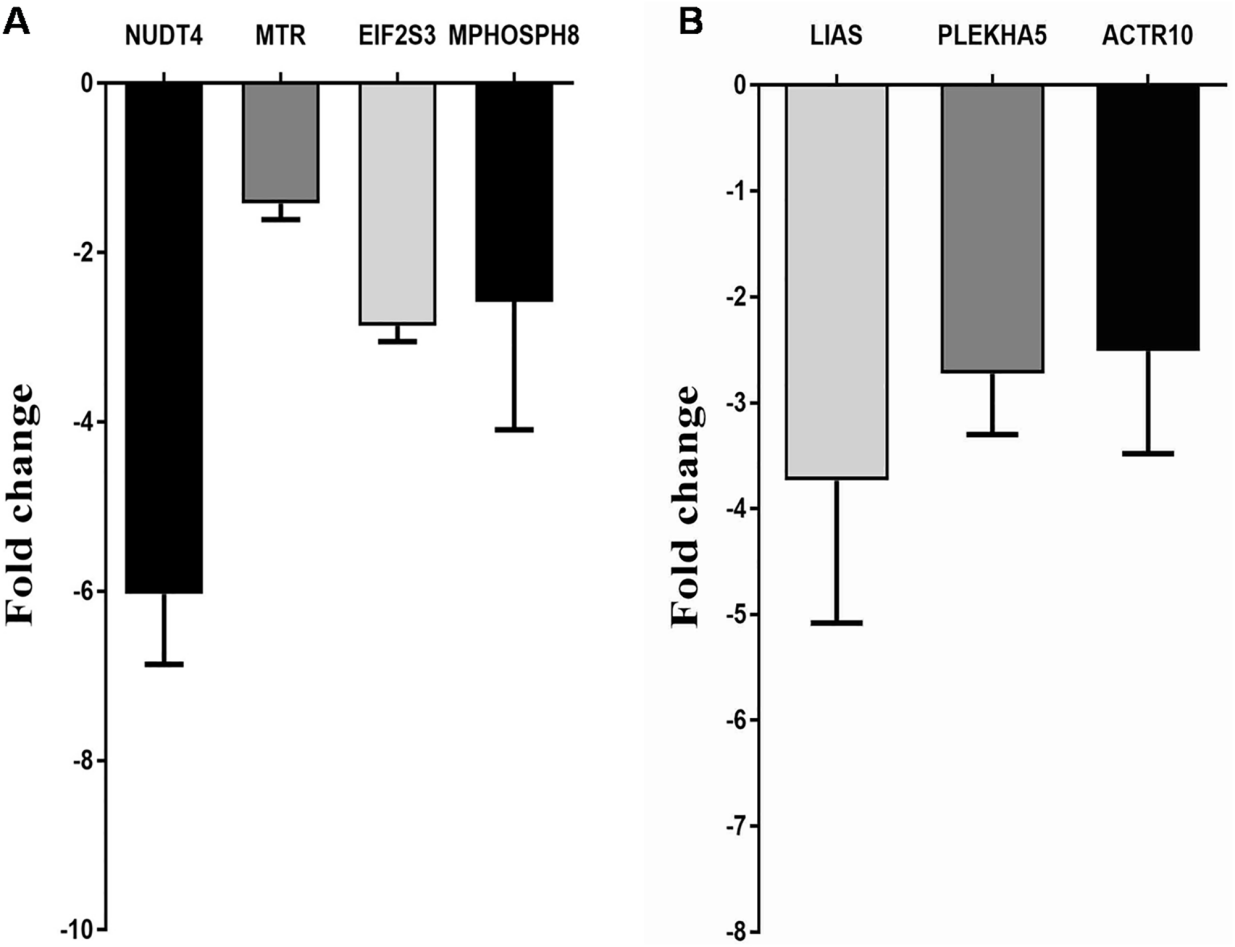
Expression profile of piRNA targets by qRT-PCR in EOCa subtypes. A. The relative expression of genes targeted by piR-52207 in ENOCa; B. The relative expression of genes targeted by piR-33733 and piR-52207) in SOCa.

**Fig 11:**
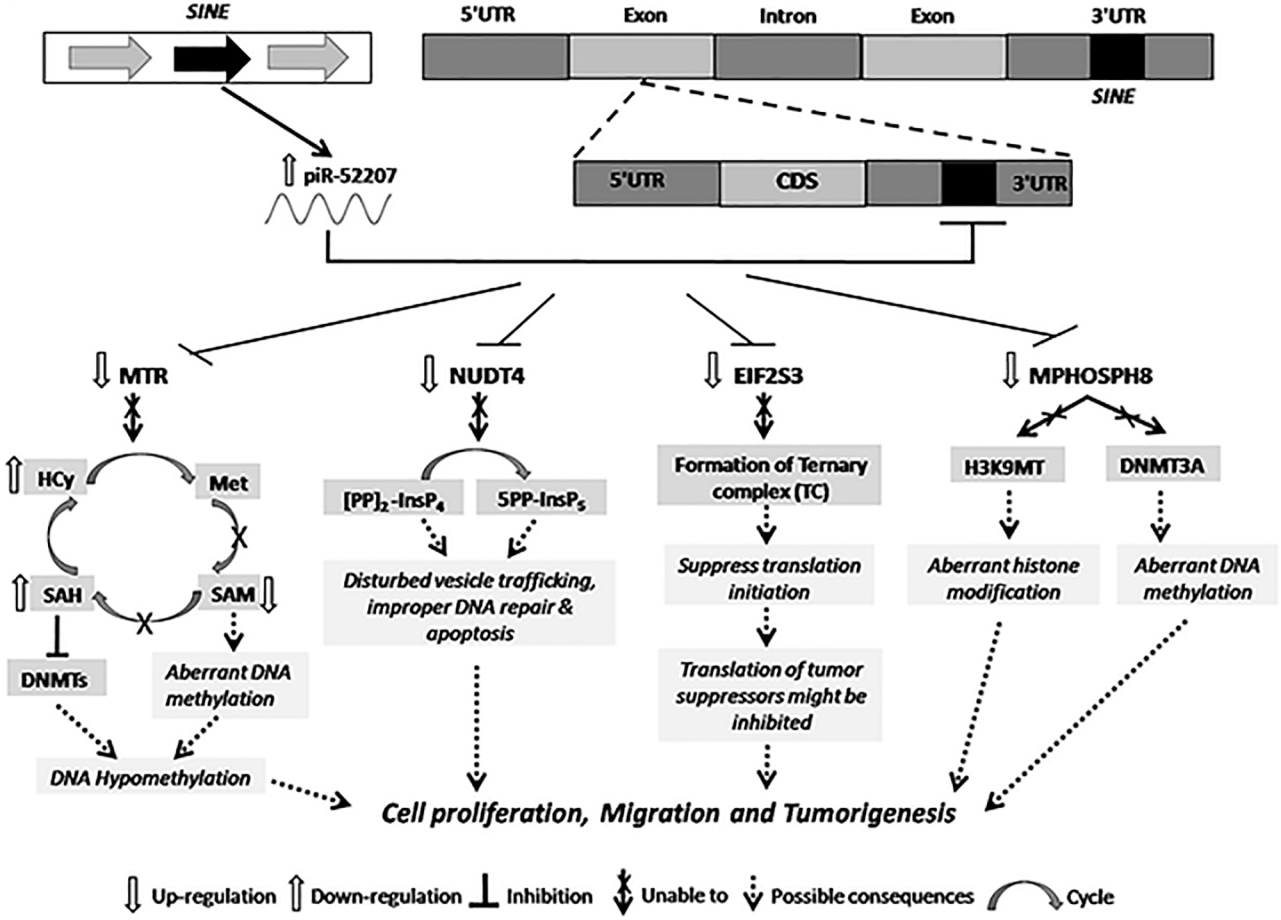
The possible effects of piR-52207 on target genes and subsequent pathophysiological consequences in ENOCa.

The four enriched targets of ENOCa- NUDT4, MTR, EIF2S3 and MPHOSPH8 targeted by piR-52207 are possibly regulating 3-phosphoinositide biosynthesis, D-myo-inositol-5-phosphate, folate transformation I, VEGF signaling and EIF2 signaling in ENOCa (Table 1). We noticed piR-52207 is showing extensive sequence complementarity on these four targets which indicate the probable role of this piRNA in ENOCa by regulating these genes. NUDT4 (nudix hydrolase 4) encodes diphosphoinositol polyphosphate phosphohydrolases 2(DIPP2) that controls the turnover of diphosphoinositol polyphosphates (DIPPs) leading to regulation of intracellular vesicle trafficking and DNA repair [44]. Another member of the same family, NUDT3 codes for DIPP1 having 76% identity with DIPP2 with the similar catalytic site and substrate specificity for DIPPs such as 5PP-InsP_5_ (diphosphoinositolpentakisphosphate) and PPInsP_4_ (diphosphoinositoltetrakisphosphate) [44, 45]. This has been reported as decapping enzyme, and its low level induces cell migration in breast cancer cells by modulating a subset of mRNAs [46]. Based on these evidences and seeing the extensive sequence complementarity of piR-52207 of 2–28 nts to NUDT4 with one G:U bp at 15^th^ position and one mismatch at 22^nd^ position (Fig 8A), we conjecture that down-regulation of NUDT4 in ENOCa is due to efficient targeting by piR-52207 which induces tumour growth and cell migration in ENOCa. This was further strengthened by validating the expression of piR-52207 (Fig 9) and NUDT4 (Fig 10A) by qRT-PCR study. MTR (5-methyltetrahydrofolate-homocysteine methyl transferase), also known as methionine synthase is an important enzyme of one-carbon metabolism, which is known to remethylate homocysteine (HCy) to methionine (Met). Lower expression of MTR has been reported to elevate the level of cellular Hcy and S-adenosyl Homocysteine (SAH) level, which inhibits DNA methyltransferase activity (DNMTs) [47] thereby leading to DNA hypomethylation [48] and increase in tumorigenic potential in prostate cancer [49, 50]. We observed a complete watson-crick complementary base pairing of 2–21 nts of piR-52207 with MTR (Fig 8A). In addition, qRT-PCR study showed that piR-52207 (Fig 9) is up-regulated about 6 folds while MTR is downregulated about 1.5 fold in ENOCa (Fig 10A). This indicates that targeting by piR-52207 might have lead to downregulation of MTR gene, which might be responsible for the enhanced tumorigenic properties in ENOCa. Another gene, EIF2S3 (eukaryotic translation initiation factor 2, subunit 3 gamma) is a crucial member of eukaryotic initiation factors (EIFs) involved in VEGF signaling which is also targeted by piR-52207. Further, EIFs are essential factors in the early steps of protein synthesis [51] and their aberrant expression have been reported causing uncontrolled cell proliferation associated with tumour development [51]. We assume that piR-52207 is impacting down-regulation of EIF2S3 of about 3-fold (refer to Fig 10A) in ENOCa. Similarly, MPHOSPH8 (M-phase phosphoprotein 8) is found to be a potential target of piR-52207. It promotes carcinogenesis, often interacting with H3K9methyl transferases leading to improper histone modification and DNMT3A leading aberrant DNA methylation. This facilitates E-cadherin repression which in turn leads to epithelial to mesenchymal transition (EMT) thereby aiding in tumour cell proliferation [52–54]. We speculate that down-regulation of MPHOSPH8 in ENOCa (Fig 10A) might be promoting EMT which is probably regulated by piR-52207 (Fig 8A). The reciprocal expression of piR-52207 (Fig 9) and its four targets observed from qRT-PCR study provided additional evidences that the said piRNA might be targeting these genes and controling key processes/pathways leading to tumorigenesis of ENOCa. The possible tumorigenic consequences of targeting by piR-52207 in ENOCa is portrayed in Fig 11.

**Table 1:** Canonical pathway(s) and network(s) enriched in ENOCa.

Sl.No	Target genes	piRNAs	Enriched CP(s)	Enriched Network (s)
1	MTR	**piR-52207** piR-35982 piR-31080 piR-51309 piR-36170 piR-30924	Methionine Salvage II (Mammalian) Superpathway of Methionine Degradation Folate Transformations I	Metabolic Disease, Nutritional Disease, Cardiovascular Disease
2	NUDT4	**piR-52207** piR-35548	3-phosphoinositide Biosynthesis Superpathway of Inositol Phosphate Compounds D-myo-inositol (1,4,5,6) Tetrakisphosphate Biosynthesis D-myo-inositol (3,4,5,6)-tetrakisphosphate Biosynthesis 3-phosphoinositide Degradation D-myo-inositol-5-phosphate Metabolism	Cell Cycle, Gene Expression, Cardiovascular System Development and Function
3	EIF2S3	**piR-52207** piR-33486	VEGF Signaling Regulation of eIF4 and p70S6K Signaling EIF2 Signaling	Cell Cycle, Hematological Disease, Immunological Disease
4	MPHOSPH8	**piR-52207**	Not enriched in CP(s)	Cancer, Dermatological Diseases and Conditions, Organismal Injury and Abnormalities

The piRNAs represented in **bold** show watson-crick base pairing of target genes to 2–21 nts of piRNAs. The remaining piRNAs show binding to target genes with alignment score (sc ≥170) and energy (en ≤ -20Kcal/mol) but not satisfying above criteria.

In SOCa, we found Lipoate biosynthesis pathway is predicted to be modulated through piR-33733 via targeting LIAS (lipoic acid synthetase) gene. LIAS gene is involved in the synthesis of Lipoic Acid (LA), a well-known antioxidant that induces apoptosis by downregulating the anti-apoptotic genes (Mcl-1 and Bcl-x_L_) and upregulating a proapoptotic gene, Bim in OCa [55, 56]. We found 2–25 nts of piR-33733 which is upregulated in SOCa is bound to LIAS which is downregulated in the same cancer with watson-crick base-pairing (Figs 9 and 10B). This tempted us to believe that lower expression of LIAS is modulating tumorigenesis of SOCa due to target repression by piR-33733 (Fig 8B).

**Fig 12:**
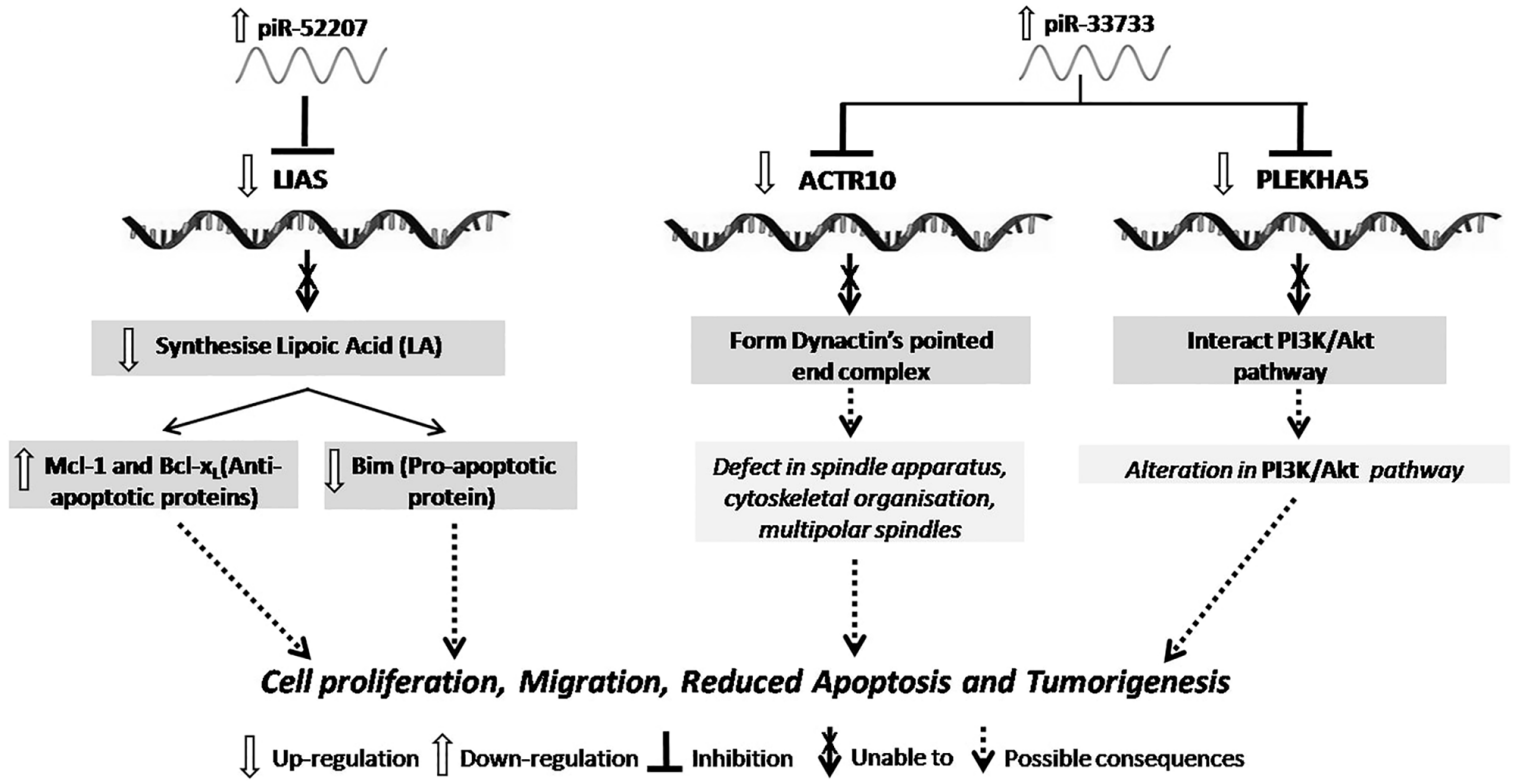
The possible effects of piR-33733 and piR-52207 on target genes and subsequent pathophysiological consequences in SOCa.

Besides, we observed two genes (ACTR10, PLEKHA5) that are enriched in cancer-related networks are probably regulated by piR-52207 in SOCa (Table 2). PLEKHA5 (Pleckstrin homology domain containing family A member 5) binds to phosphoinositol and control various cellular events such as cellular signalling, phosphoinositide metabolism (PI3K/Akt pathway) [57], and cytoskeletal rearrangement [58]. Moreover, its expression is reported in cell membranes and microtubules suggesting its possible role in cell migration and cell-cell signalling [59]. Thus, downregulation of PLEKHA5 in SOCa as evident from microarray analysis and confirmed by our qRT-PCR study might be due to efficient targeting by piR-52207 (Fig 8B). ACTR10 (actin-related protein 10 homolog, also known as Arp11) has been identified as a component of dynein-associated complex, dynactin, that assist cytoplasmic dynein by regulating its cargo binding ability [60, 61]. Arp11 gene is reported to act as a tumour suppressor in nude mice as its overexpression suppresses tumorigenesis by regulating the transcription of multiple genes involved in cytoskeletal organization and cell adhesion necessary for suppressing the tumorigenic potential. This tumor suppressor might have been inactive or suppressed in SOCa by piR-52207 resulting in failure of transcriptional regulation of cytoskeletal and cell adhesion genes leading to cancer progression. Refer to Fig 8B for details of binding sites between piR-52207 and ACTR10. The reciprocal expression of piR-52207 (Fig 9) and PLEKHA5, ACTR10 (Fig 10B) obtained from our qRT-PCR study further confirms that piR-52207 is possibly modulating above processes in SOCa through targeting of these genes. The functional consequences of piR-33733 and piR-52207 in SOCa tempted us to believe that both of them have major roles in neoplastic regulations (Fig 12).

**Table 2:** Canonical pathway(s) and network(s) enriched in SOCa.

Sl.No	Target genes	piRNAs	Enriched CP(s)	Enriched Network (s)
1	LIAS	**piR-33733**	Lipoate Biosynthesis and Incorporation II	Connective Tissue Disorders, Developmental Disorder, Hereditary Disorder
2	ACTR10	**piR-52207** piR-61135	Not enriched in CP(s)	Cancer, Cardiovascular Disease, Organismal Injury and Abnormalities
3	PLEKHA5	**piR-52207** piR-43770 piR-33783 piR-52729 piR-33733 piR-43773	Not enriched in CP(s)	Cancer, Cardiovascular Disease, Organismal Injury and Abnormalities

The piRNAs represented in **bold** show watson-crick base pairing of target genes to 2–21 nts of piRNAs. The remaining piRNAs show binding to target genes with alignment score (sc ≥170) and energy (en ≤ -20Kcal/mol) but not satisfying above criteria.

In addition, we found 62 transcripts (66 binding sites) solely bound by piR-52207 in ENOCa while searching for continuous base paring of 2–21 nts of DE piRNAs allowing up to one wobble pairing or mismatch within target binding regions and ignoring target enrichment in cancer-related functions, processes or pathways. While observing these binding sites, we noticed that 63 out of 66 binding sites of piR-52207 are falling within 3’UTRs. Moreover, this 30 nt long, abundant piRNA with GC content of 56.67% showing 1U10A bias is derived from numerous genomic contexts, mostly from Alu TEs on multiple chromosomes of a human. In SOCa, we found two piRNAs, piR-33733 and piR-52207 targeting 81 transcripts containing 90 binding sites satisfying above criteria. Upon inspection, we discovered that piR-33733 targets 25 transcripts whose binding sites reside within 3’UTRs only, while piR-52207 harbors 62 sites in 3’UTRs and 3 sites at 5’UTRs. These indicate that piR-52207 and piR-33733 possibly have significant roles in the neoplastic events of ENOCa and SOCa by modulating the expression of several targets with the extensive complementary base paring. The functional relevance of piR-52207 and piR-33733 in EOCa was further strengthened by seeing their involvement in several GOs and key pathways enriched in both ENOCa and SOCa.

## Conclusions

The presence and functioning of piRNAs in germline and gonads have been extensively studied revealing their diversified defensive function as TE-traps in association with the piwi gene, but little is known about their role in somatic cells. Recent studies have reported the role of piRNA-Piwi complex in tumor prognosis including breast, bladder, and gastric cancers [21, 22, 62]. We realized that these findings are just the tip of the iceberg because the relationships between the components of piRNA pathway and tumor cell biology in ovarian cancer progression have not yet been studied. Therefore, in this study, we investigated in detail the behavior of piRNAs, in human ovary, ENOCa, and SOCa samples. We report here the expression of three human PIWIL genes and proteins except for PIWIL3, the known components of the biogenesis and effector pathways in these three samples by qRT-PCR and WB respectively. In addition to this, we reported a specific set of piRNAs is expressed in both the types of human EOCa and normal ovary by high-throughput sequencing of small RNAs, indicating that PIWI-piRNA pathway is active in the ovary as well as cancer tissues of this origin.

We observed 84% of piRNAs identified in the normal ovary, ENOCa and SOCa are primary piRNAs demonstrating the conserved nature of the biogenesis mechanism operational in these samples. While checking conservation status of piRNAs originated from different genomic contexts in the human genome, we observed the varied level of conservations in various locations. The piRNAs originated from tRNAs were highly conserved with a median phastcons score of 0.994 in all three samples, whereas piRNAs originated from other ncRNAs such as lncRNAs and rRNAs are poorly conserved in addition to repeats and introns (Fig 6). This conservation pattern of piRNAs of EOCa can be either organism-specific or taxon-specific which can further be confirmed by checking conservation status of piRNAs in other organisms and cells/tissues. In our analysis, we also found that many of the piRNAs are derived from tRNA in each sample which suggests that piRNA pathway could be involved in the regulation of protein translation in ovarian tissues. Our investigation reported 111 piRNAs differentially expressed in both types of malignancies of ovarian epithelial cells which reflect that these two malignancies possibly share common oncogenic processes regulated by piRNAs which were corroborated from the prediction of same pathways enriched by targets of the piRNAs. The exclusive set of significantly over-expressed and under-expressed piRNAs detected in ENOCa and SOCa could provide the knowledgebase for clinical benefits of these ovarian malignancies utilizing these piRNAs as a potential biomarker for early prognosis or as an agent for RNA therapeutics. Apart from known piRNAs, we also identified a large number of novel piRNA-likes molecules in each sample which likely to have some unknown promising role in EOCa types. We can speculate that EOCa expresses a specific set of these molecules that is not yet discovered, and for this reason, escapes the current methods for RNA-Seq data analysis which are based on alignment with already reported piRNAs. Moreover, some of the piRNA-likes predicted using predefined characteristics of piRNAs implemented within the piRNA prediction tools could be plausibly another class of sncRNAs which yet to be characterized.

The inverse correlation between the expression of piRNAs (piR-52207 and piR-33733) and their target genes in ENOCa and SOCa obtained from our qRT-PCR study along with extensive target binding sequence complementarity between them indicated that these genes are possibly regulated by the corresponding piRNAs playing roles in tumorigenesis of epithelial ovarian cancers. However, this could not be validated in multiple samples to draw a generalized inference due to non-availability of cancer tissue samples which is a limitation of our study.

In conclusion, we have found that human ovary and it’s malignancies contain piRNAs, which are most likely participating in a multitude of functions by modulating post-transcriptional regulation of genes involved in oncogenesis of EOCa. Our data set and results described here unveiled a new regulatory RNA landscape involving piRNAs that would aid in the improved understanding of new layer of gene regulations in malignancies of the ovary. Further functional studies are essential to unveil and validate the unknown roles of deregulated piRNAs in oncogenesis and prognosis of EOCa which will add icing on the cake to harness the cancer piRNAs for possible therapeutics of this class of malignancy.

## Supporting information

S1 Table Annotated piRNAs identified in (A) Normal ovary, (B) ENOCa and (C) SOCa. (DOCX)

S2 Table Number of unique reads and read count of miRNAs detected in (A) Normal ovary, (B) ENOCa and (C) SOCa. (DOCX)

S3 Table Abundance of t-RNA derived piRNA in each sample. (DOCX)

S4 Table Differentially expressed (A) up-regulated piRNAs ENOCa, (B) down-regulated piRNAs in ENOCa, (C) up-regulated piRNAs in SOCa, and (D) down-regulated piRNAs in SOCa. (DOCX)

S5 Table List of target genes harboring SINE elements which is targeted by differentially expressed up-regulated piR-52207 in (A) ENOCa and (B) SOCa. (DOCX)
