# Supplementary material for: Genome-wide profiling of the PIWI-interacting RNA-mRNA regulatory networks in epithelial ovarian cancers
Source: PLoS One. 2018 Jan 10;13(1):e0190485. doi: 10.1371/journal.pone.0190485 (PMC5761873; doi:10.1371/journal.pone.0190485)
Supplement: S1 Table — (DOCX) [file pone.0190485.s001.docx]

Table S1A. Annotated piRNAs identified in Normal ovary

| **S. No** | **piRNA** | **NCBI Accession** | **piRNA sequence** | **Length** |
| --- | --- | --- | --- | --- |
| 1 | piR-34374 | DQ596308 | GACCAATGATGAGACAGTGTTTATGAAA | 28 |
| 2 | piR-33437 | DQ593325 | CGGAAGCGTGCTGGGCCCATAACCCAGA | 28 |
| 3 | piR-34372 | DQ596306 | GACCAATGATGACCACTGGTGGCGTTTGAGT | 31 |
| 4 | piR-33686 | DQ593574 | CTCAGTGATGCAATCTCTGTGTGGTTCTGAGA | 32 |
| 5 | piR-33536 | DQ593424 | CGTGAGTTCGATCCTCACACGGGGCACCA | 29 |
| 6 | piR-44312 | DQ576200 | TCCTCATTAGTATAGTGGTGAGTATCCC | 28 |
| 7 | piR-43768 | DQ575656 | TCCCTGGTAGTCTAGTGGTTAGGATTC | 27 |
| 8 | piR-34375 | DQ596309 | GACCAATGATGAGACAGTGTTTATGAAC | 28 |
| 9 | piR-34984 | DQ596918 | GATGTCTGTGTGGAAAGCGGCTGTGCA | 27 |
| 10 | piR-50385 | DQ583273 | TGCAAGTACATCTACGCCAAGGACCGC | 27 |
| 11 | piR-45371 | DQ577259 | TCTACTGAACTGCCATGAGGAAACTG | 26 |
| 12 | piR-31612 | DQ571500 | AGTTCGTGATGGATTTGCTTTTTTCTGATT | 30 |
| 13 | piR-34342 | DQ596276 | GACATCCCGATGGTGCAGCCGCTATTAAA | 29 |
| 14 | piR-36173 | DQ598107 | GGGAGGCCCGGGTTCGTTTCCCGGCCAATGCA | 32 |
| 15 | piR-36170 | DQ598104 | GGGAGATGAAGAGGACAGTGACTGAGAGAC | 30 |
| 16 | piR-33470 | DQ593358 | CGGCTGTTAACCGAAAGGTTGGTGGT | 26 |
| 17 | piR-36074 | DQ598008 | GGCTCGTTGGTCTAGGGGTATGATTCTCGG | 30 |
| 18 | piR-61648 | DQ595536 | TTGGTGGTTCAGTGGTAGAATTCTCGCCTGCC | 32 |
| 19 | piR-35406 | DQ597340 | GCCCCTGTGATGAGTTGCCATGCTAATACGGA | 32 |
| 20 | piR-34871 | DQ596805 | GAGTAGAGTGCTTAGTTGAACAGGGCC | 27 |
| 21 | piR-61646 | DQ595534 | TTGGTGGTTCAGTGGTAGAATTCTCGCCTG | 30 |
| 22 | piR-36499 | DQ598433 | GTCCGGTGCGGAGTGCCCTTCGTCCTGGGAA | 31 |
| 23 | piR-34653 | DQ596587 | GAGCACTGTTCGTAACCCGTTAGCCT | 26 |
| 24 | piR-61135 | DQ595023 | TTGCAAGCAACACTCTGTGGCAGATGATC | 29 |
| 25 | piR-35281 | DQ597215 | GCATTGGTGGTATAGTGGTAAGCATAGC | 28 |
| 26 | piR-35284 | DQ597218 | GCATTGGTGGTTCAGTGGTAGAATTCTCAC | 30 |
| 27 | piR-36707 | DQ598641 | GTTAAGATGGCAGAGCCCGGTAATCGCATAA | 31 |
| 28 | piR-36706 | DQ598640 | GTTAACCGAAAGGTTGGTGGTTCGTGCCCA | 30 |
| 29 | piR-33526 | DQ593414 | CGTCCATGATGTTCCGCAACTACCTAC | 27 |
| 30 | piR-33527 | DQ593415 | CGTCCATGATGTTCCGCAACTACCTACA | 28 |
| 31 | piR-34380 | DQ596314 | GACCAATGATGAGTATTCTGGGGTGTCTGAA | 31 |
| 32 | piR-52729 | DQ585617 | TGCTATCTGAGAGATGGTGATGACATTA | 28 |
| 33 | piR-30438 | DQ570326 | ACAGCAGTTGAACATGGGTCAGTCGGTCC | 29 |
| 34 | piR-36318 | DQ598252 | GGTAGTGTGGCCGAGCGGTCTAAGGC | 26 |
| 35 | piR-55213 | DQ588101 | TGGCTATGATCTGCCTTGTTCAAGCTGAGA | 30 |
| 36 | piR-33856 | DQ593744 | CTGCAGTGATGACTTTCTTAGGACACCTTTG | 31 |
| 37 | piR-31038 | DQ570926 | AGCAGGACGGTGGCCATGGAAGTCGGAATCC | 31 |
| 38 | piR-33486 | DQ593374 | CGGGAGGCCCGGGTTCGGTTCCCGGCCAATGC | 32 |
| 39 | piR-51309 | DQ584197 | TGCCAATGATGGTTAAGAATTTCTTCACCTGA | 32 |
| 40 | piR-44717 | DQ576605 | TCCTTAGGTCGCTGGTTCGATTCCGGCTCGAA | 32 |
| 41 | piR-54626 | DQ587514 | TGGCAATGATGACCCACTTGCCCTCACTGA | 30 |
| 42 | piR-43176 | DQ575064 | TCCCAATGATGAGTTGCCATGCTAATACTGA | 31 |
| 43 | piR-31935 | DQ571823 | ATTTGGTGTATGTGCTTGGCTGAGGAGCCAA | 31 |
| 44 | piR-33043 | DQ592931 | CCCCCCACTGCTAAATTTGACTGGCTA | 27 |
| 45 | piR-36063 | DQ597997 | GGCGGGAGTAACTATGACTCTCTTAAGGTA | 30 |
| 46 | piR-31623 | DQ571511 | ATAACTGACGAAGACTACTCCTGTCTGATT | 30 |
| 47 | piR-51657 | DQ584545 | TGCCATGGTAATCCTGCTCAGTACGAGA | 28 |
| 48 | piR-43604 | DQ575492 | TCCCGGCTAGCTCAGTCGGTAGAGCATGA | 29 |
| 49 | piR-52207 | DQ585095 | TGCCTGTAATCCCAGCTACTCAGGAGGCTG | 30 |
| 50 | piR-36743 | DQ598677 | GTTTCCGTAGTGTAGTGGTCATCACGTTCGCC | 32 |
| 51 | piR-60565 | DQ594453 | TTCCCTGGTGGTCTAGTGGTTAGGATTCGGC | 31 |
| 52 | piR-36712 | DQ598646 | GTTCACTGATGAGAGCATTGTTCTGAGCCA | 30 |
| 53 | piR-36241 | DQ598175 | GGGGGATTAGCTCAAATGGTAGAGCGCTCG | 30 |
| 54 | piR-44718 | DQ576606 | TCCTTAGGTCGCTGGTTCGGTTCCGGCTCGA | 31 |
| 55 | piR-36082 | DQ598016 | GGCTCTGTTGCGCAATGGATAGCGCAT | 27 |
| 56 | piR-36717 | DQ598651 | GTTCATGATGACACAGGACCTTGTCTGAAC | 30 |
| 57 | piR-31080 | DQ570968 | AGCCAATGGGGCGAAGCTACCATCTGT | 27 |
| 58 | piR-30229 | DQ570117 | AAGCCAGGGATTGTGGGTTCGGGTCCCATCT | 31 |
| 59 | piR-33519 | DQ593407 | CGTAGTGTAGTGGTCATCACGTTCGCCT | 28 |
| 60 | piR-49900 | DQ581788 | TGATGCCTAAGAAGAACCGGATTGCC | 26 |
| 61 | piR-33748 | DQ593636 | CTCTACTGAACTGCCATGAGGAAACTGCC | 29 |
| 62 | piR-31985 | DQ571873 | CAACAAGTACCGTAAGGGAAAGTTGA | 26 |
| 63 | piR-31531 | DQ571419 | AGTAAGTGAAGATAAAGTGTGTCTGAGG | 28 |
| 64 | piR-31143 | DQ571031 | AGCGTTGGTGGTATAGTGGTGAGCATAGCTGC | 32 |
| 65 | piR-35058 | DQ596992 | GCAATAACAGGTCTGTGATGCCCTTAGA | 28 |
| 66 | piR-35059 | DQ596993 | GCAATCACTGATGTCTCCATGTCTCTGAGCA | 31 |
| 67 | piR-30840 | DQ570728 | AGAACGTGTGGAAAACTAATGACTGAGC | 28 |
| 68 | piR-58469 | DQ591357 | TGTGAATCTGACAACAGAGGCTTACGACCCC | 31 |
| 69 | piR-35982 | DQ597916 | GGCATTGGTGGTTCAGTGGTAGAATTCTCGC | 31 |
| 70 | piR-52206 | DQ585094 | TGCCTGTAATCCCAGCACTTTGGGAGGCCG | 30 |
| 71 | piR-31636 | DQ571524 | ATAGGGTTTACGACCTCGATGTTGGATC | 28 |
| 72 | piR-31638 | DQ571526 | ATAGGTTTGGTCCTAGCCTTTCTATTAGCTCT | 32 |
| 73 | piR-30924 | DQ570812 | AGAGAGGGGCCCGTGCCTTGGAAAGCGTC | 29 |
| 74 | piR-41004 | DQ572892 | TCACAATGCTGACACTCAAACTGCTGACA | 29 |
| 75 | piR-33226 | DQ593114 | CCTCGAACTCCTGACCTCAGGTGATCCACC | 30 |
| 76 | piR-36494 | DQ598428 | GTCCATGATGATTTCAAGTTATCCCTGTCTGA | 32 |
| 77 | piR-43770 | DQ575658 | TCCCTGGTGGTCTAGTGGTTAGGATA | 26 |
| 78 | piR-57125 | DQ590013 | TGGTCGTGGTTGTAGTCCGTGCGAGAA | 27 |
| 79 | piR-43772 | DQ575660 | TCCCTGGTGGTCTAGTGGTTAGGATTCGGCAC | 32 |
| 80 | piR-43773 | DQ575661 | TCCCTGGTTCGATCCCGGGTTTCGGCACC | 29 |
| 81 | piR-51810 | DQ584698 | TGCCCCCATGTCTAACAACATGGCTTTCTCA | 31 |
| 82 | piR-60577 | DQ594465 | TTCCGTAGTGTAGTGGTTATCACGTTCGCCTC | 32 |
| 83 | piR-60576 | DQ594464 | TTCCGTAGTGTAGTGGTTATCACGTTCGCC | 30 |
| 84 | piR-34597 | DQ596531 | GAGAGAGGGGCCCGTGCCTTGGAAAGTG | 28 |
| 85 | piR-36233 | DQ598167 | GGGGCGAAGCTACCATCTGTGGGATT | 26 |
| 86 | piR-30799 | DQ570687 | ACTGTGTGCTGATTGTCACGTTCTGATT | 28 |
| 87 | piR-31970 | DQ571858 | CAAAGTGATTGGTACCTCGTTGTCTGATG | 29 |
| 88 | piR-34456 | DQ596390 | GACGGTGAATACAGGTCTGGAAGTCTGAGGT | 31 |
| 89 | piR-34669 | DQ596603 | GAGCATGGTAATGGATTTATGGTGGGTCCTT | 31 |
| 90 | piR-30451 | DQ570339 | ACAGGTCTGTGATGCCCTTAGATGTCCGG | 29 |
| 91 | piR-33065 | DQ592953 | CCCCTGGTGGTCTAGTGGTTAGGATTCGGC | 30 |
| 92 | piR-33468 | DQ593356 | CGGCTAGCTCAGTCGGTAGAGCATGAGACT | 30 |
| 93 | piR-30625 | DQ570513 | ACCGTCGTAGTTCCGACCATAAACGATGCC | 30 |
| 94 | piR-34929 | DQ596863 | GATCGATGATGACTACCGGTGGCGTATGAGT | 31 |
| 95 | piR-57947 | DQ590835 | TGTAGTGCGCTATGCCGATCGGGTGTCC | 28 |
| 96 | piR-30832 | DQ570720 | AGAACAAGGAGCATGTGATTGAGGCC | 26 |
| 97 | piR-34291 | DQ596225 | GACAATGAATACGTGTCTGGAACTCTGAGG | 30 |
| 98 | piR-56022 | DQ588910 | TGGGATGTGAGGAGCCCCTCTGCCTGGCTGC | 31 |
| 99 | piR-36329 | DQ598263 | GGTCAGTCGGTCCTGAGAGATGGGCGAGC | 29 |
| 100 | piR-61298 | DQ595186 | TTGCTGTGATGACTATCTTAGGACACCTTTG | 31 |
| 101 | piR-34358 | DQ596292 | GACATTGGTGGTTCAGTGGTAGAATTCT | 28 |
| 102 | piR-33864 | DQ593752 | CTGCATCCACTGATAGACCTTGAACAAT | 28 |
| 103 | piR-31111 | DQ570999 | AGCCTATGATGGTTAGTTATCCCTGTCTGAAA | 32 |
| 104 | piR-34668 | DQ596602 | GAGCATGGTAATGGATTTATGGTGGGTC | 28 |
| 105 | piR-33487 | DQ593375 | CGGGAGGCCCGGGTTCGTTTCCCGGCCAATG | 31 |
| 106 | piR-41464 | DQ573352 | TCAGACATTTGGTGTATGTGCTTGGC | 26 |
| 107 | piR-31987 | DQ571875 | CAACAATAGGGTTTACGACCTCGATGTTGGA | 31 |
| 108 | piR-36225 | DQ598159 | GGGGATGTAGCTCAGTGGTAGAGCGCATGCT | 31 |
| 109 | piR-34604 | DQ596538 | GAGAGGGGCCCGTGCCTTGGAAAGCGTCGCG | 31 |
| 110 | piR-60668 | DQ594556 | TTCGATGAAGAGATGATGACGAGTCTGACT | 30 |
| 111 | piR-35407 | DQ597341 | GCCCGGATGATCCTCAGTGGTCTGGGGTGC | 30 |
| 112 | piR-35548 | DQ597482 | GCTAAAAGAGCACACCCGTCTATGTAGCAAA | 31 |
| 113 | piR-34443 | DQ596377 | GACGAGGTGGCCGAGTGGTTAAGGCTATGGAC | 32 |
| 114 | piR-56321 | DQ589209 | TGGGCTTGTCTCTCTGAAAATTGGAGT | 27 |
| 115 | piR-34376 | DQ596310 | GACCAATGATGAGATTGGAGGGTGTCTGAA | 30 |
| 116 | piR-44720 | DQ576608 | TCCTTAGGTCGCTGGTTCGTTTCCGGCTCGA | 31 |
| 117 | piR-31142 | DQ571030 | AGCGTTGGTGGTATAGTGGTGAGCATAGC | 29 |
| 118 | piR-36716 | DQ598650 | GTTCAGTGATGAGGCCTGGAATGTGCGCTGGG | 32 |
| 119 | piR-31068 | DQ570956 | AGCATTGGTGGTTCAGTGGTAGAATTCTCGC | 31 |
| 120 | piR-36037 | DQ597971 | GGCCGGTTAGCTCAGTTGGTTAGAGC | 26 |
| 121 | piR-31650 | DQ571538 | ATCAATGATGAGTACCCTGGGGTGTCT | 27 |
| 122 | piR-50786 | DQ583674 | TGCAGCCGTGTCAAATTCAGTACCTGTCCT | 30 |
| 123 | piR-58119 | DQ591007 | TGTCATGCGGCCGGAGCAGATCATGAAGTCCA | 32 |
| 124 | piR-30884 | DQ570772 | AGACACTCGTGGAGGCGTCGGCAGAGC | 27 |
| 125 | piR-30652 | DQ570540 | ACCTGATGTTACATTGTAGTGTGCTGATG | 29 |
| 126 | piR-36338 | DQ598272 | GGTCGATGATGATTGGTAAAAGGTCTGATA | 30 |
| 127 | piR-36339 | DQ598273 | GGTCGCTGGTTCGAATCCGGCTCGAAGGACC | 31 |
| 128 | piR-40982 | DQ572870 | TCACAAAGATGAGTGGTGAAAATCTGATC | 29 |
| 129 | piR-57498 | DQ590386 | TGGTGGTTCAGTGGTAGAATTCTCGCCTG | 29 |
| 130 | piR-33879 | DQ593767 | CTGCGATGATGGCATTTCTTAGGACACCTTTG | 32 |
| 131 | piR-54381 | DQ587269 | TGGATATGATGACTGATTACCTGAGA | 26 |
| 132 | piR-35413 | DQ597347 | GCCCGGCTAGCTCAGTCGGTAGAGCATGAGAC | 32 |
| 133 | piR-52404 | DQ585292 | TGCGCATGAATGAATGAACGACGGTGTT | 28 |
| 134 | piR-36196 | DQ598130 | GGGCATACTCGTAGACCTTGCCTGACTG | 28 |
| 135 | piR-35551 | DQ597485 | GCTAAACCTAGCCCCAAACCCACTCCACCCT | 31 |
| 136 | piR-55152 | DQ588040 | TGGCGATGAGGAGGTACCTATTGTGTTGAGTA | 32 |
| 137 | piR-55151 | DQ588039 | TGGCGATGAGGAGGTACCTATTGTGTTGAGT | 31 |
| 138 | piR-33650 | DQ593538 | CTCACAAAGATGAGTGGTGAAAATCTGATC | 30 |
| 139 | piR-34736 | DQ596670 | GAGGAATGATGACAAGAAAAGGCCGAA | 27 |
| 140 | piR-33082 | DQ592970 | CCCGGCCCGGACACGGACAGGATTGACAGATT | 32 |
| 141 | piR-31179 | DQ571067 | AGCTGGAGTGCAGTGGTGCGATCACGGC | 28 |
| 142 | piR-30890 | DQ570778 | AGACAGGTTAGTTTTACCCTACTGATGATGT | 31 |
| 143 | piR-32374 | DQ582262 | CAGAGTCGCGCAGCGGAAGCGTGCTGGGCCC | 31 |
| 144 | piR-32678 | DQ582566 | CATTGATCATCGACACTTCGAACGCACTTG | 30 |
| 145 | piR-57984 | DQ590872 | TGTATTCCTGAACTGGAGCCCCAGAC | 26 |
| 146 | piR-31701 | DQ571589 | ATCGAGGCTAGAGTCACGCTTGGGTATCGGCT | 32 |
| 147 | piR-36026 | DQ597960 | GGCCCCATGGTGTAATGGTCAGCACTC | 27 |
| 148 | piR-31703 | DQ571591 | ATCGCTGTACGGCCTTGATGAAAGCA | 26 |
| 149 | piR-52016 | DQ584904 | TGCCTAGTGGGCCACTTTTGGTAAGCAGAA | 30 |
| 150 | piR-42844 | DQ574732 | TCCAGGTTCGACTCCTGGCTGGCTCGC | 27 |
| 151 | piR-37213 | DQ599147 | TAACGCCAAGGTCGCGGGTTCGAACCCCGTA | 31 |
| 152 | piR-48517 | DQ580405 | TGAGAGTGATGAGTTGCACACTGGTGG | 27 |
| 153 | piR-30810 | DQ570698 | ACTTGTGATGTCTTCAAAGGAACCACTGATG | 31 |
| 154 | piR-47305 | DQ579193 | TGAATCTGACAACAGAGGCTTACGACCCCTTA | 32 |
| 155 | piR-31447 | DQ571335 | AGGGGCTGAATGAAAATGGCCTTTCTGAAC | 30 |
| 156 | piR-41209 | DQ573097 | TCACCCGGCCCGGACACGGACAGGATTGACA | 31 |
| 157 | piR-35953 | DQ597887 | GGCAGATGATGTCCTTATCTCACGAT | 26 |
| 158 | piR-35952 | DQ597886 | GGCAGAGTGGCGCAGCGGAAGCGTGCTGGGCC | 32 |
| 159 | piR-30318 | DQ570206 | AATGCAGTGTGGAACACAATGAACTGAAC | 29 |
| 160 | piR-38145 | DQ600079 | TACCAATGATGAGATTGGAGGGTGTCTGAAT | 31 |
| 161 | piR-30504 | DQ570392 | ACCAATGATGAGACAGTGTTTATGAA | 26 |
| 162 | piR-36444 | DQ598378 | GTATATGGCATGTGGGCTAGTTTCAGACAGGT | 32 |
| 163 | piR-30506 | DQ570394 | ACCAATGATGAGATTGGAGGGTGTCTGAAT | 30 |
| 164 | piR-44984 | DQ576872 | TCGCCGTGATCGTATAGTGGTTAGTACTCTG | 31 |
| 165 | piR-36441 | DQ598375 | GTAGTCGTGGCCGAGTGGTTAAGGCTATGGA | 31 |
| 166 | piR-34377 | DQ596311 | GACCAATGATGAGATTGGAGGGTGTCTGAAT | 31 |
| 167 | piR-33415 | DQ593303 | CGCGGGTTCGATCCCCGTACGGGCCACC | 28 |
| 168 | piR-35469 | DQ597403 | GCCTGGGTAGCTCAGTCGGTAGAGCATCAGAC | 32 |
| 169 | piR-35545 | DQ597479 | GCGTTGGTATAGTGGTGAGCATAGCTGC | 28 |
| 170 | piR-46895 | DQ578783 | TGAACATGGGTCAGTCGGTCCTGAGA | 26 |
| 171 | piR-33151 | DQ593039 | CCGCCTGGGAATACCGGGTGCTGTAGGCTTA | 31 |
| 172 | piR-34420 | DQ596354 | GACCTATGATGATGACTGGTGGCGTATGAGT | 31 |
| 173 | piR-60852 | DQ594740 | TTCTGGGTCGGGGTTTCGTACGTAGCA | 27 |
| 174 | piR-34811 | DQ596745 | GAGGGTCCAGGGTTCATGTCCCTGTTCAG | 29 |
| 175 | piR-31963 | DQ571851 | CAAAGCAGCTGCTGCACCTGTGCCTG | 26 |
| 176 | piR-50725 | DQ583613 | TGCAGAGTGGCGCAGCGGAAGCGTGCTGG | 29 |
| 177 | piR-41435 | DQ573323 | TCAGAAGATTCCAGGTTCGACTCCTGGC | 28 |
| 178 | piR-36041 | DQ597975 | GGCCGTGATCGTATAGTGGTTAGTACTCTG | 30 |
| 179 | piR-36056 | DQ597990 | GGCGACAAACCTACCGAGCCTGGTGATAG | 29 |
| 180 | piR-36741 | DQ598675 | GTTTAGACGGGCTCACATCACCCCATAAACA | 31 |
| 181 | piR-34221 | DQ596155 | GAAGGTTGACGAAAATTCTTACTGAGCA | 28 |
| 182 | piR-30961 | DQ570849 | AGAGTTGCGCAGCGGAAGCGTGCTGGGCCCA | 31 |
| 183 | piR-61919 | DQ595807 | TTTCTGTGTGGAATTTGAATATCTGAAA | 28 |
| 184 | piR-34249 | DQ596183 | GAATGCAGCCCAAAGCGGGTGGTAAACT | 28 |
| 185 | piR-30105 | DQ569993 | AAATGCAGTGTGGAACACAATGAACTGAAC | 30 |
| 186 | piR-36376 | DQ598310 | GGTTAGTTTTACCCTACTGATGATGTGTTGTT | 32 |
| 187 | piR-57516 | DQ590404 | TGGTGTATGTGCTTGGCTGAGGAGCCAATGG | 31 |
| 188 | piR-36378 | DQ598312 | GGTTCCATGGTGTAATGGTTAGCACTCTG | 29 |
| 189 | piR-33543 | DQ593431 | CGTGCTGGGCCCATAACCCAGAGGTCGATGGA | 32 |
| 190 | piR-31052 | DQ570940 | AGCAGTTGAACATGGGTCAGTCGGTCCTG | 29 |
| 191 | piR-31115 | DQ571003 | AGCCTGAGCAACATAGCGAGACCCCGTCTCTA | 32 |
| 192 | piR-60238 | DQ594126 | TTCACTGATGAGAGCATTGTTCTGAGC | 27 |
| 193 | piR-35304 | DQ597238 | GCCAAGGTCGCGGGTTCGGTCCCCGTACGGG | 31 |
| 194 | piR-31368 | DQ571256 | AGGCTCGTTGGTCTAGTGGTATGATTCTCG | 30 |
| 195 | piR-43996 | DQ575884 | TCCGTAGTGTAGTGGTTATCACGTTCGCCTGA | 32 |
| 196 | piR-43997 | DQ575885 | TCCGTAGTGTAGTGGTTATCACTTTCGCCT | 30 |
| 197 | piR-32311 | DQ582199 | CAGAAGATTGAGGGTTCGTGTCCCTTCGTGGT | 32 |
| 198 | piR-33783 | DQ593671 | CTGAACTCCTCACACCCAATTGGACCA | 27 |
| 199 | piR-36040 | DQ597974 | GGCCGTGATCGTATAGTGGTTAGTACTC | 28 |
| 200 | piR-54265 | DQ587153 | TGGAGGTGATGAACTGTCTGAGCCTGACC | 29 |
| 201 | piR-36511 | DQ598445 | GTCGGGTTGCTTGGGAATGCAGCCCAAA | 28 |
| 202 | piR-48966 | DQ580854 | TGAGGAGCCAATGGGGCGAAGCTACCATC | 29 |
| 203 | piR-43994 | DQ575882 | TCCGTAGTGTAGTGGTTATCACGTTCGCCTCA | 32 |
| 204 | piR-31925 | DQ571813 | ATTGGTGGTTCAGTGGTAGAATTCTCGCCTG | 31 |
| 205 | piR-34789 | DQ596723 | GAGGCGGGCATGACACAGCAAGACGAGAAG | 30 |
| 206 | piR-49124 | DQ581012 | TGAGGGTTCGAGTCCCTTCGTGGTCGCC | 28 |
| 207 | piR-33949 | DQ593837 | CTGGGAATGCAGCCCAAAGCGGGTGGTAA | 29 |
| 208 | piR-30112 | DQ570000 | AAATGTTATGATGATGGGCGAAATGTTCAACT | 32 |
| 209 | piR-30113 | DQ570001 | AAATGTTGGTTATACCCTTCCCGTACTACC | 30 |
| 210 | piR-54907 | DQ587795 | TGGCCAAGGATGAGAACTCTAATCTGAAA | 29 |
| 211 | piR-60573 | DQ594461 | TTCCGGGTTCGAGTCCCGGCGGAGTCGCC | 29 |
| 212 | piR-30926 | DQ570814 | AGAGATAGCAGAGTGGCGCAGCGGAAGC | 28 |
| 213 | piR-35229 | DQ597163 | GCAGTGGCGCAGCGGAAGCGTGCTGGGCC | 29 |
| 214 | piR-34533 | DQ596467 | GAGAAAGCTCACAAGAACTGCTAACTCACC | 30 |
| 215 | piR-36340 | DQ598274 | GGTCGCTGGTTCGTTTCCGGCTCGAAGGACC | 31 |
| 216 | piR-53542 | DQ586430 | TGGAAAGGATGAAGAGCTGACTGATGGAA | 29 |
| 217 | piR-33382 | DQ593270 | CGCACGTGTTAGGACCCGAAAGATGGTGAAC | 31 |
| 218 | piR-33387 | DQ593275 | CGCAGAGTCGCGCAGCGGAAGCGTGCTGGGCC | 32 |
| 219 | piR-56450 | DQ589338 | TGGGGGGGCCCAAGTCCTTCTGATCGAGG | 29 |

Table S1B. Annotated piRNAs identified in ENOCa

| **S.No** | **piRNA** | **NCBI Accession** | **piRNA sequence** | **Length** |
| --- | --- | --- | --- | --- |
| 1 | piR-33437 | DQ593325 | CGGAAGCGTGCTGGGCCCATAACCCAGA | 28 |
| 2 | piR-43768 | DQ575656 | TCCCTGGTAGTCTAGTGGTTAGGATTC | 27 |
| 3 | piR-41947 | DQ573835 | TCATGAGGTCAGGAGTTCAAGACCAGCCA | 29 |
| 4 | piR-60565 | DQ594453 | TTCCCTGGTGGTCTAGTGGTTAGGATTCGGC | 31 |
| 5 | piR-36173 | DQ598107 | GGGAGGCCCGGGTTCGTTTCCCGGCCAATGCA | 32 |
| 6 | piR-33470 | DQ593358 | CGGCTGTTAACCGAAAGGTTGGTGGT | 26 |
| 7 | piR-61648 | DQ595536 | TTGGTGGTTCAGTGGTAGAATTCTCGCCTGCC | 32 |
| 8 | piR-61404 | DQ595292 | TTGGAGGATGAAACAAAGGAATCTGACT | 28 |
| 9 | piR-61646 | DQ595534 | TTGGTGGTTCAGTGGTAGAATTCTCGCCTG | 30 |
| 10 | piR-46944 | DQ578832 | TGAACCTGGGAGGCGGAGGTTGCAGTGAGC | 30 |
| 11 | piR-35709 | DQ597643 | GCTTGGTGGGACCTGACGAGTTGGTGT | 27 |
| 12 | piR-35284 | DQ597218 | GCATTGGTGGTTCAGTGGTAGAATTCTCAC | 30 |
| 13 | piR-36706 | DQ598640 | GTTAACCGAAAGGTTGGTGGTTCGTGCCCA | 30 |
| 14 | piR-39018 | DQ600952 | TAGAGCATGAGACTCTTAATCTCAGGGTCGTG | 32 |
| 15 | piR-36063 | DQ597997 | GGCGGGAGTAACTATGACTCTCTTAAGGTA | 30 |
| 16 | piR-38240 | DQ600174 | TACCATCTTGGCTCACTGCAACCTCCGCCT | 30 |
| 17 | piR-43604 | DQ575492 | TCCCGGCTAGCTCAGTCGGTAGAGCATGA | 29 |
| 18 | piR-52207 | DQ585095 | TGCCTGTAATCCCAGCTACTCAGGAGGCTG | 30 |
| 19 | piR-32539 | DQ582427 | CAGGCTCATAGGTAGAAGGGACTTGCCTTG | 30 |
| 20 | piR-35176 | DQ597110 | GCAGAGTGGCGCAGCGGAAGCGTGCTGGGCCC | 32 |
| 21 | piR-57132 | DQ590020 | TGGTCTCAAACTCCTGACCTCAGGTGATCT | 30 |
| 22 | piR-36712 | DQ598646 | GTTCACTGATGAGAGCATTGTTCTGAGCCA | 30 |
| 23 | piR-48389 | DQ580277 | TGAGACGCAGTTTCGCTCTTGTTGCCCAG | 29 |
| 24 | piR-31531 | DQ571419 | AGTAAGTGAAGATAAAGTGTGTCTGAGG | 28 |
| 25 | piR-57816 | DQ590704 | TGTAATCCCAGCACTTTGGGAGGCCGAGG | 29 |
| 26 | piR-35982 | DQ597916 | GGCATTGGTGGTTCAGTGGTAGAATTCTCGC | 31 |
| 27 | piR-52206 | DQ585094 | TGCCTGTAATCCCAGCACTTTGGGAGGCCG | 30 |
| 28 | piR-45012 | DQ576900 | TCGCTCACGCTGGGAGCTGTAGACCGGAGC | 30 |
| 29 | piR-60577 | DQ594465 | TTCCGTAGTGTAGTGGTTATCACGTTCGCCTC | 32 |
| 30 | piR-60576 | DQ594464 | TTCCGTAGTGTAGTGGTTATCACGTTCGCC | 30 |
| 31 | piR-35952 | DQ597886 | GGCAGAGTGGCGCAGCGGAAGCGTGCTGGGCC | 32 |
| 32 | piR-33468 | DQ593356 | CGGCTAGCTCAGTCGGTAGAGCATGAGACT | 30 |
| 33 | piR-32214 | DQ582102 | CACCTGAGGTCGGGAGTTCAAGACCAGC | 28 |
| 34 | piR-34358 | DQ596292 | GACATTGGTGGTTCAGTGGTAGAATTCT | 28 |
| 35 | piR-50437 | DQ583325 | TGCAATGGCATGATCTCGGCTCACTGC | 27 |
| 36 | piR-46079 | DQ577967 | TCTGCTGCCTCAGCCTCCCGAGTAGCTGA | 29 |
| 37 | piR-33487 | DQ593375 | CGGGAGGCCCGGGTTCGTTTCCCGGCCAATG | 31 |
| 38 | piR-38581 | DQ600515 | TACTCAGGAGGCTGAGGCAGGAGAATGGC | 29 |
| 39 | piR-36444 | DQ598378 | GTATATGGCATGTGGGCTAGTTTCAGACAGGT | 32 |
| 40 | piR-55891 | DQ588779 | TGGGAGGCGGAGGTTGCAGTGAGCCGAGA | 29 |
| 41 | piR-31068 | DQ570956 | AGCATTGGTGGTTCAGTGGTAGAATTCTCGC | 31 |
| 42 | piR-61861 | DQ595749 | TTTCAAGTGATTCTCCTGTCTCAGCCTCC | 29 |
| 43 | piR-35413 | DQ597347 | GCCCGGCTAGCTCAGTCGGTAGAGCATGAGAC | 32 |
| 44 | piR-52404 | DQ585292 | TGCGCATGAATGAATGAACGACGGTGTT | 28 |
| 45 | piR-32374 | DQ582262 | CAGAGTCGCGCAGCGGAAGCGTGCTGGGCCC | 31 |
| 46 | piR-33486 | DQ593374 | CGGGAGGCCCGGGTTCGGTTCCCGGCCAATGC | 32 |
| 47 | piR-43939 | DQ575827 | TCCGCTTCCCGGGTTCAAGCGATTCTCCTGC | 31 |
| 48 | piR-32512 | DQ582400 | CAGGAGTTCAAGACCAGCCTGGCCAACGTA | 30 |
| 49 | piR-38736 | DQ600670 | TACTTGAGAGGCTGAGGCAGGAGGATCGCT | 30 |
| 50 | piR-44984 | DQ576872 | TCGCCGTGATCGTATAGTGGTTAGTACTCTG | 31 |
| 51 | piR-43103 | DQ574991 | TCCCAAAGTGCTAGGATTACAGGAGTGAG | 29 |
| 52 | piR-34896 | DQ596830 | GAGTTTGAGACCAGCCTGGCCAACATGGT | 29 |
| 53 | piR-30961 | DQ570849 | AGAGTTGCGCAGCGGAAGCGTGCTGGGCCCA | 31 |
| 54 | piR-50725 | DQ583613 | TGCAGAGTGGCGCAGCGGAAGCGTGCTGG | 29 |
| 55 | piR-50444 | DQ583332 | TGCAATGGTGCGATCTCGGCTCACTGCA | 28 |
| 56 | piR-57567 | DQ590455 | TGGTGTGATCTCGGCTCACTGCAACCTCCT | 30 |
| 57 | piR-56501 | DQ589389 | TGGGNGGCAGAGGTTGCAGTGAGCCAAGA | 29 |
| 58 | piR-33543 | DQ593431 | CGTGCTGGGCCCATAACCCAGAGGTCGATGGA | 32 |
| 59 | piR-40666 | DQ572554 | TCAAACTCCTGACCTCATGATCCGCCTGCCT | 31 |
| 60 | piR-31115 | DQ571003 | AGCCTGAGCAACATAGCGAGACCCCGTCTCTA | 32 |
| 61 | piR-41936 | DQ573824 | TCATCTTGCCTGAGCTCTACCTGGACC | 27 |
| 62 | piR-59592 | DQ592480 | TNCAGACCAAGCTCATGACTCACAATGGCC | 30 |
| 63 | piR-43997 | DQ575885 | TCCGTAGTGTAGTGGTTATCACTTTCGCCT | 30 |
| 64 | piR-46628 | DQ578516 | TGAAAAGTACCAAGAAGTGGAAGAAGACC | 29 |
| 65 | piR-39980 | DQ601914 | TAGTCCCAGCTACTTGGGAGGCTGAGGCA | 29 |
| 66 | piR-60238 | DQ594126 | TTCACTGATGAGAGCATTGTTCTGAGC | 27 |
| 67 | piR-52966 | DQ585854 | TGCTGAAGTTTGCTGCTGCCACTGGAGCC | 29 |
| 68 | piR-43994 | DQ575882 | TCCGTAGTGTAGTGGTTATCACGTTCGCCTCA | 32 |
| 69 | piR-31925 | DQ571813 | ATTGGTGGTTCAGTGGTAGAATTCTCGCCTG | 31 |
| 70 | piR-30318 | DQ570206 | AATGCAGTGTGGAACACAATGAACTGAAC | 29 |
| 71 | piR-36245 | DQ598179 | GGGGGTATAGCTCAGTGGCAGAGCATTTGA | 30 |
| 72 | piR-36037 | DQ597971 | GGCCGGTTAGCTCAGTTGGTTAGAGC | 26 |
| 73 | piR-44610 | DQ576498 | TCCTGGGTTCAGGTGATTCTCCTGCCTCAGT | 31 |
| 74 | piR-30121 | DQ570009 | AACAATAGCAATTGGGCTGGCTTAGGAGC | 29 |
| 75 | piR-36074 | DQ598008 | GGCTCGTTGGTCTAGGGGTATGATTCTCGG | 30 |
| 76 | piR-34871 | DQ596805 | GAGTAGAGTGCTTAGTTGAACAGGGCC | 27 |
| 77 | piR-34653 | DQ596587 | GAGCACTGTTCGTAACCCGTTAGCCT | 26 |
| 78 | piR-34443 | DQ596377 | GACGAGGTGGCCGAGTGGTTAAGGCTATGGAC | 32 |
| 79 | piR-40673 | DQ572561 | TCAAAGAATTGGGTGACCACGTGACCAACTT | 31 |
| 80 | piR-31985 | DQ571873 | CAACAAGTACCGTAAGGGAAAGTTGA | 26 |
| 81 | piR-31638 | DQ571526 | ATAGGTTTGGTCCTAGCCTTTCTATTAGCTCT | 32 |
| 82 | piR-36041 | DQ597975 | GGCCGTGATCGTATAGTGGTTAGTACTCTG | 30 |
| 83 | piR-60668 | DQ594556 | TTCGATGAAGAGATGATGACGAGTCTGACT | 30 |
| 84 | piR-57172 | DQ590060 | TGGTCTTGAACTCCTGGCCTCGGGTGATAT | 30 |
| 85 | piR-36038 | DQ597972 | GGCCGTGATCGTATAGTGGTTAGTAC | 26 |
| 86 | piR-31701 | DQ571589 | ATCGAGGCTAGAGTCACGCTTGGGTATCGGCT | 32 |
| 87 | piR-31447 | DQ571335 | AGGGGCTGAATGAAAATGGCCTTTCTGAAC | 30 |
| 88 | piR-35548 | DQ597482 | GCTAAAAGAGCACACCCGTCTATGTAGCAAA | 31 |
| 89 | piR-34811 | DQ596745 | GAGGGTCCAGGGTTCATGTCCCTGTTCAG | 29 |
| 90 | piR-34249 | DQ596183 | GAATGCAGCCCAAAGCGGGTGGTAAACT | 28 |
| 91 | piR-36378 | DQ598312 | GGTTCCATGGTGTAATGGTTAGCACTCTG | 29 |
| 92 | piR-33949 | DQ593837 | CTGGGAATGCAGCCCAAAGCGGGTGGTAA | 29 |
| 93 | piR-36040 | DQ597974 | GGCCGTGATCGTATAGTGGTTAGTACTC | 28 |
| 94 | piR-36511 | DQ598445 | GTCGGGTTGCTTGGGAATGCAGCCCAAA | 28 |
| 95 | piR-35281 | DQ597215 | GCATTGGTGGTATAGTGGTAAGCATAGC | 28 |
| 96 | piR-36499 | DQ598433 | GTCCGGTGCGGAGTGCCCTTCGTCCTGGGAA | 31 |
| 97 | piR-51657 | DQ584545 | TGCCATGGTAATCCTGCTCAGTACGAGA | 28 |
| 98 | piR-31080 | DQ570968 | AGCCAATGGGGCGAAGCTACCATCTGT | 27 |
| 99 | piR-43773 | DQ575661 | TCCCTGGTTCGATCCCGGGTTTCGGCACC | 29 |
| 100 | piR-36233 | DQ598167 | GGGGCGAAGCTACCATCTGTGGGATT | 26 |
| 101 | piR-36225 | DQ598159 | GGGGATGTAGCTCAGTGGTAGAGCGCATGCT | 31 |
| 102 | piR-60852 | DQ594740 | TTCTGGGTCGGGGTTTCGTACGTAGCA | 27 |
| 103 | piR-48966 | DQ580854 | TGAGGAGCCAATGGGGCGAAGCTACCATC | 29 |
| 104 | piR-33864 | DQ593752 | CTGCATCCACTGATAGACCTTGAACAAT | 28 |
| 105 | piR-34374 | DQ596308 | GACCAATGATGAGACAGTGTTTATGAAA | 28 |
| 106 | piR-45120 | DQ577008 | TCGGCATCAATATGGTGACCTCCCGGG | 27 |
| 107 | piR-61135 | DQ595023 | TTGCAAGCAACACTCTGTGGCAGATGATC | 29 |
| 108 | piR-36318 | DQ598252 | GGTAGTGTGGCCGAGCGGTCTAAGGC | 26 |
| 109 | piR-44717 | DQ576605 | TCCTTAGGTCGCTGGTTCGATTCCGGCTCGAA | 32 |
| 110 | piR-43176 | DQ575064 | TCCCAATGATGAGTTGCCATGCTAATACTGA | 31 |
| 111 | piR-33044 | DQ592932 | CCCCCCACTGCTAAATTTGACTGGTT | 26 |
| 112 | piR-44718 | DQ576606 | TCCTTAGGTCGCTGGTTCGGTTCCGGCTCGA | 31 |
| 113 | piR-47274 | DQ579162 | TGAATAGCTGGGACTACAGGTGTGTGCCACT | 31 |
| 114 | piR-35058 | DQ596992 | GCAATAACAGGTCTGTGATGCCCTTAGA | 28 |
| 115 | piR-34375 | DQ596309 | GACCAATGATGAGACAGTGTTTATGAAC | 28 |
| 116 | piR-34456 | DQ596390 | GACGGTGAATACAGGTCTGGAAGTCTGAGGT | 31 |
| 117 | piR-34929 | DQ596863 | GATCGATGATGACTACCGGTGGCGTATGAGT | 31 |
| 118 | piR-57947 | DQ590835 | TGTAGTGCGCTATGCCGATCGGGTGTCC | 28 |
| 119 | piR-34291 | DQ596225 | GACAATGAATACGTGTCTGGAACTCTGAGG | 30 |
| 120 | piR-34377 | DQ596311 | GACCAATGATGAGATTGGAGGGTGTCTGAAT | 31 |
| 121 | piR-42844 | DQ574732 | TCCAGGTTCGACTCCTGGCTGGCTCGC | 27 |
| 122 | piR-38245 | DQ600179 | TACCATGCTGTTTTGGTTGCTGTGGCCTA | 29 |
| 123 | piR-44940 | DQ576828 | TCGATCTCCTGACCTCGTGATCGGCCCGTCTC | 32 |
| 124 | piR-41435 | DQ573323 | TCAGAAGATTCCAGGTTCGACTCCTGGC | 28 |
| 125 | piR-34420 | DQ596354 | GACCTATGATGATGACTGGTGGCGTATGAGT | 31 |
| 126 | piR-35304 | DQ597238 | GCCAAGGTCGCGGGTTCGGTCCCCGTACGGG | 31 |
| 127 | piR-31368 | DQ571256 | AGGCTCGTTGGTCTAGTGGTATGATTCTCG | 30 |
| 128 | piR-36717 | DQ598651 | GTTCATGATGACACAGGACCTTGTCTGAAC | 30 |
| 129 | piR-34221 | DQ596155 | GAAGGTTGACGAAAATTCTTACTGAGCA | 28 |
| 130 | piR-32950 | DQ582838 | CCCACTCCCAGGGCACCATATTGATGCCGAAC | 32 |
| 131 | piR-31703 | DQ571591 | ATCGCTGTACGGCCTTGATGAAAGCA | 26 |
| 132 | piR-41004 | DQ572892 | TCACAATGCTGACACTCAAACTGCTGACA | 29 |
| 133 | piR-35531 | DQ597465 | GCGGTGACACTGTATAAACACGATGACC | 28 |
| 134 | piR-58255 | DQ591143 | TGTCGCACTCGGACGAGAAGCCCTACCAGT | 30 |
| 135 | piR-30451 | DQ570339 | ACAGGTCTGTGATGCCCTTAGATGTCCGG | 29 |
| 136 | piR-32311 | DQ582199 | CAGAAGATTGAGGGTTCGTGTCCCTTCGTGGT | 32 |
| 137 | piR-33686 | DQ593574 | CTCAGTGATGCAATCTCTGTGTGGTTCTGAGA | 32 |
| 138 | piR-30438 | DQ570326 | ACAGCAGTTGAACATGGGTCAGTCGGTCC | 29 |
| 139 | piR-31623 | DQ571511 | ATAACTGACGAAGACTACTCCTGTCTGATT | 30 |
| 140 | piR-54998 | DQ587886 | TGGCCATGTTGGTCAGGCTGGTCTCGAACT | 30 |
| 141 | piR-36494 | DQ598428 | GTCCATGATGATTTCAAGTTATCCCTGTCTGA | 32 |
| 142 | piR-31052 | DQ570940 | AGCAGTTGAACATGGGTCAGTCGGTCCTG | 29 |
| 143 | piR-48368 | DQ580256 | TGAGACCAATGAAATCGCCAATGCCAAC | 28 |
| 144 | piR-30810 | DQ570698 | ACTTGTGATGTCTTCAAAGGAACCACTGATG | 31 |
| 145 | piR-31970 | DQ571858 | CAAAGTGATTGGTACCTCGTTGTCTGATG | 29 |
| 146 | piR-36441 | DQ598375 | GTAGTCGTGGCCGAGTGGTTAAGGCTATGGA | 31 |
| 147 | piR-46895 | DQ578783 | TGAACATGGGTCAGTCGGTCCTGAGA | 26 |
| 148 | piR-34998 | DQ596932 | GATTATGATGATGCCTTAACACTGACT | 27 |
| 149 | piR-61919 | DQ595807 | TTTCTGTGTGGAATTTGAATATCTGAAA | 28 |
| 150 | piR-61298 | DQ595186 | TTGCTGTGATGACTATCTTAGGACACCTTTG | 31 |
| 151 | piR-33879 | DQ593767 | CTGCGATGATGGCATTTCTTAGGACACCTTTG | 32 |
| 152 | piR-33856 | DQ593744 | CTGCAGTGATGACTTTCTTAGGACACCTTTG | 31 |
| 153 | piR-36011 | DQ597945 | GGCCAGCCTGGTCCACATGGGTCGGAA | 27 |
| 154 | piR-33527 | DQ593415 | CGTCCATGATGTTCCGCAACTACCTACA | 28 |
| 155 | piR-31038 | DQ570926 | AGCAGGACGGTGGCCATGGAAGTCGGAATCC | 31 |
| 156 | piR-31143 | DQ571031 | AGCGTTGGTGGTATAGTGGTGAGCATAGCTGC | 32 |
| 157 | piR-31013 | DQ570901 | AGCACATGATGATTCCAGCGTGCGTCTGAA | 30 |
| 158 | piR-35407 | DQ597341 | GCCCGGATGATCCTCAGTGGTCTGGGGTGC | 30 |
| 159 | piR-31142 | DQ571030 | AGCGTTGGTGGTATAGTGGTGAGCATAGC | 29 |
| 160 | piR-35953 | DQ597887 | GGCAGATGATGTCCTTATCTCACGAT | 26 |
| 161 | piR-31612 | DQ571500 | AGTTCGTGATGGATTTGCTTTTTTCTGATT | 30 |
| 162 | piR-55213 | DQ588101 | TGGCTATGATCTGCCTTGTTCAAGCTGAGA | 30 |
| 163 | piR-36241 | DQ598175 | GGGGGATTAGCTCAAATGGTAGAGCGCTCG | 30 |
| 164 | piR-31636 | DQ571524 | ATAGGGTTTACGACCTCGATGTTGGATC | 28 |
| 165 | piR-30924 | DQ570812 | AGAGAGGGGCCCGTGCCTTGGAAAGCGTC | 29 |
| 166 | piR-34597 | DQ596531 | GAGAGAGGGGCCCGTGCCTTGGAAAGTG | 28 |
| 167 | piR-33065 | DQ592953 | CCCCTGGTGGTCTAGTGGTTAGGATTCGGC | 30 |
| 168 | piR-36329 | DQ598263 | GGTCAGTCGGTCCTGAGAGATGGGCGAGC | 29 |
| 169 | piR-30652 | DQ570540 | ACCTGATGTTACATTGTAGTGTGCTGATG | 29 |
| 170 | piR-36338 | DQ598272 | GGTCGATGATGATTGGTAAAAGGTCTGATA | 30 |
| 171 | piR-54381 | DQ587269 | TGGATATGATGACTGATTACCTGAGA | 26 |
| 172 | piR-34736 | DQ596670 | GAGGAATGATGACAAGAAAAGGCCGAA | 27 |
| 173 | piR-36360 | DQ598294 | GGTGCTGATGACACCCACTGGCTGAAC | 27 |
| 174 | piR-50192 | DQ583080 | TGCAAAAGTTGTTCCTGTGAAAGCCA | 26 |
| 175 | piR-36741 | DQ598675 | GTTTAGACGGGCTCACATCACCCCATAAACA | 31 |
| 176 | piR-43996 | DQ575884 | TCCGTAGTGTAGTGGTTATCACGTTCGCCTGA | 32 |
| 177 | piR-39126 | DQ601060 | TAGATGGAAAGAGGTTGCCGACGTATGA | 28 |
| 178 | piR-41799 | DQ573687 | TCAGTGCCTAGGACTCTGTGTGCCTGACCT | 30 |
| 179 | piR-35059 | DQ596993 | GCAATCACTGATGTCTCCATGTCTCTGAGCA | 31 |
| 180 | piR-40982 | DQ572870 | TCACAAAGATGAGTGGTGAAAATCTGATC | 29 |
| 181 | piR-33650 | DQ593538 | CTCACAAAGATGAGTGGTGAAAATCTGATC | 30 |
| 182 | piR-54265 | DQ587153 | TGGAGGTGATGAACTGTCTGAGCCTGACC | 29 |
| 183 | piR-35545 | DQ597479 | GCGTTGGTATAGTGGTGAGCATAGCTGC | 28 |
| 184 | piR-50701 | DQ583589 | TGCAGACTATTCATTGGGTGTTTGGGGTGT | 30 |
| 185 | piR-30799 | DQ570687 | ACTGTGTGCTGATTGTCACGTTCTGATT | 28 |
| 186 | piR-36716 | DQ598650 | GTTCAGTGATGAGGCCTGGAATGTGCGCTGGG | 32 |
| 187 | piR-43772 | DQ575660 | TCCCTGGTGGTCTAGTGGTTAGGATTCGGCAC | 32 |
| 188 | piR-36196 | DQ598130 | GGGCATACTCGTAGACCTTGCCTGACTG | 28 |
| 189 | piR-30890 | DQ570778 | AGACAGGTTAGTTTTACCCTACTGATGATGT | 31 |
| 190 | piR-36376 | DQ598310 | GGTTAGTTTTACCCTACTGATGATGTGTTGTT | 32 |
| 191 | piR-37649 | DQ599583 | TAATGAAGGCCAGGCTGACAAGATCTCAGA | 30 |
| 192 | piR-30599 | DQ570487 | ACCCGGATGTGACTGGTCGGTTGCTGT | 27 |
| 193 | piR-36743 | DQ598677 | GTTTCCGTAGTGTAGTGGTCATCACGTTCGCC | 32 |
| 194 | piR-34604 | DQ596538 | GAGAGGGGCCCGTGCCTTGGAAAGCGTCGCG | 31 |
| 195 | piR-33519 | DQ593407 | CGTAGTGTAGTGGTCATCACGTTCGCCT | 28 |
| 196 | piR-30840 | DQ570728 | AGAACGTGTGGAAAACTAATGACTGAGC | 28 |
| 197 | piR-43770 | DQ575658 | TCCCTGGTGGTCTAGTGGTTAGGATA | 26 |
| 198 | piR-57125 | DQ590013 | TGGTCGTGGTTGTAGTCCGTGCGAGAA | 27 |
| 199 | piR-33043 | DQ592931 | CCCCCCACTGCTAAATTTGACTGGCTA | 27 |
| 200 | piR-31104 | DQ570992 | AGCCCGGCTAGCTCAGTCGGTAGAGCATGAGA | 32 |
| 201 | piR-33748 | DQ593636 | CTCTACTGAACTGCCATGAGGAAACTGCC | 29 |
| 202 | piR-36252 | DQ598186 | GGGGGTGTAGCTCAGTGGTAGAGAGCGTGCT | 31 |
| 203 | piR-38512 | DQ600446 | TACGTGGACGACACGCAGTTCGTGCGGTTC | 30 |
| 204 | piR-36339 | DQ598273 | GGTCGCTGGTTCGAATCCGGCTCGAAGGACC | 31 |
| 205 | piR-36026 | DQ597960 | GGCCCCATGGTGTAATGGTCAGCACTC | 27 |
| 206 | piR-52016 | DQ584904 | TGCCTAGTGGGCCACTTTTGGTAAGCAGAA | 30 |
| 207 | piR-33415 | DQ593303 | CGCGGGTTCGATCCCCGTACGGGCCACC | 28 |
| 208 | piR-35469 | DQ597403 | GCCTGGGTAGCTCAGTCGGTAGAGCATCAGAC | 32 |
| 209 | piR-33151 | DQ593039 | CCGCCTGGGAATACCGGGTGCTGTAGGCTTA | 31 |
| 210 | piR-59394 | DQ592282 | TGTTGACAACCCCTGAAGAGCCTCAGT | 27 |
| 211 | piR-36685 | DQ598619 | GTGTAGCTCAGTGGTAGAGCGCGTGCTTCGC | 31 |
| 212 | piR-49124 | DQ581012 | TGAGGGTTCGAGTCCCTTCGTGGTCGCC | 28 |
| 213 | piR-31935 | DQ571823 | ATTTGGTGTATGTGCTTGGCTGAGGAGCCAA | 31 |
| 214 | piR-30229 | DQ570117 | AAGCCAGGGATTGTGGGTTCGGGTCCCATCT | 31 |
| 215 | piR-41464 | DQ573352 | TCAGACATTTGGTGTATGTGCTTGGC | 26 |
| 216 | piR-35551 | DQ597485 | GCTAAACCTAGCCCCAAACCCACTCCACCCT | 31 |
| 217 | piR-57516 | DQ590404 | TGGTGTATGTGCTTGGCTGAGGAGCCAATGG | 31 |
| 218 | piR-33536 | DQ593424 | CGTGAGTTCGATCCTCACACGGGGCACCA | 29 |
| 219 | piR-36082 | DQ598016 | GGCTCTGTTGCGCAATGGATAGCGCAT | 27 |
| 220 | piR-51810 | DQ584698 | TGCCCCCATGTCTAACAACATGGCTTTCTCA | 31 |
| 221 | piR-55270 | DQ588158 | TGGCTGAGGCAGGAGGATCACTTGAATC | 28 |
| 222 | piR-59109 | DQ591997 | TGTGTATTCGGACTTCCTGCTCTACA | 26 |
| 223 | piR-55152 | DQ588040 | TGGCGATGAGGAGGTACCTATTGTGTTGAGTA | 32 |
| 224 | piR-55151 | DQ588039 | TGGCGATGAGGAGGTACCTATTGTGTTGAGT | 31 |
| 225 | piR-31106 | DQ570994 | AGCCCTGATGATGCCCACTCCTGAGC | 26 |
| 226 | piR-36170 | DQ598104 | GGGAGATGAAGAGGACAGTGACTGAGAGAC | 30 |
| 227 | piR-52729 | DQ585617 | TGCTATCTGAGAGATGGTGATGACATTA | 28 |
| 228 | piR-51309 | DQ584197 | TGCCAATGATGGTTAAGAATTTCTTCACCTGA | 32 |
| 229 | piR-33466 | DQ593354 | CGGCGGTGGCGGCGGCGGCGGCGGGACC | 28 |
| 230 | piR-36707 | DQ598641 | GTTAAGATGGCAGAGCCCGGTAATCGCATAA | 31 |
| 231 | piR-58469 | DQ591357 | TGTGAATCTGACAACAGAGGCTTACGACCCC | 31 |
| 232 | piR-44892 | DQ576780 | TCGAAGGTGGATTTAGCAGTAAACTGA | 27 |
| 233 | piR-34536 | DQ596470 | GAGAAAGCTCACAAGAACTGCTAACTCATGC | 31 |
| 234 | piR-47305 | DQ579193 | TGAATCTGACAACAGAGGCTTACGACCCCTTA | 32 |
| 235 | piR-31987 | DQ571875 | CAACAATAGGGTTTACGACCTCGATGTTGGA | 31 |
| 236 | piR-36056 | DQ597990 | GGCGACAAACCTACCGAGCCTGGTGATAG | 29 |
| 237 | piR-34789 | DQ596723 | GAGGCGGGCATGACACAGCAAGACGAGAAG | 30 |
| 238 | piR-33783 | DQ593671 | CTGAACTCCTCACACCCAATTGGACCA | 27 |
| 239 | piR-44312 | DQ576200 | TCCTCATTAGTATAGTGGTGAGTATCCC | 28 |
| 240 | piR-30625 | DQ570513 | ACCGTCGTAGTTCCGACCATAAACGATGCC | 30 |
| 241 | piR-32678 | DQ582566 | CATTGATCATCGACACTTCGAACGCACTTG | 30 |
| 242 | piR-41209 | DQ573097 | TCACCCGGCCCGGACACGGACAGGATTGACA | 31 |
| 243 | piR-33082 | DQ592970 | CCCGGCCCGGACACGGACAGGATTGACAGATT | 32 |
| 244 | piR-30112 | DQ570000 | AAATGTTATGATGATGGGCGAAATGTTCAACT | 32 |
| 245 | piR-30113 | DQ570001 | AAATGTTGGTTATACCCTTCCCGTACTACC | 30 |
| 246 | piR-54907 | DQ587795 | TGGCCAAGGATGAGAACTCTAATCTGAAA | 29 |
| 247 | piR-60573 | DQ594461 | TTCCGGGTTCGAGTCCCGGCGGAGTCGCC | 29 |
| 248 | piR-30926 | DQ570814 | AGAGATAGCAGAGTGGCGCAGCGGAAGC | 28 |
| 249 | piR-35229 | DQ597163 | GCAGTGGCGCAGCGGAAGCGTGCTGGGCC | 29 |
| 250 | piR-36340 | DQ598274 | GGTCGCTGGTTCGTTTCCGGCTCGAAGGACC | 31 |
| 251 | piR-33880 | DQ593768 | CTGCGTGTTCAAGTCACGTCGGGGTCAC | 28 |
| 252 | piR-53542 | DQ586430 | TGGAAAGGATGAAGAGCTGACTGATGGAA | 29 |
| 253 | piR-34533 | DQ596467 | GAGAAAGCTCACAAGAACTGCTAACTCACC | 30 |
| 254 | piR-33382 | DQ593270 | CGCACGTGTTAGGACCCGAAAGATGGTGAAC | 31 |
| 255 | piR-33387 | DQ593275 | CGCAGAGTCGCGCAGCGGAAGCGTGCTGGGCC | 32 |
| 256 | piR-56450 | DQ589338 | TGGGGGGGCCCAAGTCCTTCTGATCGAGG | 29 |

Table S1C. Annotated piRNAs identified in SOCa

| **S. No** | **piRNA** | **NCBI Accession** | **piRNA sequence** | **Length** |
| --- | --- | --- | --- | --- |
| 1 | piR-33437 | DQ593325 | CGGAAGCGTGCTGGGCCCATAACCCAGA | 28 |
| 2 | piR-40252 | DQ572140 | TATCCATGGTGTTGAAGCGTCGAGCCGACT | 30 |
| 3 | piR-43768 | DQ575656 | TCCCTGGTAGTCTAGTGGTTAGGATTC | 27 |
| 4 | piR-36173 | DQ598107 | GGGAGGCCCGGGTTCGTTTCCCGGCCAATGCA | 32 |
| 5 | piR-33470 | DQ593358 | CGGCTGTTAACCGAAAGGTTGGTGGT | 26 |
| 6 | piR-61648 | DQ595536 | TTGGTGGTTCAGTGGTAGAATTCTCGCCTGCC | 32 |
| 7 | piR-61404 | DQ595292 | TTGGAGGATGAAACAAAGGAATCTGACT | 28 |
| 8 | piR-61646 | DQ595534 | TTGGTGGTTCAGTGGTAGAATTCTCGCCTG | 30 |
| 9 | piR-36706 | DQ598640 | GTTAACCGAAAGGTTGGTGGTTCGTGCCCA | 30 |
| 10 | piR-33486 | DQ593374 | CGGGAGGCCCGGGTTCGGTTCCCGGCCAATGC | 32 |
| 11 | piR-43994 | DQ575882 | TCCGTAGTGTAGTGGTTATCACGTTCGCCTCA | 32 |
| 12 | piR-36063 | DQ597997 | GGCGGGAGTAACTATGACTCTCTTAAGGTA | 30 |
| 13 | piR-38240 | DQ600174 | TACCATCTTGGCTCACTGCAACCTCCGCCT | 30 |
| 14 | piR-43604 | DQ575492 | TCCCGGCTAGCTCAGTCGGTAGAGCATGA | 29 |
| 15 | piR-52207 | DQ585095 | TGCCTGTAATCCCAGCTACTCAGGAGGCTG | 30 |
| 16 | piR-58707 | DQ591595 | TGTGATTGCACCACTGCACTCCAGCCTGGT | 30 |
| 17 | piR-36712 | DQ598646 | GTTCACTGATGAGAGCATTGTTCTGAGCCA | 30 |
| 18 | piR-31537 | DQ571425 | AGTAGAGACAGGGTTTCACCATGTTGGCCA | 30 |
| 19 | piR-31531 | DQ571419 | AGTAAGTGAAGATAAAGTGTGTCTGAGG | 28 |
| 20 | piR-42491 | DQ574379 | TCCACCTCCCAGATTCAAGTGATTCTCCTGC | 31 |
| 21 | piR-31925 | DQ571813 | ATTGGTGGTTCAGTGGTAGAATTCTCGCCTG | 31 |
| 22 | piR-33733 | DQ593621 | CTCGAACTCCTGACCTCAGGTGATCTGCCT | 30 |
| 23 | piR-60577 | DQ594465 | TTCCGTAGTGTAGTGGTTATCACGTTCGCCTC | 32 |
| 24 | piR-60576 | DQ594464 | TTCCGTAGTGTAGTGGTTATCACGTTCGCC | 30 |
| 25 | piR-35952 | DQ597886 | GGCAGAGTGGCGCAGCGGAAGCGTGCTGGGCC | 32 |
| 26 | piR-34669 | DQ596603 | GAGCATGGTAATGGATTTATGGTGGGTCCTT | 31 |
| 27 | piR-33468 | DQ593356 | CGGCTAGCTCAGTCGGTAGAGCATGAGACT | 30 |
| 28 | piR-34358 | DQ596292 | GACATTGGTGGTTCAGTGGTAGAATTCT | 28 |
| 29 | piR-50437 | DQ583325 | TGCAATGGCATGATCTCGGCTCACTGC | 27 |
| 30 | piR-46079 | DQ577967 | TCTGCTGCCTCAGCCTCCCGAGTAGCTGA | 29 |
| 31 | piR-59425 | DQ592313 | TGTTGCCCAGGCTGGAGTGCAGTAGCGCGA | 30 |
| 32 | piR-33487 | DQ593375 | CGGGAGGCCCGGGTTCGTTTCCCGGCCAATG | 31 |
| 33 | piR-55891 | DQ588779 | TGGGAGGCGGAGGTTGCAGTGAGCCGAGA | 29 |
| 34 | piR-36444 | DQ598378 | GTATATGGCATGTGGGCTAGTTTCAGACAGGT | 32 |
| 35 | piR-41195 | DQ573083 | TCACCCAGGCTAGAGTGCAGTGGTGCA | 27 |
| 36 | piR-31068 | DQ570956 | AGCATTGGTGGTTCAGTGGTAGAATTCTCGC | 31 |
| 37 | piR-61861 | DQ595749 | TTTCAAGTGATTCTCCTGTCTCAGCCTCC | 29 |
| 38 | piR-35413 | DQ597347 | GCCCGGCTAGCTCAGTCGGTAGAGCATGAGAC | 32 |
| 39 | piR-52404 | DQ585292 | TGCGCATGAATGAATGAACGACGGTGTT | 28 |
| 40 | piR-32374 | DQ582262 | CAGAGTCGCGCAGCGGAAGCGTGCTGGGCCC | 31 |
| 41 | piR-43939 | DQ575827 | TCCGCTTCCCGGGTTCAAGCGATTCTCCTGC | 31 |
| 42 | piR-32512 | DQ582400 | CAGGAGTTCAAGACCAGCCTGGCCAACGTA | 30 |
| 43 | piR-38736 | DQ600670 | TACTTGAGAGGCTGAGGCAGGAGGATCGCT | 30 |
| 44 | piR-44984 | DQ576872 | TCGCCGTGATCGTATAGTGGTTAGTACTCTG | 31 |
| 45 | piR-30961 | DQ570849 | AGAGTTGCGCAGCGGAAGCGTGCTGGGCCCA | 31 |
| 46 | piR-35284 | DQ597218 | GCATTGGTGGTTCAGTGGTAGAATTCTCAC | 30 |
| 47 | piR-56501 | DQ589389 | TGGGNGGCAGAGGTTGCAGTGAGCCAAGA | 29 |
| 48 | piR-33543 | DQ593431 | CGTGCTGGGCCCATAACCCAGAGGTCGATGGA | 32 |
| 49 | piR-31115 | DQ571003 | AGCCTGAGCAACATAGCGAGACCCCGTCTCTA | 32 |
| 50 | piR-60238 | DQ594126 | TTCACTGATGAGAGCATTGTTCTGAGC | 27 |
| 51 | piR-43997 | DQ575885 | TCCGTAGTGTAGTGGTTATCACTTTCGCCT | 30 |
| 52 | piR-33221 | DQ593109 | CCTCCCAAAGTGCTGGGATTACAGGCGTGAG | 31 |
| 53 | piR-39980 | DQ601914 | TAGTCCCAGCTACTTGGGAGGCTGAGGCA | 29 |
| 54 | piR-57567 | DQ590455 | TGGTGTGATCTCGGCTCACTGCAACCTCCT | 30 |
| 55 | piR-52966 | DQ585854 | TGCTGAAGTTTGCTGCTGCCACTGGAGCC | 29 |
| 56 | piR-61811 | DQ595699 | TTGTTGAACTTGGATCAGAAGATGATGTA | 29 |
| 57 | piR-30318 | DQ570206 | AATGCAGTGTGGAACACAATGAACTGAAC | 29 |
| 58 | piR-44610 | DQ576498 | TCCTGGGTTCAGGTGATTCTCCTGCCTCAGT | 31 |
| 59 | piR-30105 | DQ569993 | AAATGCAGTGTGGAACACAATGAACTGAAC | 30 |
| 60 | piR-36074 | DQ598008 | GGCTCGTTGGTCTAGGGGTATGATTCTCGG | 30 |
| 61 | piR-34871 | DQ596805 | GAGTAGAGTGCTTAGTTGAACAGGGCC | 27 |
| 62 | piR-34653 | DQ596587 | GAGCACTGTTCGTAACCCGTTAGCCT | 26 |
| 63 | piR-34443 | DQ596377 | GACGAGGTGGCCGAGTGGTTAAGGCTATGGAC | 32 |
| 64 | piR-49900 | DQ581788 | TGATGCCTAAGAAGAACCGGATTGCC | 26 |
| 65 | piR-31985 | DQ571873 | CAACAAGTACCGTAAGGGAAAGTTGA | 26 |
| 66 | piR-31638 | DQ571526 | ATAGGTTTGGTCCTAGCCTTTCTATTAGCTCT | 32 |
| 67 | piR-60668 | DQ594556 | TTCGATGAAGAGATGATGACGAGTCTGACT | 30 |
| 68 | piR-31701 | DQ571589 | ATCGAGGCTAGAGTCACGCTTGGGTATCGGCT | 32 |
| 69 | piR-31447 | DQ571335 | AGGGGCTGAATGAAAATGGCCTTTCTGAAC | 30 |
| 70 | piR-35548 | DQ597482 | GCTAAAAGAGCACACCCGTCTATGTAGCAAA | 31 |
| 71 | piR-34811 | DQ596745 | GAGGGTCCAGGGTTCATGTCCCTGTTCAG | 29 |
| 72 | piR-36040 | DQ597974 | GGCCGTGATCGTATAGTGGTTAGTACTC | 28 |
| 73 | piR-36041 | DQ597975 | GGCCGTGATCGTATAGTGGTTAGTACTCTG | 30 |
| 74 | piR-34249 | DQ596183 | GAATGCAGCCCAAAGCGGGTGGTAAACT | 28 |
| 75 | piR-36378 | DQ598312 | GGTTCCATGGTGTAATGGTTAGCACTCTG | 29 |
| 76 | piR-33949 | DQ593837 | CTGGGAATGCAGCCCAAAGCGGGTGGTAA | 29 |
| 77 | piR-36511 | DQ598445 | GTCGGGTTGCTTGGGAATGCAGCCCAAA | 28 |
| 78 | piR-35281 | DQ597215 | GCATTGGTGGTATAGTGGTAAGCATAGC | 28 |
| 79 | piR-51657 | DQ584545 | TGCCATGGTAATCCTGCTCAGTACGAGA | 28 |
| 80 | piR-31080 | DQ570968 | AGCCAATGGGGCGAAGCTACCATCTGT | 27 |
| 81 | piR-36499 | DQ598433 | GTCCGGTGCGGAGTGCCCTTCGTCCTGGGAA | 31 |
| 82 | piR-43773 | DQ575661 | TCCCTGGTTCGATCCCGGGTTTCGGCACC | 29 |
| 83 | piR-36233 | DQ598167 | GGGGCGAAGCTACCATCTGTGGGATT | 26 |
| 84 | piR-36225 | DQ598159 | GGGGATGTAGCTCAGTGGTAGAGCGCATGCT | 31 |
| 85 | piR-54897 | DQ587785 | TGGCCAACATGGTGAAACCCCGTTTAA | 27 |
| 86 | piR-60852 | DQ594740 | TTCTGGGTCGGGGTTTCGTACGTAGCA | 27 |
| 87 | piR-48966 | DQ580854 | TGAGGAGCCAATGGGGCGAAGCTACCATC | 29 |
| 88 | piR-33864 | DQ593752 | CTGCATCCACTGATAGACCTTGAACAAT | 28 |
| 89 | piR-34375 | DQ596309 | GACCAATGATGAGACAGTGTTTATGAAC | 28 |
| 90 | piR-44720 | DQ576608 | TCCTTAGGTCGCTGGTTCGTTTCCGGCTCGA | 31 |
| 91 | piR-45120 | DQ577008 | TCGGCATCAATATGGTGACCTCCCGGG | 27 |
| 92 | piR-44721 | DQ576609 | TCCTTAGGTCGCTGGTTCGTTTCCGGCTCGAA | 32 |
| 93 | piR-61135 | DQ595023 | TTGCAAGCAACACTCTGTGGCAGATGATC | 29 |
| 94 | piR-36318 | DQ598252 | GGTAGTGTGGCCGAGCGGTCTAAGGC | 26 |
| 95 | piR-43176 | DQ575064 | TCCCAATGATGAGTTGCCATGCTAATACTGA | 31 |
| 96 | piR-33044 | DQ592932 | CCCCCCACTGCTAAATTTGACTGGTT | 26 |
| 97 | piR-44718 | DQ576606 | TCCTTAGGTCGCTGGTTCGGTTCCGGCTCGA | 31 |
| 98 | piR-35058 | DQ596992 | GCAATAACAGGTCTGTGATGCCCTTAGA | 28 |
| 99 | piR-34456 | DQ596390 | GACGGTGAATACAGGTCTGGAAGTCTGAGGT | 31 |
| 100 | piR-34929 | DQ596863 | GATCGATGATGACTACCGGTGGCGTATGAGT | 31 |
| 101 | piR-57947 | DQ590835 | TGTAGTGCGCTATGCCGATCGGGTGTCC | 28 |
| 102 | piR-34291 | DQ596225 | GACAATGAATACGTGTCTGGAACTCTGAGG | 30 |
| 103 | piR-31650 | DQ571538 | ATCAATGATGAGTACCCTGGGGTGTCT | 27 |
| 104 | piR-42844 | DQ574732 | TCCAGGTTCGACTCCTGGCTGGCTCGC | 27 |
| 105 | piR-30506 | DQ570394 | ACCAATGATGAGATTGGAGGGTGTCTGAAT | 30 |
| 106 | piR-34377 | DQ596311 | GACCAATGATGAGATTGGAGGGTGTCTGAAT | 31 |
| 107 | piR-41435 | DQ573323 | TCAGAAGATTCCAGGTTCGACTCCTGGC | 28 |
| 108 | piR-34420 | DQ596354 | GACCTATGATGATGACTGGTGGCGTATGAGT | 31 |
| 109 | piR-35304 | DQ597238 | GCCAAGGTCGCGGGTTCGGTCCCCGTACGGG | 31 |
| 110 | piR-36717 | DQ598651 | GTTCATGATGACACAGGACCTTGTCTGAAC | 30 |
| 111 | piR-34221 | DQ596155 | GAAGGTTGACGAAAATTCTTACTGAGCA | 28 |
| 112 | piR-32950 | DQ582838 | CCCACTCCCAGGGCACCATATTGATGCCGAAC | 32 |
| 113 | piR-41004 | DQ572892 | TCACAATGCTGACACTCAAACTGCTGACA | 29 |
| 114 | piR-31703 | DQ571591 | ATCGCTGTACGGCCTTGATGAAAGCA | 26 |
| 115 | piR-30451 | DQ570339 | ACAGGTCTGTGATGCCCTTAGATGTCCGG | 29 |
| 116 | piR-32311 | DQ582199 | CAGAAGATTGAGGGTTCGTGTCCCTTCGTGGT | 32 |
| 117 | piR-33685 | DQ593573 | CTCAGTGATGCAATCTCTGTGTGGTTCTGAAA | 32 |
| 118 | piR-33686 | DQ593574 | CTCAGTGATGCAATCTCTGTGTGGTTCTGAGA | 32 |
| 119 | piR-30438 | DQ570326 | ACAGCAGTTGAACATGGGTCAGTCGGTCC | 29 |
| 120 | piR-31623 | DQ571511 | ATAACTGACGAAGACTACTCCTGTCTGATT | 30 |
| 121 | piR-54998 | DQ587886 | TGGCCATGTTGGTCAGGCTGGTCTCGAACT | 30 |
| 122 | piR-36494 | DQ598428 | GTCCATGATGATTTCAAGTTATCCCTGTCTGA | 32 |
| 123 | piR-35163 | DQ597097 | GCAGAGATCAAGGCTCTCAAGGAGAAGCTGC | 31 |
| 124 | piR-31052 | DQ570940 | AGCAGTTGAACATGGGTCAGTCGGTCCTG | 29 |
| 125 | piR-31111 | DQ570999 | AGCCTATGATGGTTAGTTATCCCTGTCTGAAA | 32 |
| 126 | piR-30810 | DQ570698 | ACTTGTGATGTCTTCAAAGGAACCACTGATG | 31 |
| 127 | piR-31970 | DQ571858 | CAAAGTGATTGGTACCTCGTTGTCTGATG | 29 |
| 128 | piR-36441 | DQ598375 | GTAGTCGTGGCCGAGTGGTTAAGGCTATGGA | 31 |
| 129 | piR-36249 | DQ598183 | GGGGGTATAGCTCAGTGGTAGAGCATTTGA | 30 |
| 130 | piR-46895 | DQ578783 | TGAACATGGGTCAGTCGGTCCTGAGA | 26 |
| 131 | piR-34998 | DQ596932 | GATTATGATGATGCCTTAACACTGACT | 27 |
| 132 | piR-61919 | DQ595807 | TTTCTGTGTGGAATTTGAATATCTGAAA | 28 |
| 133 | piR-33856 | DQ593744 | CTGCAGTGATGACTTTCTTAGGACACCTTTG | 31 |
| 134 | piR-61298 | DQ595186 | TTGCTGTGATGACTATCTTAGGACACCTTTG | 31 |
| 135 | piR-33879 | DQ593767 | CTGCGATGATGGCATTTCTTAGGACACCTTTG | 32 |
| 136 | piR-33527 | DQ593415 | CGTCCATGATGTTCCGCAACTACCTACA | 28 |
| 137 | piR-31038 | DQ570926 | AGCAGGACGGTGGCCATGGAAGTCGGAATCC | 31 |
| 138 | piR-31143 | DQ571031 | AGCGTTGGTGGTATAGTGGTGAGCATAGCTGC | 32 |
| 139 | piR-36011 | DQ597945 | GGCCAGCCTGGTCCACATGGGTCGGAA | 27 |
| 140 | piR-31013 | DQ570901 | AGCACATGATGATTCCAGCGTGCGTCTGAA | 30 |
| 141 | piR-36189 | DQ598123 | GGGCACGAGTTCGAGGCCAGCCTGGTCCAT | 30 |
| 142 | piR-35407 | DQ597341 | GCCCGGATGATCCTCAGTGGTCTGGGGTGC | 30 |
| 143 | piR-31142 | DQ571030 | AGCGTTGGTGGTATAGTGGTGAGCATAGC | 29 |
| 144 | piR-57984 | DQ590872 | TGTATTCCTGAACTGGAGCCCCAGAC | 26 |
| 145 | piR-31612 | DQ571500 | AGTTCGTGATGGATTTGCTTTTTTCTGATT | 30 |
| 146 | piR-55213 | DQ588101 | TGGCTATGATCTGCCTTGTTCAAGCTGAGA | 30 |
| 147 | piR-36241 | DQ598175 | GGGGGATTAGCTCAAATGGTAGAGCGCTCG | 30 |
| 148 | piR-31636 | DQ571524 | ATAGGGTTTACGACCTCGATGTTGGATC | 28 |
| 149 | piR-30924 | DQ570812 | AGAGAGGGGCCCGTGCCTTGGAAAGCGTC | 29 |
| 150 | piR-45582 | DQ577470 | TCTCCAACAAGAGAATAGTAGGCTGCATC | 29 |
| 151 | piR-33065 | DQ592953 | CCCCTGGTGGTCTAGTGGTTAGGATTCGGC | 30 |
| 152 | piR-36329 | DQ598263 | GGTCAGTCGGTCCTGAGAGATGGGCGAGC | 29 |
| 153 | piR-30652 | DQ570540 | ACCTGATGTTACATTGTAGTGTGCTGATG | 29 |
| 154 | piR-36338 | DQ598272 | GGTCGATGATGATTGGTAAAAGGTCTGATA | 30 |
| 155 | piR-54381 | DQ587269 | TGGATATGATGACTGATTACCTGAGA | 26 |
| 156 | piR-34736 | DQ596670 | GAGGAATGATGACAAGAAAAGGCCGAA | 27 |
| 157 | piR-36741 | DQ598675 | GTTTAGACGGGCTCACATCACCCCATAAACA | 31 |
| 158 | piR-35059 | DQ596993 | GCAATCACTGATGTCTCCATGTCTCTGAGCA | 31 |
| 159 | piR-54265 | DQ587153 | TGGAGGTGATGAACTGTCTGAGCCTGACC | 29 |
| 160 | piR-40982 | DQ572870 | TCACAAAGATGAGTGGTGAAAATCTGATC | 29 |
| 161 | piR-33650 | DQ593538 | CTCACAAAGATGAGTGGTGAAAATCTGATC | 30 |
| 162 | piR-35545 | DQ597479 | GCGTTGGTATAGTGGTGAGCATAGCTGC | 28 |
| 163 | piR-30799 | DQ570687 | ACTGTGTGCTGATTGTCACGTTCTGATT | 28 |
| 164 | piR-36716 | DQ598650 | GTTCAGTGATGAGGCCTGGAATGTGCGCTGGG | 32 |
| 165 | piR-43772 | DQ575660 | TCCCTGGTGGTCTAGTGGTTAGGATTCGGCAC | 32 |
| 166 | piR-36196 | DQ598130 | GGGCATACTCGTAGACCTTGCCTGACTG | 28 |
| 167 | piR-30890 | DQ570778 | AGACAGGTTAGTTTTACCCTACTGATGATGT | 31 |
| 168 | piR-36376 | DQ598310 | GGTTAGTTTTACCCTACTGATGATGTGTTGTT | 32 |
| 169 | piR-43770 | DQ575658 | TCCCTGGTGGTCTAGTGGTTAGGATA | 26 |
| 170 | piR-36743 | DQ598677 | GTTTCCGTAGTGTAGTGGTCATCACGTTCGCC | 32 |
| 171 | piR-33519 | DQ593407 | CGTAGTGTAGTGGTCATCACGTTCGCCT | 28 |
| 172 | piR-30840 | DQ570728 | AGAACGTGTGGAAAACTAATGACTGAGC | 28 |
| 173 | piR-57125 | DQ590013 | TGGTCGTGGTTGTAGTCCGTGCGAGAA | 27 |
| 174 | piR-61465 | DQ595353 | TTGGCATCAATATGGTGACCTCTCGGGAGC | 30 |
| 175 | piR-45371 | DQ577259 | TCTACTGAACTGCCATGAGGAAACTG | 26 |
| 176 | piR-33043 | DQ592931 | CCCCCCACTGCTAAATTTGACTGGCTA | 27 |
| 177 | piR-45029 | DQ576917 | TCGCTGGTTCGAATCCGGCTCGGAGGAC | 28 |
| 178 | piR-33748 | DQ593636 | CTCTACTGAACTGCCATGAGGAAACTGCC | 29 |
| 179 | piR-38512 | DQ600446 | TACGTGGACGACACGCAGTTCGTGCGGTTC | 30 |
| 180 | piR-36339 | DQ598273 | GGTCGCTGGTTCGAATCCGGCTCGAAGGACC | 31 |
| 181 | piR-36026 | DQ597960 | GGCCCCATGGTGTAATGGTCAGCACTC | 27 |
| 182 | piR-52016 | DQ584904 | TGCCTAGTGGGCCACTTTTGGTAAGCAGAA | 30 |
| 183 | piR-33415 | DQ593303 | CGCGGGTTCGATCCCCGTACGGGCCACC | 28 |
| 184 | piR-35469 | DQ597403 | GCCTGGGTAGCTCAGTCGGTAGAGCATCAGAC | 32 |
| 185 | piR-33151 | DQ593039 | CCGCCTGGGAATACCGGGTGCTGTAGGCTTA | 31 |
| 186 | piR-44345 | DQ576233 | TCCTCCTACAAAGGCGTGTCTGTGGTTCCC | 30 |
| 187 | piR-36685 | DQ598619 | GTGTAGCTCAGTGGTAGAGCGCGTGCTTCGC | 31 |
| 188 | piR-49124 | DQ581012 | TGAGGGTTCGAGTCCCTTCGTGGTCGCC | 28 |
| 189 | piR-55985 | DQ588873 | TGGGATGAGCCGAGATGGCGCCACTGCA | 28 |
| 190 | piR-31935 | DQ571823 | ATTTGGTGTATGTGCTTGGCTGAGGAGCCAA | 31 |
| 191 | piR-30229 | DQ570117 | AAGCCAGGGATTGTGGGTTCGGGTCCCATCT | 31 |
| 192 | piR-41464 | DQ573352 | TCAGACATTTGGTGTATGTGCTTGGC | 26 |
| 193 | piR-35551 | DQ597485 | GCTAAACCTAGCCCCAAACCCACTCCACCCT | 31 |
| 194 | piR-57516 | DQ590404 | TGGTGTATGTGCTTGGCTGAGGAGCCAATGG | 31 |
| 195 | piR-33536 | DQ593424 | CGTGAGTTCGATCCTCACACGGGGCACCA | 29 |
| 196 | piR-36082 | DQ598016 | GGCTCTGTTGCGCAATGGATAGCGCAT | 27 |
| 197 | piR-51810 | DQ584698 | TGCCCCCATGTCTAACAACATGGCTTTCTCA | 31 |
| 198 | piR-55270 | DQ588158 | TGGCTGAGGCAGGAGGATCACTTGAATC | 28 |
| 199 | piR-55152 | DQ588040 | TGGCGATGAGGAGGTACCTATTGTGTTGAGTA | 32 |
| 200 | piR-55151 | DQ588039 | TGGCGATGAGGAGGTACCTATTGTGTTGAGT | 31 |
| 201 | piR-31106 | DQ570994 | AGCCCTGATGATGCCCACTCCTGAGC | 26 |
| 202 | piR-36170 | DQ598104 | GGGAGATGAAGAGGACAGTGACTGAGAGAC | 30 |
| 203 | piR-35706 | DQ597640 | GCTTGGAGTGACTTCAGCTGCTGAGA | 26 |
| 204 | piR-52729 | DQ585617 | TGCTATCTGAGAGATGGTGATGACATTA | 28 |
| 205 | piR-51309 | DQ584197 | TGCCAATGATGGTTAAGAATTTCTTCACCTGA | 32 |
| 206 | piR-33466 | DQ593354 | CGGCGGTGGCGGCGGCGGCGGCGGGACC | 28 |
| 207 | piR-36707 | DQ598641 | GTTAAGATGGCAGAGCCCGGTAATCGCATAA | 31 |
| 208 | piR-34984 | DQ596918 | GATGTCTGTGTGGAAAGCGGCTGTGCA | 27 |
| 209 | piR-58469 | DQ591357 | TGTGAATCTGACAACAGAGGCTTACGACCCC | 31 |
| 210 | piR-31987 | DQ571875 | CAACAATAGGGTTTACGACCTCGATGTTGGA | 31 |
| 211 | piR-44892 | DQ576780 | TCGAAGGTGGATTTAGCAGTAAACTGA | 27 |
| 212 | piR-34536 | DQ596470 | GAGAAAGCTCACAAGAACTGCTAACTCATGC | 31 |
| 213 | piR-47305 | DQ579193 | TGAATCTGACAACAGAGGCTTACGACCCCTTA | 32 |
| 214 | piR-36056 | DQ597990 | GGCGACAAACCTACCGAGCCTGGTGATAG | 29 |
| 215 | piR-34789 | DQ596723 | GAGGCGGGCATGACACAGCAAGACGAGAAG | 30 |
| 216 | piR-33783 | DQ593671 | CTGAACTCCTCACACCCAATTGGACCA | 27 |
| 217 | piR-44312 | DQ576200 | TCCTCATTAGTATAGTGGTGAGTATCCC | 28 |
| 218 | piR-30625 | DQ570513 | ACCGTCGTAGTTCCGACCATAAACGATGCC | 30 |
| 219 | piR-33082 | DQ592970 | CCCGGCCCGGACACGGACAGGATTGACAGATT | 32 |
| 220 | piR-32678 | DQ582566 | CATTGATCATCGACACTTCGAACGCACTTG | 30 |
| 221 | piR-41209 | DQ573097 | TCACCCGGCCCGGACACGGACAGGATTGACA | 31 |
| 222 | piR-30112 | DQ570000 | AAATGTTATGATGATGGGCGAAATGTTCAACT | 32 |
| 223 | piR-30113 | DQ570001 | AAATGTTGGTTATACCCTTCCCGTACTACC | 30 |
| 224 | piR-54907 | DQ587795 | TGGCCAAGGATGAGAACTCTAATCTGAAA | 29 |
| 225 | piR-60573 | DQ594461 | TTCCGGGTTCGAGTCCCGGCGGAGTCGCC | 29 |
| 226 | piR-30926 | DQ570814 | AGAGATAGCAGAGTGGCGCAGCGGAAGC | 28 |
| 227 | piR-36340 | DQ598274 | GGTCGCTGGTTCGTTTCCGGCTCGAAGGACC | 31 |
| 228 | piR-33880 | DQ593768 | CTGCGTGTTCAAGTCACGTCGGGGTCAC | 28 |
| 229 | piR-53542 | DQ586430 | TGGAAAGGATGAAGAGCTGACTGATGGAA | 29 |
| 230 | piR-34533 | DQ596467 | GAGAAAGCTCACAAGAACTGCTAACTCACC | 30 |
| 231 | piR-33081 | DQ592969 | CCCGCGCAGGTTCGTATCCTGCCGACTACGC | 31 |
| 232 | piR-33382 | DQ593270 | CGCACGTGTTAGGACCCGAAAGATGGTGAAC | 31 |
| 233 | piR-33387 | DQ593275 | CGCAGAGTCGCGCAGCGGAAGCGTGCTGGGCC | 32 |
| 234 | piR-56450 | DQ589338 | TGGGGGGGCCCAAGTCCTTCTGATCGAGG | 29 |
