# Supplementary material for: Genome-wide profiling of the PIWI-interacting RNA-mRNA regulatory networks in epithelial ovarian cancers
Source: PLoS One. 2018 Jan 10;13(1):e0190485. doi: 10.1371/journal.pone.0190485 (PMC5761873; doi:10.1371/journal.pone.0190485)
Supplement: S2 Table — (DOCX) [file pone.0190485.s002.docx]

Table S2A. Number of unique reads and read count of miRNAs detected in Normal ovary

| **S. N.** | **miRNA** | **Unique Reads** | **Read Count** |
| --- | --- | --- | --- |
| 1 | hsa-miR-10b-5p | 1470 | 1027911 |
| 2 | hsa-miR-26a-5p | 476 | 292954 |
| 3 | hsa-miR-3182 | 79 | 189280 |
| 4 | hsa-miR-143-3p | 566 | 176878 |
| 5 | hsa-let-7a-5p | 195 | 106444 |
| 6 | hsa-miR-92a-3p | 524 | 102646 |
| 7 | hsa-miR-125b-5p | 351 | 60084 |
| 8 | hsa-miR-125a-5p | 248 | 42629 |
| 9 | hsa-let-7b-5p | 291 | 37112 |
| 10 | hsa-miR-148a-3p | 203 | 34627 |
| 11 | hsa-let-7c-5p | 139 | 32561 |
| 12 | hsa-miR-181a-5p | 166 | 27549 |
| 13 | hsa-miR-27b-3p | 140 | 26073 |
| 14 | hsa-let-7f-5p | 126 | 23104 |
| 15 | hsa-miR-1273g-3p | 94 | 19484 |
| 16 | hsa-miR-22-3p | 82 | 17499 |
| 17 | hsa-miR-191-5p | 111 | 16582 |
| 18 | hsa-miR-100-5p | 168 | 14883 |
| 19 | hsa-miR-146b-5p | 98 | 12444 |
| 20 | hsa-miR-30d-5p | 131 | 12311 |
| 21 | hsa-miR-101-3p | 133 | 12089 |
| 22 | hsa-miR-29a-3p | 143 | 10304 |
| 23 | hsa-miR-186-5p | 94 | 10169 |
| 24 | hsa-miR-28-3p | 83 | 9459 |
| 25 | hsa-let-7i-5p | 77 | 9387 |
| 26 | hsa-miR-26b-5p | 104 | 9003 |
| 27 | hsa-let-7g-5p | 73 | 8663 |
| 28 | hsa-miR-99a-5p | 99 | 7701 |
| 29 | hsa-miR-21-5p | 85 | 7676 |
| 30 | hsa-miR-4448 | 13 | 7063 |
| 31 | hsa-miR-30e-5p | 88 | 6917 |
| 32 | hsa-miR-145-5p | 92 | 6674 |
| 33 | hsa-miR-151a-3p | 119 | 6490 |
| 34 | hsa-miR-199a-5p | 101 | 6444 |
| 35 | hsa-miR-99b-5p | 99 | 6433 |
| 36 | hsa-miR-204-5p | 55 | 5674 |
| 37 | hsa-miR-10a-5p | 85 | 5666 |
| 38 | hsa-miR-30a-5p | 63 | 5621 |
| 39 | hsa-miR-16-5p | 53 | 5016 |
| 40 | hsa-miR-125b-2-3p | 85 | 4251 |
| 41 | hsa-miR-195-5p | 52 | 4246 |
| 42 | hsa-miR-151a-5p | 43 | 4244 |
| 43 | hsa-miR-30c-5p | 54 | 4114 |
| 44 | hsa-miR-140-3p | 110 | 4041 |
| 45 | hsa-miR-320a | 97 | 3940 |
| 46 | hsa-miR-130a-3p | 57 | 3712 |
| 47 | hsa-miR-424-5p | 57 | 3543 |
| 48 | hsa-miR-19b-3p | 42 | 3158 |
| 49 | hsa-miR-532-5p | 64 | 3089 |
| 50 | hsa-miR-92b-3p | 115 | 2995 |
| 51 | hsa-miR-23b-3p | 59 | 2992 |
| 52 | hsa-miR-423-3p | 58 | 2881 |
| 53 | hsa-miR-486-5p | 45 | 2846 |
| 54 | hsa-let-7e-5p | 41 | 2845 |
| 55 | hsa-miR-103a-3p | 26 | 2528 |
| 56 | hsa-miR-181b-5p | 63 | 2413 |
| 57 | hsa-miR-30b-5p | 35 | 2395 |
| 58 | hsa-miR-381-3p | 33 | 2359 |
| 59 | hsa-miR-509-3p | 44 | 2320 |
| 60 | hsa-miR-25-3p | 47 | 2233 |
| 61 | hsa-miR-29c-3p | 53 | 1930 |
| 62 | hsa-miR-221-3p | 45 | 1835 |
| 63 | hsa-miR-378a-3p | 28 | 1733 |
| 64 | hsa-miR-5684 | 7 | 1671 |
| 65 | hsa-miR-660-5p | 35 | 1641 |
| 66 | hsa-miR-181c-5p | 30 | 1582 |
| 67 | hsa-let-7d-5p | 29 | 1575 |
| 68 | hsa-miR-192-5p | 39 | 1530 |
| 69 | hsa-miR-222-3p | 30 | 1330 |
| 70 | hsa-miR-500a-3p | 17 | 1246 |
| 71 | hsa-miR-126-5p | 16 | 1233 |
| 72 | hsa-miR-27a-3p | 34 | 1190 |
| 73 | hsa-miR-708-5p | 22 | 1020 |
| 74 | hsa-miR-98-5p | 18 | 1012 |
| 75 | hsa-miR-126-3p | 28 | 993 |
| 76 | hsa-miR-23a-3p | 26 | 987 |
| 77 | hsa-miR-423-5p | 27 | 948 |
| 78 | hsa-miR-7641 | 8 | 946 |
| 79 | hsa-miR-451a | 17 | 909 |
| 80 | hsa-miR-574-3p | 29 | 908 |
| 81 | hsa-miR-24-3p | 23 | 884 |
| 82 | hsa-miR-769-5p | 15 | 883 |
| 83 | hsa-miR-30e-3p | 19 | 839 |
| 84 | hsa-miR-152-3p | 25 | 838 |
| 85 | hsa-miR-508-3p | 21 | 821 |
| 86 | hsa-miR-497-5p | 23 | 801 |
| 87 | hsa-miR-424-3p | 26 | 797 |
| 88 | hsa-miR-196a-5p | 16 | 772 |
| 89 | hsa-miR-411-5p | 16 | 731 |
| 90 | hsa-miR-136-3p | 12 | 702 |
| 91 | hsa-miR-134-5p | 28 | 692 |
| 92 | hsa-miR-145-3p | 34 | 618 |
| 93 | hsa-miR-197-3p | 24 | 617 |
| 94 | hsa-miR-125b-1-3p | 14 | 604 |
| 95 | hsa-miR-4792 | 9 | 599 |
| 96 | hsa-miR-654-3p | 27 | 587 |
| 97 | hsa-miR-342-3p | 22 | 529 |
| 98 | hsa-miR-6131 | 5 | 526 |
| 99 | hsa-miR-28-5p | 13 | 506 |
| 100 | hsa-miR-193b-3p | 24 | 492 |
| 101 | hsa-miR-214-5p | 14 | 484 |
| 102 | hsa-miR-361-5p | 13 | 465 |
| 103 | hsa-miR-214-3p | 27 | 443 |
| 104 | hsa-miR-502-3p | 15 | 429 |
| 105 | hsa-miR-202-5p | 10 | 427 |
| 106 | hsa-miR-34a-5p | 16 | 396 |
| 107 | hsa-miR-361-3p | 16 | 395 |
| 108 | hsa-miR-148b-3p | 10 | 380 |
| 109 | hsa-miR-99a-3p | 12 | 379 |
| 110 | hsa-miR-148a-5p | 9 | 366 |
| 111 | hsa-miR-345-5p | 15 | 340 |
| 112 | hsa-miR-93-5p | 12 | 339 |
| 113 | hsa-miR-335-5p | 6 | 320 |
| 114 | hsa-let-7b-3p | 12 | 318 |
| 115 | hsa-miR-127-3p | 42 | 316 |
| 116 | hsa-miR-4301 | 9 | 305 |
| 117 | hsa-miR-19a-3p | 4 | 281 |
| 118 | hsa-miR-181a-2-3p | 10 | 278 |
| 119 | hsa-miR-4508 | 2 | 275 |
| 120 | hsa-miR-339-3p | 15 | 270 |
| 121 | hsa-miR-146b-3p | 13 | 254 |
| 122 | hsa-let-7d-3p | 10 | 249 |
| 123 | hsa-miR-664a-3p | 11 | 228 |
| 124 | hsa-miR-328-3p | 14 | 227 |
| 125 | hsa-miR-155-5p | 11 | 226 |
| 126 | hsa-miR-1202 | 1 | 210 |
| 127 | hsa-miR-887-3p | 10 | 200 |
| 128 | hsa-miR-15b-5p | 6 | 198 |
| 129 | hsa-miR-203a-3p | 10 | 197 |
| 130 | hsa-miR-146a-5p | 12 | 197 |
| 131 | hsa-miR-941 | 8 | 196 |
| 132 | hsa-miR-132-3p | 9 | 194 |
| 133 | hsa-miR-4497 | 5 | 192 |
| 134 | hsa-miR-574-5p | 15 | 187 |
| 135 | hsa-miR-340-5p | 3 | 180 |
| 136 | hsa-miR-410-3p | 5 | 180 |
| 137 | hsa-miR-196b-5p | 7 | 175 |
| 138 | hsa-miR-450a-5p | 10 | 171 |
| 139 | hsa-miR-1468-5p | 7 | 169 |
| 140 | hsa-miR-182-5p | 7 | 166 |
| 141 | hsa-miR-708-3p | 9 | 166 |
| 142 | hsa-miR-20a-5p | 6 | 165 |
| 143 | hsa-miR-455-5p | 6 | 161 |
| 144 | hsa-miR-181d-5p | 7 | 160 |
| 145 | hsa-miR-484 | 10 | 160 |
| 146 | hsa-miR-128-3p | 11 | 160 |
| 147 | hsa-miR-374b-5p | 8 | 160 |
| 148 | hsa-miR-150-5p | 5 | 159 |
| 149 | hsa-miR-532-3p | 7 | 155 |
| 150 | hsa-miR-21-3p | 7 | 153 |
| 151 | hsa-miR-195-3p | 9 | 153 |
| 152 | hsa-miR-4532 | 7 | 150 |
| 153 | hsa-miR-450b-5p | 5 | 149 |
| 154 | hsa-miR-425-5p | 6 | 148 |
| 155 | hsa-miR-432-5p | 13 | 144 |
| 156 | hsa-miR-331-3p | 9 | 143 |
| 157 | hsa-miR-4492 | 1 | 140 |
| 158 | hsa-miR-1247-5p | 8 | 136 |
| 159 | hsa-miR-136-5p | 3 | 135 |
| 160 | hsa-miR-409-3p | 7 | 135 |
| 161 | hsa-miR-874-3p | 9 | 135 |
| 162 | hsa-miR-29b-3p | 11 | 135 |
| 163 | hsa-miR-301a-3p | 3 | 133 |
| 164 | hsa-miR-3609 | 2 | 133 |
| 165 | hsa-miR-1246 | 5 | 132 |
| 166 | hsa-miR-501-3p | 8 | 130 |
| 167 | hsa-miR-17-5p | 6 | 128 |
| 168 | hsa-miR-744-5p | 8 | 122 |
| 169 | hsa-miR-3960 | 14 | 118 |
| 170 | hsa-miR-493-5p | 4 | 117 |
| 171 | hsa-miR-652-3p | 9 | 115 |
| 172 | hsa-miR-509-3-5p | 6 | 114 |
| 173 | hsa-miR-30a-3p | 10 | 113 |
| 174 | hsa-miR-493-3p | 6 | 110 |
| 175 | hsa-miR-542-3p | 6 | 110 |
| 176 | hsa-miR-374a-5p | 4 | 107 |
| 177 | hsa-miR-199b-5p | 6 | 104 |
| 178 | hsa-miR-376c-3p | 8 | 102 |
| 179 | hsa-miR-15a-5p | 5 | 102 |
| 180 | hsa-miR-421 | 8 | 101 |
| 181 | hsa-miR-4454 | 7 | 98 |
| 182 | hsa-miR-210-3p | 8 | 94 |
| 183 | hsa-miR-149-5p | 7 | 92 |
| 184 | hsa-miR-362-5p | 8 | 91 |
| 185 | hsa-miR-455-3p | 6 | 90 |
| 186 | hsa-miR-383-5p | 4 | 89 |
| 187 | hsa-miR-513c-5p | 5 | 83 |
| 188 | hsa-miR-3607-3p | 3 | 79 |
| 189 | hsa-miR-193a-5p | 6 | 77 |
| 190 | hsa-miR-106b-3p | 7 | 76 |
| 191 | hsa-miR-140-5p | 6 | 75 |
| 192 | hsa-miR-487b-3p | 7 | 75 |
| 193 | hsa-miR-505-3p | 8 | 73 |
| 194 | hsa-miR-127-5p | 8 | 71 |
| 195 | hsa-miR-194-5p | 3 | 71 |
| 196 | hsa-miR-1271-5p | 4 | 71 |
| 197 | hsa-miR-374a-3p | 1 | 69 |
| 198 | hsa-miR-1296-5p | 4 | 67 |
| 199 | hsa-miR-221-5p | 5 | 66 |
| 200 | hsa-miR-339-5p | 6 | 66 |
| 201 | hsa-miR-181c-3p | 5 | 65 |
| 202 | hsa-miR-143-5p | 5 | 65 |
| 203 | hsa-miR-141-3p | 1 | 64 |
| 204 | hsa-miR-299-3p | 3 | 63 |
| 205 | hsa-miR-363-3p | 5 | 63 |
| 206 | hsa-miR-4791 | 1 | 63 |
| 207 | hsa-miR-615-3p | 7 | 63 |
| 208 | hsa-miR-29c-5p | 9 | 63 |
| 209 | hsa-miR-5701 | 1 | 63 |
| 210 | hsa-miR-500a-5p | 10 | 61 |
| 211 | hsa-miR-454-3p | 2 | 60 |
| 212 | hsa-miR-335-3p | 3 | 60 |
| 213 | hsa-miR-376a-3p | 4 | 58 |
| 214 | hsa-miR-193a-3p | 5 | 56 |
| 215 | hsa-miR-200b-3p | 5 | 55 |
| 216 | hsa-miR-99b-3p | 6 | 53 |
| 217 | hsa-miR-31-5p | 4 | 51 |
| 218 | hsa-miR-3168 | 9 | 50 |
| 219 | hsa-miR-379-5p | 5 | 49 |
| 220 | hsa-miR-106b-5p | 5 | 49 |
| 221 | hsa-let-7i-3p | 4 | 48 |
| 222 | hsa-miR-10b-3p | 6 | 47 |
| 223 | hsa-miR-154-5p | 3 | 47 |
| 224 | hsa-miR-495-3p | 4 | 46 |
| 225 | hsa-miR-514a-3p | 4 | 46 |
| 226 | hsa-miR-27b-5p | 2 | 46 |
| 227 | hsa-miR-1248 | 1 | 45 |
| 228 | hsa-miR-542-5p | 3 | 45 |
| 229 | hsa-miR-133a-3p | 3 | 43 |
| 230 | hsa-miR-561-5p | 1 | 43 |
| 231 | hsa-miR-3195 | 6 | 40 |
| 232 | hsa-miR-26b-3p | 4 | 40 |
| 233 | hsa-miR-589-5p | 2 | 38 |
| 234 | hsa-miR-1247-3p | 3 | 38 |
| 235 | hsa-miR-369-5p | 2 | 37 |
| 236 | hsa-miR-7704 | 6 | 37 |
| 237 | hsa-miR-107 | 3 | 37 |
| 238 | hsa-miR-30d-3p | 5 | 36 |
| 239 | hsa-miR-377-5p | 2 | 36 |
| 240 | hsa-miR-324-5p | 3 | 35 |
| 241 | hsa-miR-223-3p | 5 | 35 |
| 242 | hsa-miR-125a-3p | 3 | 34 |
| 243 | hsa-let-7a-3p | 4 | 34 |
| 244 | hsa-miR-485-5p | 2 | 33 |
| 245 | hsa-miR-17-3p | 3 | 32 |
| 246 | hsa-miR-503-5p | 5 | 31 |
| 247 | hsa-miR-100-3p | 1 | 31 |
| 248 | hsa-miR-874-5p | 3 | 31 |
| 249 | hsa-miR-27a-5p | 2 | 31 |
| 250 | hsa-miR-200c-3p | 3 | 31 |
| 251 | hsa-miR-139-5p | 3 | 30 |
| 252 | hsa-miR-3196 | 2 | 30 |
| 253 | hsa-miR-576-5p | 3 | 29 |
| 254 | hsa-miR-202-3p | 3 | 29 |
| 255 | hsa-miR-671-3p | 2 | 28 |
| 256 | hsa-miR-1307-3p | 5 | 28 |
| 257 | hsa-miR-337-3p | 4 | 26 |
| 258 | hsa-miR-4510 | 4 | 26 |
| 259 | hsa-let-7e-3p | 4 | 26 |
| 260 | hsa-miR-320b | 6 | 26 |
| 261 | hsa-miR-9-5p | 2 | 25 |
| 262 | hsa-miR-382-5p | 3 | 25 |
| 263 | hsa-miR-508-5p | 5 | 24 |
| 264 | hsa-miR-129-2-3p | 3 | 24 |
| 265 | hsa-miR-5100 | 6 | 24 |
| 266 | hsa-miR-30c-1-3p | 3 | 24 |
| 267 | hsa-miR-4516 | 4 | 23 |
| 268 | hsa-miR-32-5p | 2 | 23 |
| 269 | hsa-let-7f-2-3p | 1 | 23 |
| 270 | hsa-miR-142-5p | 1 | 23 |
| 271 | hsa-miR-1180-3p | 2 | 23 |
| 272 | hsa-miR-758-3p | 3 | 23 |
| 273 | hsa-miR-3607-5p | 1 | 23 |
| 274 | hsa-miR-1275 | 2 | 22 |
| 275 | hsa-miR-494-3p | 3 | 21 |
| 276 | hsa-miR-135a-5p | 2 | 21 |
| 277 | hsa-miR-130b-3p | 5 | 21 |
| 278 | hsa-miR-377-3p | 2 | 21 |
| 279 | hsa-miR-641 | 2 | 21 |
| 280 | hsa-miR-485-3p | 3 | 20 |
| 281 | hsa-miR-7977 | 3 | 20 |
| 282 | hsa-miR-889-3p | 3 | 20 |
| 283 | hsa-miR-215-5p | 3 | 20 |
| 284 | hsa-let-7g-3p | 3 | 19 |
| 285 | hsa-miR-452-5p | 4 | 19 |
| 286 | hsa-miR-370-3p | 3 | 18 |
| 287 | hsa-let-7c-3p | 2 | 18 |
| 288 | hsa-miR-205-5p | 2 | 17 |
| 289 | hsa-miR-181a-3p | 1 | 17 |
| 290 | hsa-miR-299-5p | 2 | 16 |
| 291 | hsa-miR-378a-5p | 1 | 16 |
| 292 | hsa-miR-326 | 3 | 15 |
| 293 | hsa-miR-144-5p | 2 | 15 |
| 294 | hsa-miR-625-5p | 2 | 14 |
| 295 | hsa-miR-376a-5p | 2 | 14 |
| 296 | hsa-miR-4787-5p | 1 | 14 |
| 297 | hsa-miR-501-5p | 3 | 14 |
| 298 | hsa-miR-92a-1-5p | 1 | 13 |
| 299 | hsa-miR-378i | 2 | 12 |
| 300 | hsa-miR-379-3p | 1 | 12 |
| 301 | hsa-miR-655-3p | 2 | 12 |
| 302 | hsa-miR-433-3p | 1 | 12 |
| 303 | hsa-miR-3653-5p | 1 | 12 |
| 304 | hsa-miR-4662a-5p | 1 | 12 |
| 305 | hsa-miR-548o-3p | 1 | 12 |
| 306 | hsa-miR-101-5p | 3 | 11 |
| 307 | hsa-miR-629-5p | 2 | 11 |
| 308 | hsa-miR-7-1-3p | 2 | 11 |
| 309 | hsa-miR-411-3p | 1 | 10 |
| 310 | hsa-miR-152-5p | 1 | 10 |
| 311 | hsa-miR-1307-5p | 1 | 10 |
| 312 | hsa-miR-302a-5p | 2 | 10 |
| 313 | hsa-miR-362-3p | 1 | 9 |
| 314 | hsa-miR-539-3p | 2 | 9 |
| 315 | hsa-miR-7706 | 2 | 9 |
| 316 | hsa-miR-548k | 1 | 9 |
| 317 | hsa-miR-590-3p | 2 | 9 |
| 318 | hsa-miR-660-3p | 2 | 9 |
| 319 | hsa-miR-200a-3p | 3 | 9 |
| 320 | hsa-miR-153-3p | 1 | 9 |
| 321 | hsa-miR-1301-3p | 2 | 9 |
| 322 | hsa-miR-4677-3p | 1 | 8 |
| 323 | hsa-miR-378c | 2 | 8 |
| 324 | hsa-miR-2114-5p | 1 | 8 |
| 325 | hsa-miR-548i | 2 | 8 |
| 326 | hsa-miR-509-5p | 2 | 8 |
| 327 | hsa-miR-625-3p | 2 | 8 |
| 328 | hsa-miR-25-5p | 1 | 8 |
| 329 | hsa-miR-499a-5p | 1 | 7 |
| 330 | hsa-miR-20a-3p | 2 | 7 |
| 331 | hsa-miR-543 | 1 | 7 |
| 332 | hsa-miR-369-3p | 2 | 7 |
| 333 | hsa-let-7f-1-3p | 1 | 7 |
| 334 | hsa-miR-671-5p | 1 | 7 |
| 335 | hsa-miR-29a-5p | 2 | 7 |
| 336 | hsa-miR-375 | 1 | 7 |
| 337 | hsa-miR-185-5p | 2 | 7 |
| 338 | hsa-miR-320d | 2 | 7 |
| 339 | hsa-miR-330-5p | 2 | 7 |
| 340 | hsa-miR-34c-5p | 1 | 7 |
| 341 | hsa-miR-382-3p | 1 | 7 |
| 342 | hsa-miR-302a-3p | 1 | 6 |
| 343 | hsa-miR-188-5p | 1 | 6 |
| 344 | hsa-miR-342-5p | 2 | 6 |
| 345 | hsa-miR-1185-5p | 2 | 6 |
| 346 | hsa-miR-93-3p | 1 | 6 |
| 347 | hsa-miR-301b-3p | 1 | 6 |
| 348 | hsa-miR-487a-5p | 1 | 6 |
| 349 | hsa-miR-1287-5p | 1 | 6 |
| 350 | hsa-miR-6511b-3p | 2 | 6 |
| 351 | hsa-miR-487a-3p | 1 | 6 |
| 352 | hsa-miR-429 | 1 | 6 |
| 353 | hsa-miR-30c-2-3p | 1 | 6 |
| 354 | hsa-miR-4286 | 2 | 6 |
| 355 | hsa-miR-129-5p | 2 | 6 |
| 356 | hsa-miR-324-3p | 1 | 6 |
| 357 | hsa-miR-3615 | 1 | 6 |
| 358 | hsa-miR-219a-1-3p | 1 | 6 |
| 359 | hsa-miR-4485-3p | 2 | 6 |
| 360 | hsa-miR-483-3p | 2 | 6 |
| 361 | hsa-miR-548ba | 1 | 6 |
| 362 | hsa-miR-3909 | 1 | 6 |
| 363 | hsa-miR-26a-2-3p | 1 | 6 |
| 364 | hsa-miR-675-3p | 1 | 5 |
| 365 | hsa-miR-135a-3p | 1 | 5 |
| 366 | hsa-miR-3605-3p | 1 | 5 |
| 367 | hsa-miR-664a-5p | 1 | 5 |
| 368 | hsa-miR-1304-3p | 1 | 5 |
| 369 | hsa-miR-22-5p | 1 | 5 |
| 370 | hsa-miR-212-5p | 1 | 5 |
| 371 | hsa-miR-132-5p | 1 | 5 |
| 372 | hsa-miR-302d-3p | 1 | 5 |
| 373 | hsa-miR-598-3p | 1 | 5 |
| 374 | hsa-miR-212-3p | 1 | 5 |
| 375 | hsa-miR-190a-5p | 1 | 5 |
| 376 | hsa-miR-18a-5p | 1 | 5 |
| 377 | hsa-miR-224-5p | 1 | 5 |
| 378 | hsa-miR-29b-2-5p | 1 | 5 |
| 379 | hsa-miR-340-3p | 1 | 5 |
| 380 | hsa-miR-374b-3p | 1 | 4 |
| 381 | hsa-miR-1290 | 1 | 4 |
| 382 | hsa-miR-3158-3p | 1 | 4 |
| 383 | hsa-miR-4461 | 1 | 4 |
| 384 | hsa-miR-513a-5p | 1 | 4 |
| 385 | hsa-miR-338-3p | 1 | 4 |
| 386 | hsa-miR-193b-5p | 1 | 4 |
| 387 | hsa-miR-296-5p | 1 | 4 |
| 388 | hsa-miR-4284 | 1 | 4 |
| 389 | hsa-miR-323a-3p | 1 | 4 |
| 390 | hsa-miR-2355-5p | 1 | 4 |
| 391 | hsa-miR-628-5p | 1 | 4 |
| 392 | hsa-miR-134-3p | 1 | 4 |
| 393 | hsa-miR-372-3p | 1 | 3 |
| 394 | hsa-miR-877-5p | 1 | 3 |
| 395 | hsa-miR-4531 | 1 | 3 |
| 396 | hsa-miR-770-5p | 1 | 3 |
| 397 | hsa-miR-378d | 1 | 3 |
| 398 | hsa-miR-548e-3p | 1 | 3 |
| 399 | hsa-miR-106a-5p | 1 | 3 |
| 400 | hsa-miR-624-5p | 1 | 3 |
| 401 | hsa-miR-210-5p | 1 | 3 |
| 402 | hsa-miR-2114-3p | 1 | 3 |
| 403 | hsa-miR-5096 | 1 | 3 |
| 404 | hsa-miR-507 | 1 | 3 |
| 405 | hsa-miR-183-5p | 1 | 3 |
| 406 | hsa-miR-19b-1-5p | 1 | 3 |
| 407 | hsa-miR-576-3p | 1 | 3 |
| 408 | hsa-miR-513b-5p | 1 | 3 |
| 409 | hsa-miR-6087 | 1 | 3 |
| 410 | hsa-miR-3687 | 1 | 3 |
| 411 | hsa-miR-218-5p | 1 | 3 |
| 412 | hsa-miR-1260b | 1 | 3 |
| 413 | hsa-miR-329-3p | 1 | 3 |
| 414 | hsa-miR-142-3p | 1 | 3 |
| 415 | hsa-miR-548h-5p | 1 | 3 |
| 416 | hsa-miR-582-5p | 1 | 3 |
| 417 | hsa-miR-510-5p | 1 | 3 |
| 418 | hsa-miR-2467-5p | 1 | 3 |
| 419 | hsa-miR-23a-5p | 1 | 3 |
| 420 | hsa-miR-5000-3p | 1 | 3 |
| 421 | hsa-miR-584-5p | 1 | 3 |
| 422 | hsa-miR-184 | 1 | 3 |
| 423 | hsa-miR-618 | 1 | 3 |
| 424 | hsa-miR-431-3p | 1 | 3 |

Table S2B. Number of unique reads and read count of miRNAs detected in ENOCa

| **S. N.** | **miRNA** | **unique Reads** | **Read Count** |
| --- | --- | --- | --- |
| 1 | hsa-miR-10a-5p | 912 | 414328 |
| 2 | hsa-miR-10b-5p | 435 | 106863 |
| 3 | hsa-miR-3182 | 57 | 82116 |
| 4 | hsa-let-7a-5p | 121 | 64916 |
| 5 | hsa-miR-92a-3p | 416 | 59213 |
| 6 | hsa-miR-26a-5p | 156 | 45235 |
| 7 | hsa-miR-27b-3p | 160 | 42540 |
| 8 | hsa-miR-181a-5p | 188 | 41580 |
| 9 | hsa-miR-21-5p | 162 | 32216 |
| 10 | hsa-miR-191-5p | 142 | 30278 |
| 11 | hsa-miR-30e-5p | 135 | 23620 |
| 12 | hsa-miR-182-5p | 149 | 20356 |
| 13 | hsa-miR-1273g-3p | 92 | 19997 |
| 14 | hsa-miR-22-3p | 83 | 18529 |
| 15 | hsa-let-7f-5p | 79 | 17905 |
| 16 | hsa-miR-378a-3p | 111 | 17555 |
| 17 | hsa-miR-143-3p | 132 | 16048 |
| 18 | hsa-let-7b-5p | 149 | 15672 |
| 19 | hsa-miR-141-3p | 63 | 13418 |
| 20 | hsa-miR-30c-5p | 87 | 12862 |
| 21 | hsa-miR-186-5p | 101 | 12141 |
| 22 | hsa-miR-92b-3p | 251 | 11790 |
| 23 | hsa-miR-148a-3p | 90 | 11739 |
| 24 | hsa-miR-125a-5p | 121 | 11276 |
| 25 | hsa-miR-30d-5p | 103 | 11258 |
| 26 | hsa-miR-16-5p | 60 | 10513 |
| 27 | hsa-miR-146b-5p | 90 | 10100 |
| 28 | hsa-miR-151a-5p | 59 | 9150 |
| 29 | hsa-miR-151a-3p | 116 | 8743 |
| 30 | hsa-miR-103a-3p | 59 | 8040 |
| 31 | hsa-miR-200b-3p | 92 | 7736 |
| 32 | hsa-miR-200c-3p | 76 | 7306 |
| 33 | hsa-miR-29a-3p | 90 | 6699 |
| 34 | hsa-miR-28-3p | 58 | 6224 |
| 35 | hsa-miR-4448 | 22 | 5925 |
| 36 | hsa-miR-25-3p | 61 | 5646 |
| 37 | hsa-miR-26b-5p | 55 | 4626 |
| 38 | hsa-miR-130a-3p | 51 | 4486 |
| 39 | hsa-let-7i-5p | 43 | 4426 |
| 40 | hsa-miR-99b-5p | 69 | 4372 |
| 41 | hsa-let-7g-5p | 33 | 4266 |
| 42 | hsa-miR-181b-5p | 74 | 4131 |
| 43 | hsa-let-7d-5p | 39 | 4014 |
| 44 | hsa-miR-23b-3p | 69 | 3982 |
| 45 | hsa-miR-93-5p | 68 | 3952 |
| 46 | hsa-miR-486-5p | 61 | 3808 |
| 47 | hsa-let-7e-5p | 41 | 3396 |
| 48 | hsa-miR-27a-3p | 36 | 3110 |
| 49 | hsa-miR-30a-5p | 39 | 2748 |
| 50 | hsa-miR-222-3p | 43 | 2640 |
| 51 | hsa-miR-30e-3p | 35 | 2603 |
| 52 | hsa-miR-221-3p | 44 | 2437 |
| 53 | hsa-miR-205-5p | 41 | 2322 |
| 54 | hsa-miR-423-3p | 48 | 1916 |
| 55 | hsa-miR-21-3p | 31 | 1801 |
| 56 | hsa-miR-23a-3p | 36 | 1617 |
| 57 | hsa-miR-7641 | 16 | 1549 |
| 58 | hsa-miR-148b-3p | 29 | 1485 |
| 59 | hsa-miR-320a | 47 | 1478 |
| 60 | hsa-miR-192-5p | 35 | 1410 |
| 61 | hsa-miR-769-5p | 23 | 1332 |
| 62 | hsa-miR-24-3p | 27 | 1308 |
| 63 | hsa-miR-335-3p | 24 | 1285 |
| 64 | hsa-miR-4792 | 18 | 1279 |
| 65 | hsa-miR-181c-5p | 20 | 1152 |
| 66 | hsa-miR-941 | 23 | 1115 |
| 67 | hsa-miR-335-5p | 15 | 1085 |
| 68 | hsa-miR-425-5p | 32 | 1069 |
| 69 | hsa-miR-98-5p | 18 | 1018 |
| 70 | hsa-miR-195-5p | 18 | 987 |
| 71 | hsa-miR-183-5p | 27 | 972 |
| 72 | hsa-miR-484 | 35 | 961 |
| 73 | hsa-miR-146a-5p | 29 | 955 |
| 74 | hsa-miR-200a-3p | 22 | 918 |
| 75 | hsa-miR-30b-5p | 27 | 915 |
| 76 | hsa-miR-197-3p | 28 | 911 |
| 77 | hsa-miR-423-5p | 26 | 896 |
| 78 | hsa-miR-451a | 23 | 855 |
| 79 | hsa-miR-128-3p | 34 | 812 |
| 80 | hsa-miR-101-3p | 18 | 796 |
| 81 | hsa-miR-345-5p | 23 | 792 |
| 82 | hsa-miR-126-5p | 15 | 786 |
| 83 | hsa-miR-497-5p | 17 | 648 |
| 84 | hsa-miR-193b-3p | 29 | 641 |
| 85 | hsa-miR-19b-3p | 10 | 638 |
| 86 | hsa-miR-181a-2-3p | 18 | 616 |
| 87 | hsa-miR-140-3p | 27 | 603 |
| 88 | hsa-miR-155-5p | 20 | 585 |
| 89 | hsa-miR-5684 | 5 | 582 |
| 90 | hsa-miR-4454 | 16 | 578 |
| 91 | hsa-let-7d-3p | 15 | 564 |
| 92 | hsa-miR-28-5p | 13 | 552 |
| 93 | hsa-miR-126-3p | 18 | 532 |
| 94 | hsa-miR-421 | 19 | 529 |
| 95 | hsa-miR-148a-5p | 11 | 513 |
| 96 | hsa-miR-500a-3p | 10 | 511 |
| 97 | hsa-miR-361-5p | 14 | 508 |
| 98 | hsa-miR-4508 | 5 | 502 |
| 99 | hsa-miR-429 | 15 | 483 |
| 100 | hsa-miR-15a-5p | 13 | 468 |
| 101 | hsa-miR-363-3p | 15 | 458 |
| 102 | hsa-miR-17-5p | 11 | 443 |
| 103 | hsa-miR-203a-3p | 15 | 435 |
| 104 | hsa-miR-532-5p | 17 | 416 |
| 105 | hsa-miR-301a-3p | 6 | 415 |
| 106 | hsa-miR-361-3p | 17 | 409 |
| 107 | hsa-miR-342-3p | 15 | 408 |
| 108 | hsa-miR-744-5p | 13 | 399 |
| 109 | hsa-miR-34a-5p | 18 | 394 |
| 110 | hsa-miR-130b-3p | 13 | 390 |
| 111 | hsa-miR-100-5p | 10 | 387 |
| 112 | hsa-miR-4301 | 16 | 386 |
| 113 | hsa-miR-454-3p | 10 | 369 |
| 114 | hsa-miR-1246 | 18 | 357 |
| 115 | hsa-miR-20a-5p | 11 | 343 |
| 116 | hsa-miR-15b-5p | 6 | 341 |
| 117 | hsa-miR-141-5p | 10 | 313 |
| 118 | hsa-miR-4497 | 7 | 311 |
| 119 | hsa-miR-574-3p | 13 | 303 |
| 120 | hsa-let-7b-3p | 12 | 294 |
| 121 | hsa-miR-339-3p | 16 | 290 |
| 122 | hsa-miR-142-5p | 10 | 287 |
| 123 | hsa-miR-1307-3p | 14 | 287 |
| 124 | hsa-miR-150-5p | 10 | 277 |
| 125 | hsa-miR-4532 | 10 | 273 |
| 126 | hsa-miR-125b-5p | 10 | 266 |
| 127 | hsa-miR-10a-3p | 12 | 257 |
| 128 | hsa-miR-106b-5p | 6 | 248 |
| 129 | hsa-miR-106b-3p | 10 | 245 |
| 130 | hsa-miR-146b-3p | 12 | 238 |
| 131 | hsa-miR-664a-3p | 10 | 236 |
| 132 | hsa-miR-29c-3p | 15 | 228 |
| 133 | hsa-miR-340-5p | 5 | 225 |
| 134 | hsa-miR-224-5p | 7 | 222 |
| 135 | hsa-miR-1307-5p | 10 | 221 |
| 136 | hsa-miR-3960 | 37 | 212 |
| 137 | hsa-let-7c-5p | 4 | 206 |
| 138 | hsa-miR-3195 | 9 | 205 |
| 139 | hsa-miR-27b-5p | 5 | 201 |
| 140 | hsa-miR-574-5p | 16 | 201 |
| 141 | hsa-miR-7704 | 13 | 198 |
| 142 | hsa-miR-4492 | 5 | 195 |
| 143 | hsa-miR-181a-3p | 7 | 193 |
| 144 | hsa-miR-199a-5p | 8 | 186 |
| 145 | hsa-miR-331-3p | 8 | 183 |
| 146 | hsa-miR-31-5p | 5 | 183 |
| 147 | hsa-miR-145-5p | 8 | 172 |
| 148 | hsa-miR-107 | 5 | 171 |
| 149 | hsa-miR-193a-5p | 8 | 165 |
| 150 | hsa-miR-99b-3p | 10 | 162 |
| 151 | hsa-miR-5701 | 3 | 162 |
| 152 | hsa-miR-132-3p | 5 | 161 |
| 153 | hsa-miR-660-5p | 8 | 160 |
| 154 | hsa-miR-7977 | 5 | 159 |
| 155 | hsa-miR-6131 | 3 | 156 |
| 156 | hsa-miR-181d-5p | 9 | 151 |
| 157 | hsa-miR-5100 | 13 | 150 |
| 158 | hsa-miR-378a-5p | 9 | 144 |
| 159 | hsa-miR-483-3p | 11 | 143 |
| 160 | hsa-miR-339-5p | 9 | 141 |
| 161 | hsa-miR-130b-5p | 6 | 138 |
| 162 | hsa-miR-7974 | 5 | 135 |
| 163 | hsa-miR-34c-5p | 4 | 135 |
| 164 | hsa-miR-200a-5p | 7 | 129 |
| 165 | hsa-miR-30c-1-3p | 6 | 128 |
| 166 | hsa-miR-378i | 11 | 123 |
| 167 | hsa-miR-221-5p | 5 | 119 |
| 168 | hsa-miR-877-5p | 16 | 116 |
| 169 | hsa-miR-152-3p | 10 | 116 |
| 170 | hsa-miR-181c-3p | 5 | 113 |
| 171 | hsa-miR-625-3p | 12 | 112 |
| 172 | hsa-miR-671-3p | 4 | 112 |
| 173 | hsa-miR-374b-5p | 6 | 111 |
| 174 | hsa-miR-516a-5p | 4 | 110 |
| 175 | hsa-miR-19a-3p | 4 | 109 |
| 176 | hsa-miR-149-5p | 7 | 108 |
| 177 | hsa-miR-3607-3p | 3 | 107 |
| 178 | hsa-miR-505-3p | 9 | 103 |
| 179 | hsa-miR-29b-3p | 4 | 101 |
| 180 | hsa-miR-1248 | 2 | 99 |
| 181 | hsa-miR-223-3p | 8 | 99 |
| 182 | hsa-miR-17-3p | 7 | 95 |
| 183 | hsa-miR-374a-5p | 4 | 95 |
| 184 | hsa-miR-652-3p | 8 | 94 |
| 185 | hsa-miR-210-3p | 9 | 91 |
| 186 | hsa-miR-411-5p | 2 | 87 |
| 187 | hsa-miR-501-3p | 6 | 87 |
| 188 | hsa-miR-3196 | 6 | 86 |
| 189 | hsa-miR-93-3p | 5 | 85 |
| 190 | hsa-miR-194-5p | 4 | 85 |
| 191 | hsa-miR-7706 | 6 | 83 |
| 192 | hsa-let-7a-3p | 6 | 83 |
| 193 | hsa-miR-4516 | 11 | 82 |
| 194 | hsa-miR-452-5p | 8 | 80 |
| 195 | hsa-miR-874-3p | 6 | 79 |
| 196 | hsa-miR-409-3p | 6 | 75 |
| 197 | hsa-miR-381-3p | 3 | 75 |
| 198 | hsa-miR-654-3p | 4 | 74 |
| 199 | hsa-miR-195-3p | 6 | 74 |
| 200 | hsa-miR-200b-5p | 6 | 74 |
| 201 | hsa-miR-3687 | 13 | 73 |
| 202 | hsa-miR-199b-5p | 6 | 71 |
| 203 | hsa-miR-483-5p | 6 | 71 |
| 204 | hsa-miR-96-5p | 3 | 67 |
| 205 | hsa-miR-589-5p | 3 | 67 |
| 206 | hsa-miR-1275 | 5 | 64 |
| 207 | hsa-miR-502-3p | 6 | 64 |
| 208 | hsa-miR-582-3p | 2 | 63 |
| 209 | hsa-miR-5096 | 14 | 62 |
| 210 | hsa-miR-30a-3p | 8 | 62 |
| 211 | hsa-miR-338-3p | 3 | 61 |
| 212 | hsa-miR-375 | 3 | 61 |
| 213 | hsa-miR-193a-3p | 4 | 57 |
| 214 | hsa-miR-301b-3p | 1 | 56 |
| 215 | hsa-miR-1260b | 6 | 56 |
| 216 | hsa-miR-598-3p | 5 | 54 |
| 217 | hsa-miR-675-3p | 6 | 53 |
| 218 | hsa-miR-424-5p | 4 | 53 |
| 219 | hsa-miR-26b-3p | 3 | 53 |
| 220 | hsa-miR-25-5p | 2 | 53 |
| 221 | hsa-miR-522-3p | 2 | 51 |
| 222 | hsa-miR-185-5p | 4 | 51 |
| 223 | hsa-miR-449c-5p | 6 | 49 |
| 224 | hsa-miR-142-3p | 5 | 49 |
| 225 | hsa-miR-3609 | 1 | 49 |
| 226 | hsa-miR-1296-5p | 3 | 48 |
| 227 | hsa-miR-7705 | 5 | 46 |
| 228 | hsa-miR-324-5p | 4 | 46 |
| 229 | hsa-miR-548k | 1 | 45 |
| 230 | hsa-miR-125a-3p | 4 | 45 |
| 231 | hsa-miR-4791 | 1 | 44 |
| 232 | hsa-miR-424-3p | 4 | 44 |
| 233 | hsa-let-7e-3p | 5 | 43 |
| 234 | hsa-miR-576-5p | 5 | 43 |
| 235 | hsa-miR-99a-5p | 4 | 42 |
| 236 | hsa-miR-187-3p | 6 | 40 |
| 237 | hsa-miR-582-5p | 3 | 39 |
| 238 | hsa-miR-3615 | 5 | 39 |
| 239 | hsa-miR-378c | 2 | 38 |
| 240 | hsa-miR-625-5p | 3 | 37 |
| 241 | hsa-miR-708-5p | 5 | 37 |
| 242 | hsa-miR-1269a | 6 | 35 |
| 243 | hsa-miR-1260a | 2 | 35 |
| 244 | hsa-miR-675-5p | 4 | 35 |
| 245 | hsa-miR-18a-5p | 4 | 34 |
| 246 | hsa-miR-18a-3p | 4 | 33 |
| 247 | hsa-miR-27a-5p | 2 | 32 |
| 248 | hsa-miR-641 | 3 | 32 |
| 249 | hsa-miR-20b-5p | 3 | 31 |
| 250 | hsa-miR-215-5p | 5 | 31 |
| 251 | hsa-miR-454-5p | 4 | 30 |
| 252 | hsa-miR-6087 | 5 | 30 |
| 253 | hsa-miR-135b-5p | 3 | 29 |
| 254 | hsa-miR-532-3p | 3 | 28 |
| 255 | hsa-miR-196a-5p | 4 | 28 |
| 256 | hsa-miR-410-3p | 1 | 28 |
| 257 | hsa-miR-3200-3p | 3 | 27 |
| 258 | hsa-miR-378g | 4 | 27 |
| 259 | hsa-let-7f-1-3p | 2 | 27 |
| 260 | hsa-miR-561-5p | 1 | 27 |
| 261 | hsa-miR-4286 | 2 | 27 |
| 262 | hsa-miR-326 | 3 | 27 |
| 263 | hsa-miR-200c-5p | 2 | 27 |
| 264 | hsa-miR-374a-3p | 1 | 27 |
| 265 | hsa-miR-330-5p | 2 | 27 |
| 266 | hsa-miR-1468-5p | 1 | 26 |
| 267 | hsa-miR-218-5p | 3 | 26 |
| 268 | hsa-miR-3168 | 5 | 26 |
| 269 | hsa-miR-134-5p | 5 | 26 |
| 270 | hsa-miR-1261 | 4 | 25 |
| 271 | hsa-miR-3607-5p | 1 | 25 |
| 272 | hsa-miR-4326 | 5 | 25 |
| 273 | hsa-miR-125b-2-3p | 3 | 25 |
| 274 | hsa-miR-362-5p | 2 | 24 |
| 275 | hsa-miR-2277-5p | 4 | 24 |
| 276 | hsa-miR-4677-3p | 2 | 24 |
| 277 | hsa-miR-136-3p | 1 | 24 |
| 278 | hsa-miR-328-3p | 5 | 24 |
| 279 | hsa-miR-30d-3p | 3 | 24 |
| 280 | hsa-miR-204-5p | 3 | 24 |
| 281 | hsa-miR-1304-3p | 4 | 23 |
| 282 | hsa-let-7f-2-3p | 1 | 22 |
| 283 | hsa-miR-212-3p | 2 | 22 |
| 284 | hsa-miR-296-5p | 2 | 22 |
| 285 | hsa-miR-29c-5p | 4 | 22 |
| 286 | hsa-miR-548o-3p | 1 | 22 |
| 287 | hsa-miR-214-3p | 3 | 22 |
| 288 | hsa-miR-7-1-3p | 4 | 22 |
| 289 | hsa-miR-15b-3p | 1 | 21 |
| 290 | hsa-miR-106a-5p | 3 | 21 |
| 291 | hsa-miR-1180-3p | 1 | 21 |
| 292 | hsa-miR-519a-3p | 2 | 21 |
| 293 | hsa-miR-577 | 1 | 20 |
| 294 | hsa-miR-145-3p | 3 | 20 |
| 295 | hsa-miR-378d | 1 | 19 |
| 296 | hsa-miR-10b-3p | 3 | 19 |
| 297 | hsa-miR-550a-3p | 2 | 19 |
| 298 | hsa-miR-296-3p | 4 | 18 |
| 299 | hsa-miR-4787-5p | 2 | 18 |
| 300 | hsa-miR-651-5p | 2 | 17 |
| 301 | hsa-miR-1283 | 3 | 16 |
| 302 | hsa-miR-449a | 2 | 16 |
| 303 | hsa-miR-887-3p | 1 | 15 |
| 304 | hsa-miR-140-5p | 2 | 15 |
| 305 | hsa-miR-1285-3p | 3 | 15 |
| 306 | hsa-miR-128-1-5p | 2 | 15 |
| 307 | hsa-miR-3651 | 4 | 15 |
| 308 | hsa-miR-3065-3p | 3 | 15 |
| 309 | hsa-miR-708-3p | 3 | 14 |
| 310 | hsa-miR-32-5p | 2 | 13 |
| 311 | hsa-miR-450b-5p | 2 | 13 |
| 312 | hsa-miR-144-5p | 2 | 13 |
| 313 | hsa-let-7i-3p | 2 | 13 |
| 314 | hsa-miR-340-3p | 1 | 13 |
| 315 | hsa-miR-501-5p | 3 | 13 |
| 316 | hsa-miR-1290 | 2 | 12 |
| 317 | hsa-miR-942-5p | 3 | 12 |
| 318 | hsa-miR-671-5p | 3 | 12 |
| 319 | hsa-miR-183-3p | 3 | 12 |
| 320 | hsa-miR-4284 | 1 | 12 |
| 321 | hsa-miR-493-5p | 1 | 12 |
| 322 | hsa-miR-33b-5p | 1 | 12 |
| 323 | hsa-miR-744-3p | 3 | 12 |
| 324 | hsa-miR-369-5p | 1 | 11 |
| 325 | hsa-miR-500a-5p | 2 | 11 |
| 326 | hsa-miR-92a-1-5p | 1 | 11 |
| 327 | hsa-miR-338-5p | 1 | 10 |
| 328 | hsa-miR-455-5p | 1 | 10 |
| 329 | hsa-miR-29a-5p | 2 | 10 |
| 330 | hsa-miR-874-5p | 2 | 10 |
| 331 | hsa-miR-4662a-5p | 1 | 10 |
| 332 | hsa-miR-664b-3p | 1 | 10 |
| 333 | hsa-miR-24-2-5p | 1 | 10 |
| 334 | hsa-miR-1301-3p | 2 | 10 |
| 335 | hsa-miR-629-5p | 2 | 10 |
| 336 | hsa-miR-378h | 2 | 9 |
| 337 | hsa-miR-4517 | 1 | 9 |
| 338 | hsa-miR-342-5p | 2 | 9 |
| 339 | hsa-miR-664a-5p | 2 | 9 |
| 340 | hsa-miR-125b-1-3p | 1 | 9 |
| 341 | hsa-miR-29b-1-5p | 3 | 9 |
| 342 | hsa-miR-9-5p | 2 | 9 |
| 343 | hsa-miR-144-3p | 2 | 9 |
| 344 | hsa-miR-98-3p | 1 | 9 |
| 345 | hsa-miR-34c-3p | 1 | 8 |
| 346 | hsa-miR-210-5p | 1 | 8 |
| 347 | hsa-miR-450a-5p | 2 | 8 |
| 348 | hsa-miR-3648 | 2 | 8 |
| 349 | hsa-miR-34b-5p | 2 | 8 |
| 350 | hsa-miR-2110 | 2 | 8 |
| 351 | hsa-miR-382-5p | 1 | 8 |
| 352 | hsa-miR-493-3p | 2 | 8 |
| 353 | hsa-miR-4531 | 2 | 7 |
| 354 | hsa-miR-432-5p | 2 | 7 |
| 355 | hsa-miR-4510 | 1 | 7 |
| 356 | hsa-miR-548e-3p | 2 | 7 |
| 357 | hsa-miR-551a | 1 | 7 |
| 358 | hsa-miR-1254 | 1 | 7 |
| 359 | hsa-miR-129-2-3p | 1 | 7 |
| 360 | hsa-miR-22-5p | 2 | 7 |
| 361 | hsa-miR-132-5p | 1 | 7 |
| 362 | hsa-miR-370-3p | 1 | 7 |
| 363 | hsa-miR-589-3p | 1 | 7 |
| 364 | hsa-miR-4676-5p | 1 | 7 |
| 365 | hsa-miR-23a-5p | 1 | 7 |
| 366 | hsa-miR-619-5p | 2 | 6 |
| 367 | hsa-miR-1276 | 1 | 6 |
| 368 | hsa-miR-485-3p | 1 | 6 |
| 369 | hsa-miR-1266-5p | 1 | 6 |
| 370 | hsa-miR-1287-5p | 2 | 6 |
| 371 | hsa-miR-32-3p | 1 | 6 |
| 372 | hsa-miR-212-5p | 1 | 6 |
| 373 | hsa-miR-3653-5p | 1 | 6 |
| 374 | hsa-miR-30c-2-3p | 1 | 6 |
| 375 | hsa-miR-487b-3p | 1 | 6 |
| 376 | hsa-miR-425-3p | 2 | 6 |
| 377 | hsa-miR-34a-3p | 2 | 6 |
| 378 | hsa-miR-377-5p | 1 | 6 |
| 379 | hsa-miR-214-5p | 1 | 6 |
| 380 | hsa-miR-224-3p | 2 | 6 |
| 381 | hsa-miR-148b-5p | 1 | 6 |
| 382 | hsa-miR-3909 | 1 | 6 |
| 383 | hsa-miR-136-5p | 1 | 5 |
| 384 | hsa-miR-934 | 1 | 5 |
| 385 | hsa-miR-31-3p | 1 | 5 |
| 386 | hsa-miR-3605-3p | 1 | 5 |
| 387 | hsa-miR-889-3p | 1 | 5 |
| 388 | hsa-miR-576-3p | 1 | 5 |
| 389 | hsa-miR-518d-3p | 1 | 5 |
| 390 | hsa-let-7g-3p | 1 | 5 |
| 391 | hsa-miR-1271-5p | 1 | 5 |
| 392 | hsa-miR-758-3p | 1 | 5 |
| 393 | hsa-miR-193b-5p | 1 | 5 |
| 394 | hsa-miR-143-5p | 1 | 5 |
| 395 | hsa-miR-139-5p | 1 | 5 |
| 396 | hsa-miR-129-5p | 1 | 5 |
| 397 | hsa-miR-323b-3p | 1 | 5 |
| 398 | hsa-miR-431-5p | 1 | 5 |
| 399 | hsa-miR-3614-5p | 1 | 4 |
| 400 | hsa-miR-191-3p | 1 | 4 |
| 401 | hsa-miR-365b-5p | 1 | 4 |
| 402 | hsa-miR-520f-5p | 1 | 4 |
| 403 | hsa-miR-548ah-3p | 1 | 4 |
| 404 | hsa-miR-378e | 1 | 4 |
| 405 | hsa-miR-135a-5p | 1 | 4 |
| 406 | hsa-miR-624-5p | 1 | 4 |
| 407 | hsa-miR-4449 | 1 | 4 |
| 408 | hsa-miR-590-3p | 1 | 4 |
| 409 | hsa-miR-4652-5p | 1 | 4 |
| 410 | hsa-miR-509-3p | 1 | 4 |
| 411 | hsa-miR-7-5p | 1 | 4 |
| 412 | hsa-miR-3690 | 1 | 4 |
| 413 | hsa-miR-99a-3p | 1 | 4 |
| 414 | hsa-miR-1255a | 1 | 4 |
| 415 | hsa-miR-1-3p | 1 | 4 |
| 416 | hsa-miR-628-5p | 1 | 4 |
| 417 | hsa-miR-2467-5p | 1 | 4 |
| 418 | hsa-miR-29b-2-5p | 1 | 4 |
| 419 | hsa-miR-34b-3p | 1 | 4 |
| 420 | hsa-miR-186-3p | 1 | 4 |
| 421 | hsa-miR-584-5p | 1 | 4 |
| 422 | hsa-miR-548ab | 1 | 3 |
| 423 | hsa-miR-301a-5p | 1 | 3 |
| 424 | hsa-miR-663b | 1 | 3 |
| 425 | hsa-miR-374b-3p | 1 | 3 |
| 426 | hsa-miR-331-5p | 1 | 3 |
| 427 | hsa-miR-494-3p | 1 | 3 |
| 428 | hsa-miR-92b-5p | 1 | 3 |
| 429 | hsa-miR-376c-3p | 1 | 3 |
| 430 | hsa-miR-4488 | 1 | 3 |
| 431 | hsa-miR-1908-5p | 1 | 3 |
| 432 | hsa-miR-940 | 1 | 3 |
| 433 | hsa-miR-7703 | 1 | 3 |
| 434 | hsa-miR-548w | 1 | 3 |
| 435 | hsa-miR-937-3p | 1 | 3 |
| 436 | hsa-miR-4435 | 1 | 3 |
| 437 | hsa-miR-135b-3p | 1 | 3 |
| 438 | hsa-miR-152-5p | 1 | 3 |
| 439 | hsa-miR-127-5p | 1 | 3 |
| 440 | hsa-miR-455-3p | 1 | 3 |
| 441 | hsa-miR-203b-3p | 1 | 3 |
| 442 | hsa-miR-147b | 1 | 3 |
| 443 | hsa-miR-505-5p | 1 | 3 |
| 444 | hsa-miR-769-3p | 1 | 3 |
| 445 | hsa-miR-6842-3p | 1 | 3 |
| 446 | hsa-miR-3065-5p | 1 | 3 |
| 447 | hsa-miR-323a-3p | 1 | 3 |
| 448 | hsa-miR-2355-5p | 1 | 3 |
| 449 | hsa-miR-219a-1-3p | 1 | 3 |
| 450 | hsa-miR-3622a-5p | 1 | 3 |
| 451 | hsa-miR-2355-3p | 1 | 3 |
| 452 | hsa-miR-377-3p | 1 | 3 |
| 453 | hsa-miR-4746-5p | 1 | 3 |
| 454 | hsa-miR-628-3p | 1 | 3 |
| 455 | hsa-miR-3613-3p | 1 | 3 |
| 456 | hsa-miR-542-3p | 1 | 3 |
| 457 | hsa-miR-3116 | 1 | 3 |
| 458 | hsa-miR-184 | 1 | 3 |
| 459 | hsa-miR-26a-2-3p | 1 | 3 |

Table S2C. Number of unique reads and read count of miRNAs detected in SOCa

| **S. N.** | **miRNA** | **unique Reads** | **Read Count** |
| --- | --- | --- | --- |
| 1 | hsa-miR-10b-5p | 801 | 663694 |
| 2 | hsa-miR-10a-5p | 728 | 445720 |
| 3 | hsa-miR-21-5p | 487 | 326344 |
| 4 | hsa-miR-26a-5p | 349 | 314055 |
| 5 | hsa-miR-27b-3p | 219 | 187356 |
| 6 | hsa-miR-148a-3p | 354 | 158992 |
| 7 | hsa-let-7a-5p | 137 | 132232 |
| 8 | hsa-miR-141-3p | 168 | 128498 |
| 9 | hsa-miR-22-3p | 167 | 125037 |
| 10 | hsa-miR-143-3p | 281 | 107681 |
| 11 | hsa-miR-3182 | 45 | 107122 |
| 12 | hsa-miR-146b-5p | 264 | 93937 |
| 13 | hsa-miR-181a-5p | 223 | 77365 |
| 14 | hsa-miR-92a-3p | 393 | 70877 |
| 15 | hsa-miR-1273g-3p | 154 | 53749 |
| 16 | hsa-let-7f-5p | 118 | 47930 |
| 17 | hsa-miR-191-5p | 148 | 45559 |
| 18 | hsa-miR-92b-3p | 447 | 36495 |
| 19 | hsa-miR-16-5p | 113 | 36086 |
| 20 | hsa-miR-30d-5p | 153 | 30632 |
| 21 | hsa-miR-151a-5p | 90 | 28308 |
| 22 | hsa-miR-99b-5p | 167 | 27155 |
| 23 | hsa-miR-182-5p | 142 | 26655 |
| 24 | hsa-miR-30e-5p | 162 | 25893 |
| 25 | hsa-miR-125a-5p | 165 | 25658 |
| 26 | hsa-let-7b-5p | 137 | 25344 |
| 27 | hsa-miR-21-3p | 130 | 23084 |
| 28 | hsa-let-7i-5p | 100 | 22347 |
| 29 | hsa-miR-151a-3p | 176 | 22193 |
| 30 | hsa-miR-25-3p | 110 | 19974 |
| 31 | hsa-miR-30a-5p | 102 | 19947 |
| 32 | hsa-miR-27a-3p | 95 | 19269 |
| 33 | hsa-let-7g-5p | 71 | 19120 |
| 34 | hsa-miR-378a-3p | 97 | 17098 |
| 35 | hsa-miR-200b-3p | 127 | 15260 |
| 36 | hsa-miR-93-5p | 93 | 14363 |
| 37 | hsa-miR-186-5p | 94 | 14341 |
| 38 | hsa-miR-101-3p | 97 | 13841 |
| 39 | hsa-miR-26b-5p | 77 | 11524 |
| 40 | hsa-miR-29a-3p | 91 | 10770 |
| 41 | hsa-miR-192-5p | 95 | 10452 |
| 42 | hsa-miR-221-3p | 85 | 10326 |
| 43 | hsa-miR-28-3p | 86 | 9577 |
| 44 | hsa-miR-130a-3p | 62 | 9208 |
| 45 | hsa-miR-126-5p | 43 | 8953 |
| 46 | hsa-miR-100-5p | 79 | 8394 |
| 47 | hsa-miR-200c-3p | 72 | 7890 |
| 48 | hsa-miR-103a-3p | 44 | 7879 |
| 49 | hsa-miR-181c-5p | 66 | 7819 |
| 50 | hsa-miR-30c-5p | 55 | 7396 |
| 51 | hsa-let-7e-5p | 52 | 7321 |
| 52 | hsa-miR-486-5p | 61 | 6584 |
| 53 | hsa-miR-23b-3p | 75 | 6172 |
| 54 | hsa-miR-222-3p | 76 | 6168 |
| 55 | hsa-miR-199a-5p | 75 | 5903 |
| 56 | hsa-miR-125b-5p | 74 | 5543 |
| 57 | hsa-miR-142-5p | 42 | 5432 |
| 58 | hsa-miR-200a-3p | 50 | 5375 |
| 59 | hsa-miR-30b-5p | 36 | 5326 |
| 60 | hsa-miR-181b-5p | 77 | 5114 |
| 61 | hsa-miR-24-3p | 46 | 4887 |
| 62 | hsa-miR-484 | 84 | 4795 |
| 63 | hsa-miR-146a-5p | 61 | 4684 |
| 64 | hsa-miR-23a-3p | 56 | 4353 |
| 65 | hsa-let-7c-5p | 27 | 4232 |
| 66 | hsa-miR-155-5p | 59 | 3938 |
| 67 | hsa-miR-429 | 42 | 3663 |
| 68 | hsa-miR-451a | 45 | 3566 |
| 69 | hsa-miR-98-5p | 28 | 3541 |
| 70 | hsa-miR-148b-3p | 46 | 3299 |
| 71 | hsa-miR-423-3p | 60 | 3004 |
| 72 | hsa-miR-345-5p | 36 | 2897 |
| 73 | hsa-miR-320a | 60 | 2799 |
| 74 | hsa-let-7d-5p | 26 | 2770 |
| 75 | hsa-miR-183-5p | 44 | 2705 |
| 76 | hsa-miR-31-5p | 26 | 2655 |
| 77 | hsa-miR-335-3p | 34 | 2549 |
| 78 | hsa-miR-199b-5p | 31 | 2533 |
| 79 | hsa-miR-5684 | 9 | 2487 |
| 80 | hsa-miR-15a-5p | 27 | 2075 |
| 81 | hsa-miR-224-5p | 30 | 2062 |
| 82 | hsa-miR-128-3p | 47 | 1974 |
| 83 | hsa-miR-4454 | 30 | 1917 |
| 84 | hsa-miR-425-5p | 32 | 1905 |
| 85 | hsa-miR-99a-5p | 39 | 1892 |
| 86 | hsa-miR-19b-3p | 14 | 1790 |
| 87 | hsa-miR-4448 | 12 | 1790 |
| 88 | hsa-miR-126-3p | 33 | 1735 |
| 89 | hsa-miR-140-3p | 47 | 1722 |
| 90 | hsa-miR-203a-3p | 40 | 1682 |
| 91 | hsa-miR-708-5p | 30 | 1677 |
| 92 | hsa-miR-106b-5p | 28 | 1584 |
| 93 | hsa-miR-193b-3p | 39 | 1546 |
| 94 | hsa-miR-20a-5p | 21 | 1480 |
| 95 | hsa-miR-210-3p | 35 | 1456 |
| 96 | hsa-miR-30e-3p | 23 | 1360 |
| 97 | hsa-miR-28-5p | 23 | 1317 |
| 98 | hsa-miR-148a-5p | 19 | 1309 |
| 99 | hsa-miR-181d-5p | 26 | 1282 |
| 100 | hsa-miR-340-5p | 20 | 1234 |
| 101 | hsa-miR-411-5p | 12 | 1232 |
| 102 | hsa-miR-769-5p | 18 | 1170 |
| 103 | hsa-miR-4532 | 23 | 1162 |
| 104 | hsa-miR-17-5p | 15 | 1118 |
| 105 | hsa-miR-654-3p | 32 | 1105 |
| 106 | hsa-miR-141-5p | 17 | 1055 |
| 107 | hsa-miR-342-3p | 27 | 1046 |
| 108 | hsa-miR-29c-3p | 26 | 978 |
| 109 | hsa-miR-423-5p | 18 | 971 |
| 110 | hsa-miR-152-3p | 28 | 938 |
| 111 | hsa-miR-146b-3p | 22 | 920 |
| 112 | hsa-miR-1307-5p | 26 | 900 |
| 113 | hsa-miR-574-3p | 20 | 886 |
| 114 | hsa-miR-15b-5p | 12 | 858 |
| 115 | hsa-miR-532-5p | 25 | 851 |
| 116 | hsa-miR-941 | 16 | 848 |
| 117 | hsa-miR-361-5p | 19 | 840 |
| 118 | hsa-miR-150-5p | 14 | 832 |
| 119 | hsa-miR-335-5p | 13 | 802 |
| 120 | hsa-miR-142-3p | 23 | 797 |
| 121 | hsa-miR-34a-5p | 20 | 796 |
| 122 | hsa-miR-106b-3p | 25 | 794 |
| 123 | hsa-miR-197-3p | 19 | 776 |
| 124 | hsa-miR-361-3p | 20 | 741 |
| 125 | hsa-miR-381-3p | 18 | 681 |
| 126 | hsa-miR-195-5p | 13 | 627 |
| 127 | hsa-miR-34c-5p | 11 | 607 |
| 128 | hsa-miR-136-3p | 14 | 560 |
| 129 | hsa-miR-708-3p | 16 | 538 |
| 130 | hsa-miR-409-3p | 14 | 531 |
| 131 | hsa-miR-181a-2-3p | 17 | 525 |
| 132 | hsa-miR-454-3p | 10 | 522 |
| 133 | hsa-miR-301a-3p | 8 | 521 |
| 134 | hsa-miR-204-5p | 10 | 496 |
| 135 | hsa-miR-181a-3p | 17 | 484 |
| 136 | hsa-miR-4792 | 14 | 480 |
| 137 | hsa-miR-127-3p | 51 | 476 |
| 138 | hsa-miR-194-5p | 12 | 472 |
| 139 | hsa-miR-130b-3p | 10 | 460 |
| 140 | hsa-miR-625-3p | 24 | 433 |
| 141 | hsa-miR-125b-2-3p | 15 | 425 |
| 142 | hsa-miR-1307-3p | 22 | 413 |
| 143 | hsa-miR-30a-3p | 17 | 412 |
| 144 | hsa-miR-452-5p | 20 | 412 |
| 145 | hsa-miR-7977 | 3 | 412 |
| 146 | hsa-miR-500a-3p | 8 | 405 |
| 147 | hsa-miR-331-3p | 17 | 403 |
| 148 | hsa-miR-363-3p | 14 | 391 |
| 149 | hsa-miR-135b-5p | 12 | 390 |
| 150 | hsa-miR-181c-3p | 11 | 388 |
| 151 | hsa-miR-421 | 9 | 378 |
| 152 | hsa-miR-374a-5p | 6 | 362 |
| 153 | hsa-miR-652-3p | 14 | 360 |
| 154 | hsa-miR-19a-3p | 5 | 353 |
| 155 | hsa-miR-582-3p | 11 | 353 |
| 156 | hsa-miR-29b-3p | 11 | 352 |
| 157 | hsa-miR-339-3p | 14 | 343 |
| 158 | hsa-miR-660-5p | 8 | 339 |
| 159 | hsa-miR-96-5p | 4 | 338 |
| 160 | hsa-let-7b-3p | 10 | 328 |
| 161 | hsa-miR-874-3p | 16 | 319 |
| 162 | hsa-miR-4497 | 11 | 318 |
| 163 | hsa-miR-135a-5p | 9 | 316 |
| 164 | hsa-miR-375 | 8 | 311 |
| 165 | hsa-miR-214-3p | 15 | 307 |
| 166 | hsa-miR-424-5p | 10 | 307 |
| 167 | hsa-miR-374a-3p | 4 | 303 |
| 168 | hsa-miR-574-5p | 16 | 302 |
| 169 | hsa-miR-1246 | 19 | 289 |
| 170 | hsa-miR-32-5p | 5 | 282 |
| 171 | hsa-miR-205-5p | 10 | 273 |
| 172 | hsa-miR-221-5p | 7 | 265 |
| 173 | hsa-miR-187-3p | 15 | 263 |
| 174 | hsa-let-7d-3p | 8 | 258 |
| 175 | hsa-miR-145-5p | 10 | 254 |
| 176 | hsa-miR-218-5p | 9 | 252 |
| 177 | hsa-miR-410-3p | 5 | 250 |
| 178 | hsa-miR-125b-1-3p | 9 | 247 |
| 179 | hsa-miR-744-5p | 7 | 246 |
| 180 | hsa-miR-132-3p | 8 | 241 |
| 181 | hsa-miR-223-3p | 13 | 241 |
| 182 | hsa-miR-7704 | 10 | 236 |
| 183 | hsa-miR-30d-3p | 12 | 232 |
| 184 | hsa-miR-200a-5p | 6 | 231 |
| 185 | hsa-miR-4508 | 6 | 228 |
| 186 | hsa-miR-450b-5p | 6 | 224 |
| 187 | hsa-miR-7641 | 8 | 222 |
| 188 | hsa-miR-339-5p | 10 | 222 |
| 189 | hsa-miR-193a-5p | 9 | 221 |
| 190 | hsa-miR-27b-5p | 4 | 220 |
| 191 | hsa-miR-582-5p | 9 | 210 |
| 192 | hsa-miR-625-5p | 6 | 202 |
| 193 | hsa-miR-497-5p | 5 | 193 |
| 194 | hsa-miR-5701 | 3 | 192 |
| 195 | hsa-miR-214-5p | 7 | 189 |
| 196 | hsa-miR-5100 | 9 | 188 |
| 197 | hsa-miR-145-3p | 7 | 188 |
| 198 | hsa-miR-589-5p | 6 | 184 |
| 199 | hsa-miR-374b-5p | 5 | 184 |
| 200 | hsa-miR-33b-5p | 2 | 178 |
| 201 | hsa-miR-215-5p | 17 | 177 |
| 202 | hsa-miR-10a-3p | 10 | 174 |
| 203 | hsa-miR-190a-5p | 7 | 172 |
| 204 | hsa-let-7a-3p | 7 | 172 |
| 205 | hsa-miR-134-5p | 13 | 171 |
| 206 | hsa-miR-144-3p | 3 | 168 |
| 207 | hsa-miR-1260b | 9 | 163 |
| 208 | hsa-miR-501-3p | 7 | 163 |
| 209 | hsa-miR-17-3p | 7 | 160 |
| 210 | hsa-miR-4301 | 8 | 159 |
| 211 | hsa-miR-378a-5p | 7 | 155 |
| 212 | hsa-miR-671-3p | 5 | 155 |
| 213 | hsa-let-7i-3p | 6 | 154 |
| 214 | hsa-miR-99b-3p | 13 | 153 |
| 215 | hsa-miR-107 | 4 | 152 |
| 216 | hsa-miR-664a-3p | 7 | 150 |
| 217 | hsa-miR-200b-5p | 7 | 149 |
| 218 | hsa-miR-1296-5p | 7 | 145 |
| 219 | hsa-miR-455-5p | 7 | 144 |
| 220 | hsa-miR-7-5p | 6 | 142 |
| 221 | hsa-miR-493-5p | 3 | 139 |
| 222 | hsa-miR-493-3p | 5 | 139 |
| 223 | hsa-miR-3195 | 10 | 137 |
| 224 | hsa-miR-576-5p | 4 | 134 |
| 225 | hsa-miR-550a-3p | 7 | 132 |
| 226 | hsa-miR-185-5p | 6 | 114 |
| 227 | hsa-miR-328-3p | 9 | 114 |
| 228 | hsa-miR-136-5p | 2 | 113 |
| 229 | hsa-miR-615-3p | 7 | 113 |
| 230 | hsa-miR-22-5p | 6 | 111 |
| 231 | hsa-miR-3960 | 19 | 110 |
| 232 | hsa-miR-4531 | 3 | 110 |
| 233 | hsa-miR-193a-3p | 8 | 110 |
| 234 | hsa-miR-15b-3p | 6 | 109 |
| 235 | hsa-miR-1285-3p | 7 | 109 |
| 236 | hsa-miR-144-5p | 5 | 106 |
| 237 | hsa-miR-125a-3p | 6 | 105 |
| 238 | hsa-miR-2467-5p | 4 | 104 |
| 239 | hsa-miR-449c-5p | 6 | 99 |
| 240 | hsa-miR-10b-3p | 6 | 98 |
| 241 | hsa-miR-3687 | 15 | 97 |
| 242 | hsa-miR-450a-5p | 6 | 96 |
| 243 | hsa-miR-301b-3p | 2 | 95 |
| 244 | hsa-miR-4516 | 13 | 93 |
| 245 | hsa-miR-4492 | 3 | 93 |
| 246 | hsa-miR-1301-3p | 11 | 92 |
| 247 | hsa-miR-449a | 2 | 89 |
| 248 | hsa-miR-889-3p | 3 | 87 |
| 249 | hsa-miR-3196 | 8 | 87 |
| 250 | hsa-miR-505-3p | 8 | 86 |
| 251 | hsa-miR-455-3p | 8 | 84 |
| 252 | hsa-miR-140-5p | 5 | 79 |
| 253 | hsa-miR-502-3p | 6 | 79 |
| 254 | hsa-miR-93-3p | 3 | 77 |
| 255 | hsa-miR-378c | 2 | 77 |
| 256 | hsa-miR-4286 | 2 | 76 |
| 257 | hsa-miR-18a-5p | 6 | 75 |
| 258 | hsa-miR-548k | 1 | 75 |
| 259 | hsa-miR-324-5p | 5 | 74 |
| 260 | hsa-miR-598-3p | 6 | 74 |
| 261 | hsa-miR-1260a | 3 | 73 |
| 262 | hsa-miR-424-3p | 5 | 73 |
| 263 | hsa-miR-26a-2-3p | 3 | 72 |
| 264 | hsa-miR-1271-5p | 4 | 71 |
| 265 | hsa-miR-378d | 3 | 69 |
| 266 | hsa-miR-130b-5p | 4 | 68 |
| 267 | hsa-miR-376c-3p | 4 | 67 |
| 268 | hsa-miR-149-5p | 5 | 67 |
| 269 | hsa-miR-369-5p | 3 | 65 |
| 270 | hsa-miR-885-5p | 3 | 64 |
| 271 | hsa-miR-3615 | 5 | 63 |
| 272 | hsa-miR-542-3p | 5 | 63 |
| 273 | hsa-miR-590-3p | 4 | 63 |
| 274 | hsa-miR-27a-5p | 2 | 62 |
| 275 | hsa-miR-432-5p | 6 | 61 |
| 276 | hsa-miR-409-5p | 6 | 61 |
| 277 | hsa-miR-330-5p | 3 | 55 |
| 278 | hsa-let-7f-2-3p | 3 | 55 |
| 279 | hsa-miR-7706 | 5 | 55 |
| 280 | hsa-miR-196b-5p | 5 | 54 |
| 281 | hsa-miR-30c-1-3p | 4 | 54 |
| 282 | hsa-miR-629-5p | 6 | 54 |
| 283 | hsa-miR-431-5p | 5 | 52 |
| 284 | hsa-miR-7974 | 3 | 52 |
| 285 | hsa-miR-3607-3p | 3 | 51 |
| 286 | hsa-miR-379-5p | 4 | 49 |
| 287 | hsa-miR-671-5p | 7 | 48 |
| 288 | hsa-miR-3176 | 9 | 47 |
| 289 | hsa-miR-758-3p | 4 | 47 |
| 290 | hsa-miR-378i | 7 | 47 |
| 291 | hsa-miR-3168 | 8 | 46 |
| 292 | hsa-miR-4677-3p | 3 | 46 |
| 293 | hsa-miR-887-3p | 4 | 46 |
| 294 | hsa-miR-127-5p | 6 | 44 |
| 295 | hsa-miR-320d | 7 | 44 |
| 296 | hsa-miR-25-5p | 3 | 43 |
| 297 | hsa-miR-1180-3p | 2 | 43 |
| 298 | hsa-let-7e-3p | 5 | 42 |
| 299 | hsa-miR-34b-5p | 3 | 42 |
| 300 | hsa-miR-487b-3p | 5 | 42 |
| 301 | hsa-miR-7-1-3p | 6 | 41 |
| 302 | hsa-miR-548o-3p | 2 | 41 |
| 303 | hsa-miR-382-5p | 5 | 41 |
| 304 | hsa-let-7f-1-3p | 2 | 41 |
| 305 | hsa-miR-3613-5p | 3 | 40 |
| 306 | hsa-miR-26b-3p | 3 | 38 |
| 307 | hsa-miR-935 | 3 | 38 |
| 308 | hsa-miR-196a-5p | 4 | 38 |
| 309 | hsa-miR-370-3p | 4 | 38 |
| 310 | hsa-miR-99a-3p | 4 | 37 |
| 311 | hsa-miR-106a-5p | 5 | 37 |
| 312 | hsa-miR-1270 | 5 | 36 |
| 313 | hsa-miR-212-3p | 3 | 36 |
| 314 | hsa-let-7g-3p | 3 | 35 |
| 315 | hsa-miR-651-5p | 3 | 34 |
| 316 | hsa-miR-449b-5p | 5 | 34 |
| 317 | hsa-miR-210-5p | 3 | 34 |
| 318 | hsa-miR-489-3p | 2 | 34 |
| 319 | hsa-miR-340-3p | 3 | 34 |
| 320 | hsa-miR-4791 | 1 | 34 |
| 321 | hsa-miR-143-5p | 4 | 32 |
| 322 | hsa-miR-338-3p | 2 | 31 |
| 323 | hsa-miR-548ah-3p | 2 | 31 |
| 324 | hsa-miR-20b-5p | 5 | 31 |
| 325 | hsa-miR-6131 | 1 | 30 |
| 326 | hsa-miR-1248 | 5 | 30 |
| 327 | hsa-miR-195-3p | 5 | 30 |
| 328 | hsa-miR-877-5p | 7 | 29 |
| 329 | hsa-miR-1277-5p | 3 | 29 |
| 330 | hsa-miR-1304-3p | 3 | 28 |
| 331 | hsa-miR-92a-1-5p | 1 | 28 |
| 332 | hsa-miR-550a-5p | 7 | 27 |
| 333 | hsa-miR-320b | 3 | 27 |
| 334 | hsa-miR-4449 | 5 | 27 |
| 335 | hsa-miR-152-5p | 1 | 26 |
| 336 | hsa-miR-9-5p | 2 | 26 |
| 337 | hsa-miR-532-3p | 3 | 26 |
| 338 | hsa-miR-369-3p | 2 | 26 |
| 339 | hsa-miR-3158-3p | 3 | 25 |
| 340 | hsa-miR-299-3p | 2 | 25 |
| 341 | hsa-miR-24-2-5p | 3 | 25 |
| 342 | hsa-miR-425-3p | 4 | 24 |
| 343 | hsa-miR-154-5p | 1 | 24 |
| 344 | hsa-miR-1261 | 3 | 24 |
| 345 | hsa-miR-128-1-5p | 2 | 24 |
| 346 | hsa-miR-326 | 3 | 24 |
| 347 | hsa-miR-539-3p | 4 | 24 |
| 348 | hsa-miR-3609 | 2 | 23 |
| 349 | hsa-miR-324-3p | 6 | 23 |
| 350 | hsa-miR-31-3p | 3 | 23 |
| 351 | hsa-miR-98-3p | 2 | 22 |
| 352 | hsa-miR-4326 | 3 | 22 |
| 353 | hsa-miR-29c-5p | 4 | 22 |
| 354 | hsa-miR-342-5p | 5 | 22 |
| 355 | hsa-miR-30c-2-3p | 2 | 21 |
| 356 | hsa-miR-744-3p | 4 | 21 |
| 357 | hsa-miR-548ah-5p | 3 | 21 |
| 358 | hsa-miR-874-5p | 3 | 21 |
| 359 | hsa-miR-362-5p | 3 | 21 |
| 360 | hsa-miR-641 | 2 | 21 |
| 361 | hsa-miR-296-5p | 2 | 20 |
| 362 | hsa-miR-133a-3p | 2 | 20 |
| 363 | hsa-miR-653-5p | 2 | 20 |
| 364 | hsa-miR-1275 | 3 | 20 |
| 365 | hsa-miR-135b-3p | 4 | 20 |
| 366 | hsa-miR-576-3p | 3 | 20 |
| 367 | hsa-miR-374b-3p | 1 | 20 |
| 368 | hsa-miR-628-5p | 3 | 19 |
| 369 | hsa-miR-203b-3p | 3 | 19 |
| 370 | hsa-miR-3200-3p | 3 | 19 |
| 371 | hsa-miR-7705 | 3 | 19 |
| 372 | hsa-miR-656-3p | 1 | 18 |
| 373 | hsa-miR-376a-3p | 1 | 18 |
| 374 | hsa-miR-331-5p | 2 | 18 |
| 375 | hsa-miR-2277-5p | 4 | 18 |
| 376 | hsa-miR-548e-3p | 2 | 18 |
| 377 | hsa-miR-411-3p | 2 | 18 |
| 378 | hsa-miR-942-5p | 4 | 18 |
| 379 | hsa-miR-4787-5p | 4 | 18 |
| 380 | hsa-miR-3913-5p | 3 | 17 |
| 381 | hsa-miR-224-3p | 3 | 17 |
| 382 | hsa-miR-132-5p | 1 | 17 |
| 383 | hsa-miR-3607-5p | 1 | 17 |
| 384 | hsa-miR-34c-3p | 3 | 16 |
| 385 | hsa-miR-664b-3p | 1 | 16 |
| 386 | hsa-miR-100-3p | 1 | 16 |
| 387 | hsa-miR-193b-5p | 2 | 16 |
| 388 | hsa-miR-577 | 1 | 16 |
| 389 | hsa-miR-147b | 2 | 15 |
| 390 | hsa-miR-485-3p | 1 | 15 |
| 391 | hsa-miR-377-3p | 2 | 14 |
| 392 | hsa-miR-3651 | 3 | 14 |
| 393 | hsa-miR-18a-3p | 3 | 14 |
| 394 | hsa-miR-92b-5p | 2 | 14 |
| 395 | hsa-miR-95-3p | 1 | 14 |
| 396 | hsa-miR-16-2-3p | 3 | 14 |
| 397 | hsa-miR-1185-1-3p | 2 | 14 |
| 398 | hsa-miR-556-5p | 2 | 14 |
| 399 | hsa-miR-296-3p | 3 | 13 |
| 400 | hsa-miR-33a-5p | 2 | 13 |
| 401 | hsa-miR-1266-5p | 2 | 13 |
| 402 | hsa-miR-200c-5p | 1 | 13 |
| 403 | hsa-miR-30b-3p | 2 | 12 |
| 404 | hsa-miR-190b | 1 | 12 |
| 405 | hsa-miR-940 | 3 | 12 |
| 406 | hsa-miR-3912-3p | 3 | 12 |
| 407 | hsa-miR-186-3p | 1 | 12 |
| 408 | hsa-miR-299-5p | 2 | 12 |
| 409 | hsa-miR-665 | 1 | 12 |
| 410 | hsa-miR-5096 | 4 | 12 |
| 411 | hsa-miR-551a | 2 | 12 |
| 412 | hsa-miR-219a-1-3p | 2 | 12 |
| 413 | hsa-miR-485-5p | 2 | 11 |
| 414 | hsa-miR-487a-3p | 1 | 11 |
| 415 | hsa-miR-4510 | 1 | 11 |
| 416 | hsa-miR-548y | 1 | 11 |
| 417 | hsa-miR-3934-5p | 2 | 11 |
| 418 | hsa-miR-2355-5p | 2 | 11 |
| 419 | hsa-miR-139-5p | 1 | 11 |
| 420 | hsa-miR-148b-5p | 2 | 11 |
| 421 | hsa-miR-337-3p | 2 | 11 |
| 422 | hsa-miR-659-5p | 2 | 11 |
| 423 | hsa-miR-584-5p | 2 | 11 |
| 424 | hsa-miR-1468-5p | 1 | 10 |
| 425 | hsa-miR-483-3p | 2 | 10 |
| 426 | hsa-miR-378g | 2 | 10 |
| 427 | hsa-miR-3677-3p | 2 | 10 |
| 428 | hsa-miR-330-3p | 2 | 10 |
| 429 | hsa-miR-153-3p | 1 | 10 |
| 430 | hsa-miR-34a-3p | 2 | 10 |
| 431 | hsa-miR-516a-5p | 1 | 9 |
| 432 | hsa-miR-4284 | 2 | 9 |
| 433 | hsa-miR-542-5p | 2 | 9 |
| 434 | hsa-miR-23a-5p | 2 | 9 |
| 435 | hsa-miR-29b-1-5p | 2 | 9 |
| 436 | hsa-miR-337-5p | 2 | 9 |
| 437 | hsa-miR-101-5p | 2 | 9 |
| 438 | hsa-miR-377-5p | 1 | 8 |
| 439 | hsa-miR-499a-5p | 2 | 8 |
| 440 | hsa-miR-379-3p | 2 | 8 |
| 441 | hsa-miR-561-5p | 1 | 8 |
| 442 | hsa-miR-323a-3p | 1 | 8 |
| 443 | hsa-miR-29a-5p | 2 | 8 |
| 444 | hsa-miR-1247-5p | 1 | 8 |
| 445 | hsa-miR-320c | 1 | 8 |
| 446 | hsa-miR-4746-5p | 2 | 8 |
| 447 | hsa-miR-1185-5p | 2 | 8 |
| 448 | hsa-miR-212-5p | 1 | 8 |
| 449 | hsa-miR-183-3p | 2 | 8 |
| 450 | hsa-miR-4455 | 2 | 8 |
| 451 | hsa-miR-1197 | 1 | 8 |
| 452 | hsa-miR-1287-5p | 2 | 8 |
| 453 | hsa-miR-3690 | 2 | 8 |
| 454 | hsa-miR-191-3p | 1 | 7 |
| 455 | hsa-miR-378e | 2 | 7 |
| 456 | hsa-miR-664a-5p | 2 | 7 |
| 457 | hsa-miR-652-5p | 2 | 7 |
| 458 | hsa-miR-188-5p | 2 | 7 |
| 459 | hsa-miR-323b-3p | 1 | 6 |
| 460 | hsa-miR-519a-3p | 1 | 6 |
| 461 | hsa-miR-4728-3p | 2 | 6 |
| 462 | hsa-miR-4662a-5p | 1 | 6 |
| 463 | hsa-miR-548az-5p | 1 | 6 |
| 464 | hsa-miR-1290 | 1 | 6 |
| 465 | hsa-miR-1283 | 2 | 6 |
| 466 | hsa-miR-1251-5p | 1 | 6 |
| 467 | hsa-miR-32-3p | 1 | 6 |
| 468 | hsa-miR-1254 | 1 | 6 |
| 469 | hsa-miR-766-3p | 1 | 6 |
| 470 | hsa-miR-181b-3p | 1 | 6 |
| 471 | hsa-miR-365a-5p | 1 | 6 |
| 472 | hsa-miR-6087 | 2 | 6 |
| 473 | hsa-miR-616-5p | 1 | 6 |
| 474 | hsa-miR-362-3p | 2 | 6 |
| 475 | hsa-miR-188-3p | 1 | 6 |
| 476 | hsa-miR-1268b | 1 | 6 |
| 477 | hsa-miR-3622a-5p | 2 | 6 |
| 478 | hsa-miR-138-5p | 1 | 6 |
| 479 | hsa-miR-3648 | 1 | 5 |
| 480 | hsa-miR-934 | 1 | 5 |
| 481 | hsa-miR-3116 | 1 | 5 |
| 482 | hsa-miR-2114-3p | 1 | 5 |
| 483 | hsa-miR-6765-3p | 1 | 5 |
| 484 | hsa-miR-34b-3p | 1 | 5 |
| 485 | hsa-miR-3180-3p | 1 | 4 |
| 486 | hsa-miR-4758-3p | 1 | 4 |
| 487 | hsa-miR-653-3p | 1 | 4 |
| 488 | hsa-miR-380-3p | 1 | 4 |
| 489 | hsa-miR-3909 | 1 | 4 |
| 490 | hsa-miR-548j-5p | 1 | 4 |
| 491 | hsa-miR-503-5p | 1 | 4 |
| 492 | hsa-miR-548e-5p | 1 | 4 |
| 493 | hsa-miR-4517 | 1 | 4 |
| 494 | hsa-miR-24-1-5p | 1 | 4 |
| 495 | hsa-miR-549a | 1 | 4 |
| 496 | hsa-miR-548i | 1 | 4 |
| 497 | hsa-miR-3614-5p | 1 | 4 |
| 498 | hsa-miR-6843-3p | 1 | 4 |
| 499 | hsa-miR-376b-3p | 1 | 4 |
| 500 | hsa-miR-548x-3p | 1 | 4 |
| 501 | hsa-miR-495-3p | 1 | 4 |
| 502 | hsa-miR-1827 | 1 | 4 |
| 503 | hsa-miR-548d-5p | 1 | 4 |
| 504 | hsa-miR-1285-5p | 1 | 4 |
| 505 | hsa-miR-4443 | 1 | 4 |
| 506 | hsa-miR-4775 | 1 | 4 |
| 507 | hsa-miR-185-3p | 1 | 3 |
| 508 | hsa-miR-5579-3p | 1 | 3 |
| 509 | hsa-miR-873-5p | 1 | 3 |
| 510 | hsa-miR-483-5p | 1 | 3 |
| 511 | hsa-miR-628-3p | 1 | 3 |
| 512 | hsa-miR-3144-3p | 1 | 3 |
| 513 | hsa-let-7c-3p | 1 | 3 |
| 514 | hsa-miR-4781-3p | 1 | 3 |
| 515 | hsa-miR-5683 | 1 | 3 |
| 516 | hsa-miR-211-5p | 1 | 3 |
| 517 | hsa-miR-129-5p | 1 | 3 |
| 518 | hsa-miR-491-5p | 1 | 3 |
| 519 | hsa-miR-1226-3p | 1 | 3 |
| 520 | hsa-miR-760 | 1 | 3 |
| 521 | hsa-miR-1299 | 1 | 3 |
| 522 | hsa-miR-1291 | 1 | 3 |
| 523 | hsa-miR-20a-3p | 1 | 3 |
| 524 | hsa-miR-433-3p | 1 | 3 |
| 525 | hsa-miR-3688-3p | 1 | 3 |
| 526 | hsa-miR-5001-3p | 1 | 3 |
| 527 | hsa-miR-548n | 1 | 3 |
| 528 | hsa-miR-585-3p | 1 | 3 |
| 529 | hsa-miR-3619-5p | 1 | 3 |
| 530 | hsa-miR-655-3p | 1 | 3 |
| 531 | hsa-miR-4446-3p | 1 | 3 |
| 532 | hsa-miR-382-3p | 1 | 3 |
| 533 | hsa-miR-3620-3p | 1 | 3 |
| 534 | hsa-miR-627-5p | 1 | 3 |
| 535 | hsa-miR-588 | 1 | 3 |
| 536 | hsa-miR-627-3p | 1 | 3 |
| 537 | hsa-miR-3679-5p | 1 | 3 |
| 538 | hsa-miR-301a-5p | 1 | 3 |
| 539 | hsa-miR-543 | 1 | 3 |
| 540 | hsa-miR-4682 | 1 | 3 |
| 541 | hsa-miR-454-5p | 1 | 3 |
| 542 | hsa-miR-3622b-3p | 1 | 3 |
| 543 | hsa-miR-2110 | 1 | 3 |
| 544 | hsa-miR-3605-3p | 1 | 3 |
| 545 | hsa-miR-590-5p | 1 | 3 |
| 546 | hsa-miR-223-5p | 1 | 3 |
| 547 | hsa-miR-654-5p | 1 | 3 |
| 548 | hsa-miR-219a-5p | 1 | 3 |
| 549 | hsa-miR-3194-5p | 1 | 3 |
| 550 | hsa-miR-486-3p | 1 | 3 |
| 551 | hsa-miR-3136-5p | 1 | 3 |
| 552 | hsa-miR-5091 | 1 | 3 |
| 553 | hsa-miR-545-5p | 1 | 3 |
| 554 | hsa-miR-6842-3p | 1 | 3 |
| 555 | hsa-miR-376a-5p | 1 | 3 |
| 556 | hsa-miR-548f-3p | 1 | 3 |
| 557 | hsa-miR-6516-3p | 1 | 3 |
