# Supplementary material for: Genome-wide profiling of the PIWI-interacting RNA-mRNA regulatory networks in epithelial ovarian cancers
Source: PLoS One. 2018 Jan 10;13(1):e0190485. doi: 10.1371/journal.pone.0190485 (PMC5761873; doi:10.1371/journal.pone.0190485)
Supplement: S3 Table — (DOCX) [file pone.0190485.s003.docx]

Table S3. Abundance of t-RNA derived piRNA in each sample

| **tRNA species** | **piRNA mapped to in ENOCa** | **piRNA mapped to in SOCa** | **piRNA mapped to in Normal Ovary** |
| --- | --- | --- | --- |
| **tRNA-Ala GCG/GCY/AGC/CGC/TGC** | Yes | Yes | Yes |
| **tRNA-Arg-AGA/AGG/CGG//ACG/CCG//CCT/TCG/CGY** | Yes | Yes | Yes |
| **tRNA-Asn-AAC/GTT** | Yes | Yes | Yes |
| **tRNA-Ile-ATT/AAT** | Yes | Yes | Yes |
| **tRNA-Val-AAC/CAC** | Yes | Yes | Yes |
| **tRNA-Tyr-GTA** | Yes | Yes | Yes |
| **tRNA-SeC-TGA/TCA** | Yes | Yes | Yes |
| **tRNA-Thr-CGT** | Yes | Yes | Yes |
| **tRNA-Asp-GAY** | Yes | Yes | Yes |
| **tRNA-Glu-GAG** | Yes | Yes | Yes |
| **tRNA-Leu-TTA/CTA** | Yes | Yes | Yes |
| **tRNA-Gly-GGA/GCC/GGY** | Yes | Yes | Yes |
| **tRNA-Pro-CCA/TGG** | Yes | Yes | Yes |
| **tRNA-Ser-AGY/TGA/TCA/TCYGCT/AGA** | Yes | Yes | Yes |
| **tRNA-Met-CAT** | Yes | Yes | Yes |
| **tRNA-Gln-CAA/CAG/CTG** | Yes | Yes | Yes |
| **tRNA-Lys-AAA/CTT/AAG** | Yes | Yes | Yes |
| **tRNA-Cys-TGY** | Yes | Yes | No |
