# Supplementary material for: Genome-wide profiling of the PIWI-interacting RNA-mRNA regulatory networks in epithelial ovarian cancers
Source: PLoS One. 2018 Jan 10;13(1):e0190485. doi: 10.1371/journal.pone.0190485 (PMC5761873; doi:10.1371/journal.pone.0190485)
Supplement: S4 Table — (DOCX) [file pone.0190485.s004.docx]

Table S4A. Differentially expressed up-regulated piRNAs in ENOCa

| **S. No** | **NCBI Accession** | **Up-regulated piRNA in ENOCa** | **Fold-change** |
| --- | --- | --- | --- |
| 1 | DQ570994 | piR-31106 | 46.2 |
| 2 | DQ596932 | piR-34998 | 18.48 |
| 3 | DQ584197 | piR-51309 | 12.32 |
| 4 | DQ585095 | piR-52207 | 11.07 |
| 5 | DQ596531 | piR-34597 | 9.87 |
| 6 | DQ585854 | piR-52966 | 9.24 |
| 7 |  | piR-49124 | 8.3 |
| 8 | DQ598273 | piR-36339 | 7.01 |
| 9 | DQ593303 | piR-33415 | 6.23 |
| 10 | DQ576606 | piR-44718 | 6.16 |
| 11 | DQ597971 | piR-36037 | 6.16 |
| 12 | DQ598294 | piR-36360 | 6.16 |
| 13 | DQ591997 | piR-59109 | 6.16 |
| 14 | DQ578516 | piR-46628 | 6.16 |
| 15 | DQ573323 | piR-41435 | 6.16 |
| 16 | DQ596805 | piR-34871 | 5.3 |
| 17 | DQ597997 | piR-36063 | 4.83 |
| 18 | DQ596723 | piR-34789 | 4.78 |
| 19 | DQ598016 | piR-36082 | 4.67 |
| 20 | DQ593671 | piR-33783 | 4.44 |
| 21 | DQ593356 | piR-33468 | 4.3 |
| 22 | DQ598675 | piR-36741 | 4.24 |
| 23 | DQ598312 | piR-36378 | 4.06 |
| 24 | DQ570812 | piR-30924 | 4.04 |
| 25 | DQ598641 | piR-36707 | 4.04 |
| 26 | DQ570968 | piR-31080 | 3.81 |
| 27 | DQ598433 | piR-36499 | 3.58 |
| 28 | DQ598104 | piR-36170 | 3.48 |
| 29 | DQ597974 | piR-36040 | 3.45 |
| 30 | DQ578783 | piR-46895 | 3.12 |
| 31 | DQ593374 | piR-33486 | 3.11 |
| 32 | DQ598263 | piR-36329 | 3 |
| 33 | DQ594556 | piR-60668 | 2.93 |
| 34 | DQ571031 | piR-31143 | 2.92 |
| 35 | DQ590835 | piR-57947 | 2.75 |
| 36 | DQ593270 | piR-33382 | 2.71 |
| 37 | DQ570117 | piR-30229 | 2.68 |
| 38 | DQ598167 | piR-36233 | 2.68 |
| 39 | DQ598274 | piR-36340 | 2.66 |
| 40 | DQ570926 | piR-31038 | 2.61 |
| 41 | DQ595536 | piR-61648 | 2.58 |
| 42 | DQ593752 | piR-33864 | 2.56 |
| 43 | DQ570940 | piR-31052 | 2.52 |
| 44 | DQ575661 | piR-43773 | 2.488 |
| 45 | DQ570326 | piR-30438 | 2.39 |
| 46 | DQ576608 | piR-44720 | 2.34 |
| 47 | DQ593414 | piR-33526 | 2.34 |
| 48 | DQ575658 | piR-43770 | 2.32 |
| 49 | DQ580854 | piR-48966 | 2.29 |
| 50 | DQ584545 | piR-51657 | 2.29 |
| 51 | DQ571030 | piR-31142 | 2.28 |
| 52 | DQ585617 | piR-52729 | 2.18 |
| 53 | DQ575492 | piR-43604 | 2.14 |
| 54 | DQ594461 | piR-60573 | 2.08 |
| 55 | DQ598445 | piR-36511 | 2.05 |
| 56 | DQ596538 | piR-34604 | 1.93 |
| 57 | DQ597238 | piR-35304 | 1.91 |
| 58 | DQ597482 | piR-35548 | 1.86 |
| 59 | DQ596183 | piR-34249 | 1.84 |
| 60 | DQ591357 | piR-58469 | 1.83 |
| 61 | DQ570513 | piR-30625 | 1.80 |
| 62 | DQ575882 | piR-43994 | 1.76 |
| 63 | DQ597485 | piR-35551 | 1.75 |
| 64 | DQ575884 | piR-43996 | 1.704 |
| 65 | DQ597403 | piR-35469 | 1.68 |
| 66 | DQ584904 | piR-52016 | 1.68 |
| 67 | DQ573097 | piR-41209 | 1.65 |
| 68 | DQ571823 | piR-31935 | 1.64 |
| 69 | DQ570778 | piR-30890 | 1.62 |
| 70 | DQ592970 | piR-33082 | 1.61 |
| 71 | DQ598159 | piR-36225 | 1.58 |
| 72 | DQ596992 | piR-35058 | 1.55 |
| 73 | DQ570814 | piR-30926 | 1.55 |
| 74 | DQ596745 | piR-34811 | 1.54 |

Table S4B. Differentially expressed down-regulated piRNAs in ENOCa

| **S. No** | **NCBI Accession** | **Down regulated piRNA** | **Fold-change** |
| --- | --- | --- | --- |
| 1 | DQ596310 | piR-34376 | -65.49 |
| 2 | DQ593574 | piR-33686 | -12.74 |
| 3 | DQ596308 | piR-34374 | -24.08 |
| 4 | DQ596309 | piR-34375 | -210.18 |
| 5 | DQ596311 | piR-34377 | -100.48 |
| 6 | DQ583674 | piR-50786 | -2.97 |
| 7 | DQ593325 | piR-33437 | -1.54 |
| 8 | DQ583273 | piR-50385 | -2.97 |
| 9 | DQ595023 | piR-61135 | -6.74 |
| 10 | DQ597218 | piR-35284 | -3.32 |
| 11 | DQ588101 | piR-55213 | -4.81 |
| 12 | DQ589209 | piR-56321 | -2.97 |
| 13 | DQ598650 | piR-36716 | -4.81 |
| 14 | DQ570001 | piR-30113 | -5.97 |
| 15 | DQ571813 | piR-31925 | -2.33 |
| 16 | DQ575660 | piR-43772 | -1.63 |
| 17 | DQ592953 | piR-33065 | -2.97 |
| 18 | DQ588910 | piR-56022 | -2.97 |
| 19 | DQ596292 | piR-34358 | -2.97 |
| 20 | DQ577259 | piR-45371 | -7.06 |
| 21 | DQ596602 | piR-34668 | -5.93 |
| 22 | DQ596603 | piR-34669 | -2.97 |
| 23 | DQ596377 | piR-34443 | -1.93 |
| 24 | DQ570956 | piR-31068 | -4.75 |
| 25 | DQ591007 | piR-58119 | -2.97 |
| 26 | DQ597972 | piR-36038 | -2.05 |
| 27 | DQ572870 | piR-40982 | -4.11 |
| 28 | DQ593538 | piR-33650 | -2.81 |
| 29 | DQ596467 | piR-34533 | -51.2 |
| 30 | DQ593375 | piR-33487 | -2.36 |
| 31 | DQ570698 | piR-30810 | -2.63 |
| 32 | DQ597887 | piR-35953 | -2.97 |
| 33 | DQ597886 | piR-35952 | -2.25 |
| 34 | DQ570206 | piR-30318 | -6.9 |
| 35 | DQ597960 | piR-36026 | -2.97 |
| 36 | DQ600079 | piR-38145 | -5.93 |
| 37 | DQ570392 | piR-30504 | -2.97 |
| 38 | DQ570394 | piR-30506 | -682.21 |
| 39 | DQ598375 | piR-36441 | -1.93 |
| 40 | DQ570772 | piR-30884 | -2.97 |
| 41 | DQ571851 | piR-31963 | -2.97 |
| 42 | DQ590872 | piR-57984 | -2.97 |
| 43 | DQ596314 | piR-34380 | -2.97 |
| 44 | DQ571875 | piR-31987 | -7.7 |
| 45 | DQ569993 | piR-30105 | -8.9 |
| 46 | DQ571003 | piR-31115 | -1.8 |
| 47 | DQ594126 | piR-60238 | -24.59 |
| 48 | DQ596306 | piR-34372 | -1.65 |
| 49 | DQ576200 | piR-44312 | -2.48 |
| 50 | DQ575064 | piR-43176 | -3.18 |
| 51 | DQ598428 | piR-36494 | -4.17 |
| 52 | DQ593744 | piR-33856 | -1.66 |
| 53 | DQ597341 | piR-35407 | -3.77 |
| 54 | DQ571538 | piR-31650 | -7.45 |
| 55 | DQ586430 | piR-53542 | -1.83 |
| 56 | DQ596670 | piR-34736 | -6.54 |
| 57 | DQ593275 | piR-33387 | -2.40 |
| 58 | DQ589338 | piR-56450 | -5.12 |
| 59 | DQ571500 | piR-31612 | -2.16 |
| 60 | DQ596587 | piR-34653 | -4.82 |
| 61 | DQ571591 | piR-31703 | -5.59 |
| 62 | DQ595807 | piR-61919 | -14.76 |
| 63 | DQ598646 | piR-36712 | -19.73 |
| 64 | DQ598651 | piR-36717 | -2.42 |
| 65 | DQ593636 | piR-33748 | -4.95 |
| 66 | DQ570728 | piR-30840 | -4.24 |
| 67 | DQ572892 | piR-41004 | -8.1 |
| 68 | DQ571511 | piR-31623 | -2.3 |
| 69 | DQ571858 | piR-31970 | -7.11 |
| 70 | DQ596390 | piR-34456 | -424.69 |
| 71 | DQ596863 | piR-34929 | -11.86 |
| 72 | DQ596225 | piR-34291 | -339.95 |
| 73 | DQ598272 | piR-36338 | -2.31 |
| 74 | DQ571335 | piR-31447 | -3.27 |
| 75 | DQ571419 | piR-31531 | -3 |
| 76 | DQ596354 | piR-34420 | -65.49 |
| 77 | DQ571256 | piR-31368 | -2.41 |

Table S4C. Differentially expressed up-regulated piRNAs in SOCa

| **S. No.** | **NCBI Accession** | **Up-Regulated piRNA** | **Fold-Change** |
| --- | --- | --- | --- |
| 1 | DQ570994 | piR-31106 | 72.62 |
| 2 | DQ593303 | piR-33415 | 19.5 |
| 3 | DQ596470 | piR-34536 | 13.45 |
| 4 | DQ596932 | piR-34998 | 13.45 |
| 5 | DQ598273 | piR-36339 | 8.16 |
| 6 | DQ591357 | piR-58469 | 8.07 |
| 7 | DQ593621 | piR-33733 | 8.07 |
| 8 | DQ593837 | piR-33949 | 8.07 |
| 9 | DQ598641 | piR-36707 | 6.35 |
| 10 | DQ590835 | piR-57947 | 6.09 |
| 11 | DQ570999 | piR-31111 | 5.43 |
| 12 | DQ597945 | piR-36011 | 5.41 |
| 13 | DQ596602 | piR-34668 | 4.99 |
| 14 | DQ597997 | piR-36063 | 4.95 |
| 15 | DQ581012 | piR-49124 | 4.53 |
| 16 | DQ576608 | piR-44720 | 4.08 |
| 17 | DQ594461 | piR-60573 | 3.63 |
| 18 | DQ596276 | piR-34342 | 3.62 |
| 19 | DQ594556 | piR-60668 | 3.49 |
| 20 | DQ570814 | piR-30926 | 3.47 |
| 21 | DQ593358 | piR-33470 | 3.32 |
| 22 | DQ598433 | piR-36499 | 3.32 |
| 23 | DQ598428 | piR-36494 | 3.29 |
| 24 | DQ595536 | piR-61648 | 3.29 |
| 25 | DQ587795 | piR-54907 | 3.29 |
| 26 | DQ593671 | piR-33783 | 3.27 |
| 27 | DQ597403 | piR-35469 | 3.19 |
| 28 | DQ594464 | piR-60576 | 3.11 |
| 29 | DQ598107 | piR-36173 | 3 |
| 30 | DQ598640 | piR-36706 | 2.99 |
| 31 | DQ593325 | piR-33437 | 2.95 |
| 32 | DQ593414 | piR-33526 | 2.95 |
| 33 | DQ593752 | piR-33864 | 2.9 |
| 34 | DQ598675 | piR-36741 | 2.8 |
| 35 | DQ575661 | piR-43773 | 2.63 |
| 36 | DQ595023 | piR-61135 | 2.59 |
| 37 | DQ596745 | piR-34811 | 2.54 |
| 38 | DQ598159 | piR-36225 | 2.52 |
| 39 | DQ585617 | piR-52729 | 2.40 |
| 40 | DQ573097 | piR-41209 | 2.25 |
| 41 | DQ596723 | piR-34789 | 2.21 |
| 42 | DQ593424 | piR-33536 | 2.11 |
| 43 | DQ593270 | piR-33382 | 2.1 |
| 44 | DQ579193 | piR-47305 | 1.92 |
| 45 | DQ570513 | piR-30625 | 1.86 |
| 46 | DQ582199 | piR-32311 | 1.84 |
| 47 | DQ595807 | piR-61919 | 1.84 |
| 48 | DQ596805 | piR-34871 | 1.82 |
| 49 | DQ596992 | piR-35058 | 1.70 |
| 50 | DQ585095 | piR-52207 | 1.67 |
| 51 | DQ596155 | piR-34221 | 1.66 |
| 52 | DQ598167 | piR-36233 | 1.65 |
| 53 | DQ598274 | piR-36340 | 1.57 |
| 54 | DQ584545 | piR-51657 | 1.55 |
| 55 | DQ598312 | piR-36378 | 1.53 |
| 56 | DQ570968 | piR-31080 | 1.51 |

Table S4D. Differentially expressed down-regulated piRNAs in SOCa

| **S. No** | **NCBI Accession** | **Down-Regulated piRNA** | **Fold-change** |
| --- | --- | --- | --- |
| 1 | DQ596310 | piR-34376 | -9.37 |
| 2 | DQ596311 | piR-34377 | -41.84 |
| 3 | DQ596308 | piR-34374 | -7.88 |
| 4 | DQ596309 | piR-34375 | -6.98 |
| 5 | DQ593574 | piR-33686 | -3.39 |
| 6 | DQ583674 | piR-50786 | -2.97 |
| 7 | DQ583273 | piR-50385 | -2.97 |
| 8 | DQ571500 | piR-31612 | -2.47 |
| 9 | DQ597218 | piR-35284 | -8.55 |
| 10 | DQ589209 | piR-56321 | -2.97 |
| 11 | DQ585094 | piR-52206 | -5.93 |
| 12 | DQ598646 | piR-36712 | -5.27 |
| 13 | DQ598650 | piR-36716 | -2.07 |
| 14 | DQ593636 | piR-33748 | -3.61 |
| 15 | DQ576872 | piR-44984 | -1.75 |
| 16 | DQ597916 | piR-35982 | -5.93 |
| 17 | DQ571526 | piR-31638 | -2.17 |
| 18 | DQ575660 | piR-43772 | -2.19 |
| 19 | DQ597163 | piR-35229 | -5.93 |
| 20 | DQ570720 | piR-30832 | -2.97 |
| 21 | DQ588910 | piR-56022 | -2.97 |
| 22 | DQ597340 | piR-35406 | -5.93 |
| 23 | DQ596377 | piR-34443 | -2.94 |
| 24 | DQ571031 | piR-31143 | -1.82 |
| 25 | DQ570956 | piR-31068 | -5.35 |
| 26 | DQ591007 | piR-58119 | -2.97 |
| 27 | DQ570540 | piR-30652 | -1.68 |
| 28 | DQ597347 | piR-35413 | -2.68 |
| 29 | DQ593538 | piR-33650 | -4.93 |
| 30 | DQ596467 | piR-34533 | -18.52 |
| 31 | DQ582262 | piR-32374 | -2.21 |
| 32 | DQ571589 | piR-31701 | -2.44 |
| 33 | DQ593375 | piR-33487 | -1.98 |
| 34 | DQ570698 | piR-30810 | -3.31 |
| 35 | DQ597887 | piR-35953 | -2.97 |
| 36 | DQ570206 | piR-30318 | -2.08 |
| 37 | DQ600079 | piR-38145 | -5.93 |
| 38 | DQ570392 | piR-30504 | -2.97 |
| 39 | DQ570394 | piR-30506 | -84.54 |
| 40 | DQ598375 | piR-36441 | -3.31 |
| 41 | DQ570772 | piR-30884 | -2.97 |
| 42 | DQ570849 | piR-30961 | -2.13 |
| 43 | DQ571851 | piR-31963 | -2.97 |
| 44 | DQ596314 | piR-34380 | -2.97 |
| 45 | DQ571875 | piR-31987 | -1.76 |
| 46 | DQ569993 | piR-30105 | -3.31 |
| 47 | DQ594126 | piR-60238 | -7.4 |
| 48 | DQ597975 | piR-36041 | -3.46 |
| 49 | DQ575885 | piR-43997 | -1.95 |
| 50 | DQ576200 | piR-44312 | -2.60 |
| 51 | DQ597341 | piR-35407 | -4.25 |
| 52 | DQ598008 | piR-36074 | -2.63 |
| 53 | DQ597215 | piR-35281 | -3.83 |
| 54 | DQ570326 | piR-30438 | -2.13 |
| 55 | DQ575656 | piR-43768 | -1.71 |
| 56 | DQ575064 | piR-43176 | -1.71 |
| 57 | DQ570000 | piR-30112 | -2.13 |
| 58 | DQ570812 | piR-30924 | -1.80 |
| 59 | DQ571538 | piR-31650 | -4.38 |
| 60 | DQ593767 | piR-33879 | -1.51 |
| 61 | DQ596670 | piR-34736 | -6.82 |
| 62 | DQ570778 | piR-30890 | -1.56 |
| 63 | DQ570940 | piR-31052 | -1.71 |
| 64 | DQ593275 | piR-33387 | -2.87 |
| 65 | DQ580854 | piR-48966 | -1.57 |
| 66 | DQ589338 | piR-56450 | -1.92 |
| 67 | DQ572892 | piR-41004 | -3.7 |
| 68 | DQ571419 | piR-31531 | -5.22 |
| 69 | DQ596587 | piR-34653 | -3.98 |
| 70 | DQ598651 | piR-36717 | -40.8 |
| 71 | DQ570728 | piR-30840 | -2.02 |
| 72 | DQ571858 | piR-31970 | -3.96 |
| 73 | DQ596390 | piR-34456 | -54.03 |
| 74 | DQ596863 | piR-34929 | -11.86 |
| 75 | DQ596225 | piR-34291 | -18.54 |
| 76 | DQ587153 | piR-54265 | -1.7 |
| 77 | DQ593744 | piR-33856 | -2.02 |
| 78 | DQ571335 | piR-31447 | -7.11 |
| 79 | DQ597886 | piR-35952 | -15.16 |
| 80 | DQ596354 | piR-34420 | -7.5 |
| 81 | DQ571256 | piR-31368 | -5.51 |
