# Supplementary material for: Genome-wide profiling of the PIWI-interacting RNA-mRNA regulatory networks in epithelial ovarian cancers
Source: PLoS One. 2018 Jan 10;13(1):e0190485. doi: 10.1371/journal.pone.0190485 (PMC5761873; doi:10.1371/journal.pone.0190485)
Supplement: S5 Table — (DOCX) [file pone.0190485.s005.docx]

Table S5A. List of target genes harboring SINE elements which is targeted by differentially expressed up-regulated piR-52207 in ENOCa.

| **S.No** | **Target Genes** | **Corresponding Transcripts** | **piRNA ID** | **TBindPos** | **TargetBindReg** |
| --- | --- | --- | --- | --- | --- |
| 1 | S100PBP | NM_001256121.1 | piR-52207 (DQ585095) | 2893-2922 | 3'UTR |
|  |  | NM_022753.3 | piR-52207(DQ585095) | 2907-2936 | 3'UTR |
| 2 | ZMYM6 | NM_007167.3 | piR-52207(DQ585095) | 4699-4728 | 3'UTR |
| 3 | MTR | NM_000254.2 | piR-52207 (DQ585095) | 5093-5122 | 3'UTR |
| 4 | FAM114A1 | NM_138389.2 | piR-52207 (DQ585095) | 2579-2608 | 3'UTR |
| 5 | WAC | NM_100264.2 | piR-52207 (DQ585095) | 4711-4740 | 3'UTR |
|  |  | NM_016628.4 | piR-52207 (DQ585095) | 4420-4449 | 3'UTR |
|  |  | NM_100486.3 | piR-52207 (DQ585095) | 4111-4140 | 3'UTR |
| 6 | NUDT4 | NM_199040.3 | piR-52207 (DQ585095) | 2293-2322 | 3'UTR |
|  |  | NM_019094.5 | piR-52207 (DQ585095) | 2290-2319 | 3'UTR |
| 7 | MPHOSPH8 | NM_017520.3 | piR-52207 (DQ585095) | 3070-3099 | 3'UTR |
| 8 | ACTR10 | NM_018477.2 | piR-52207 (DQ585095) | 1741-1770 | 3'UTR |
| 9 | JOSD1 | NM_014876.5 | piR-52207 (DQ585095) | 1758-1787 | 3'UTR |
| 10 | EIF2S3 | NM_001415.3 | piR-52207 (DQ585095) | 1840-1869 | 3'UTR |

Table S5B. List of target genes harboring SINE elements which is targeted by differentially expressed up-regulated piR-52207 in SOCa.

| **S.No** | **Target Genes** | **Corresponding Transcripts** | **piRNA ID** | **TBindPos** | **TargetBindReg** |
| --- | --- | --- | --- | --- | --- |
| 1 | ACTR10 | NM_018477.2 | piR-52207 (DQ585095) | 1741-1770 | 3'UTR |
| 2 | C2CD2 | NM_015500.1 | piR-52207 (DQ585095) | 4576-4605 | 3'UTR |
|  |  | NM_199050.2 | piR-52207 (DQ585095) | 4075-4104 | 3'UTR |
| 3 | LIAS | NM_006859.3 | piR-33733 (DQ593621) | 1398-1427 | 3'UTR |
|  |  | NM_194451.2 | piR-33733 (DQ593621) | 1286-1315 | 3'UTR |
| 4 | MPHOSPH8 | NM_017520.3 | piR-52207 (DQ585095) | 3070-3099 | 3'UTR |
| 5 | PLEKHA5 | NM_001143821.2 | piR-52207 (DQ585095) | 4052-4081 | 3'UTR |
| 6 | TMEM159 | NM_020422.5 | piR-52207 (DQ585095) | 1854-1883 | 3'UTR |
| 7 | TMX4 | NM_021156.3 | piR-52207 (DQ585095) | 3877-3906 | 3'UTR |
